# The *Chlamydia*-related *Waddlia chondrophila* encodes functional type II toxin-antitoxin systems

**DOI:** 10.1128/aem.00681-23

**Published:** 2024-01-12

**Authors:** Silvia Ardissone, Gilbert Greub

**Affiliations:** 1 Institute of Microbiology, Lausanne University Hospital and Lausanne University, Lausanne, Switzerland; Washington University in St. Louis, St. Louis, Missouri, USA

**Keywords:** toxin-antitoxin, *Waddlia chondrophila*, Chlamydiales, intracellular bacteria, virulence factors

## Abstract

Bacterial toxin-antitoxin (TA) systems are widespread in chromosomes and plasmids of free-living microorganisms, but only a few have been identified in obligate intracellular species. We found seven putative type II TA modules in *Waddlia chondrophila*, a *Chlamydia*-related species that is able to infect a very broad series of eukaryotic hosts, ranging from protists to mammalian cells. The RNA levels of *Waddlia* TA systems are significantly upregulated by iron starvation and novobiocin, but they are not affected by antibiotics such as β-lactams and glycopeptides, which suggests different mechanisms underlying stress responses. Five of the identified TA modules, including HigBA1 and MazEF1, encoded on the *Waddlia* cryptic plasmid, proved to be functional when expressed in a heterologous host. TA systems have been associated with the maintenance of mobile genetic elements, bacterial defense against bacteriophages, and persistence upon exposure to adverse conditions. As their RNA levels are upregulated upon exposure to adverse conditions, *Waddlia* TA modules may be involved in survival to stress. Moreover, as *Waddlia* can infect a wide range of hosts including free-living amoebae, TA modules could also represent an innate immunity system to fight against bacteriophages and other microorganisms with which *Waddlia* has to share its replicative niche.

**IMPORTANCE:**

The response to adverse conditions, such as exposure to antibiotics, nutrient starvation and competition with other microorganisms, is essential for the survival of a bacterial population. TA systems are modules composed of two elements, a toxic protein and an antitoxin (protein or RNA) that counteracts the toxin. Although many aspects of TA biological functions still await to be elucidated, TAs have often been implicated in bacterial response to stress, including the response to nutrient starvation, antibiotic treatment and bacteriophage infection. TAs are ubiquitous in free-living bacteria but rare in obligate intracellular species such as chlamydiae. We identified functional TA systems in *Waddlia chondrophila*, a chlamydial species with a strikingly broad host range compared to other chlamydiae. Our work contributes to understand how obligate intracellular bacteria react to adverse conditions that might arise from competition with other viruses/bacteria for the same replicative niche and would threaten their ability to replicate.

## INTRODUCTION

Toxin-antitoxin (TA) systems are small genetic elements encoding a stable toxin that can poison the producer cell by blocking essential functions such as translation or membrane integrity, and an unstable antitoxin that counteracts the toxin activity. TA modules are currently classified in eight classes, depending on the antitoxin nature (protein or RNA) and mode of action (e.g., direct binding to the toxin, competition for the same target, and block of toxin translation) (1). Most toxins are proteins, with the exception of the recently identified type VIII toxins that are small RNAs (2, 3). TA modules have been initially identified on plasmids and considered implicated in plasmid maintenance (4, 5). As the toxin is more stable than the antitoxin, if the plasmid is lost, the antitoxin is degraded faster than the toxin, which then kills the cell. However, TA systems are widespread in bacteria, with some species possessing dozens of them, mainly encoded on the chromosome (1, 6–8). Their function is still controversial. They have been associated with persistence and antibiotic resistance, as transcription of TA genes is frequently upregulated in response to a stress such as DNA damage (SOS response) or to nutrient limitation (stringent response) (9–16). Upon stress, the activation of the toxin would block the bacterial growth, leading to a state of dormancy, waiting for conditions more favorable to growth. Recently, this role has been debated, as it was shown that abiotic stress can induce a dramatic increase in TA transcription without generating a critical, growth-inhibitory level of free active toxin (17). Moreover, deletion of TA modules from a genome does not always affect persisters formation (18, 19). On the other hand, the observation that TA modules are often encoded on prophages and are frequently part of the non-conserved accessory genome of a species has suggested a role for TA systems in defense against bacteriophages [reviewed in reference (20)].

Type II TA modules are the most common and the best characterized modules. In this case, both the toxin and the antitoxin are proteins, encoded in operon, and the antitoxin can directly bind to the toxin, inhibiting its toxicity (21–25). Type II antitoxins are more susceptible than their cognate toxins to proteolysis by cellular proteases such as Lon and ClpP (26), and in addition to the toxin-binding region, they frequently possess a DNA-binding domain that enables them to repress their own promoter, alone or in complex with the cognate toxin (27). The main target of type II toxins is protein synthesis: numerous members of this group are RNases that cleave target mRNAs, tRNAs or rRNA with different degrees of specificity (28–31).

Although widespread in free-living bacteria, TA modules are considered rare in obligate intracellular bacteria, as these microorganisms possess small genomes adapted to their specific niche as a result of genome size reduction with substantial gene loss (8). Moreover, strict intracellular bacteria are considered somehow protected from phages when internalized in eukaryotic cells. Nevertheless, TA systems have been identified in the genomes of obligate intracellular bacteria including several *Rickettsia* and *Wolbachia* species (8, 32–34). Conversely, no TA system has been found until now in the members of the Chlamydiaceae, the best-studied family of the Chlamydiales order that includes several well-known human and animal pathogens such as *Chlamydia trachomatis*, *Chlamydia pneumoniae*, and *Chlamydia psittaci*. The Chlamydiales order represents, however, a large group of obligate intracellular bacteria and includes a constantly growing number of families in addition to the Chlamydiaceae, collectively known as *Chlamydia*-related, encompassing numerous species able to infect a wide host range (35, 36). TA modules are actually encoded in the genome of *Chlamydia*-related species such as *Waddlia chondrophila* (hereafter *Waddlia*) (37), a member of the Waddliaceae family that was first isolated from an aborted bovine fetus (38), and *Estrella lausannensis* (39), a member of the Criblamydiaceae family that was isolated from river water (40). Both *Waddlia* and *E. lausannensis* can infect a wide variety of hosts including mammalian cells, fish cells, insect cells, as well as amoebae (41–45). Moreover, *Waddlia* and *E. lausannensis* share with all known members of the Chlamydiales a common biphasic developmental cycle, characterized by the infectious, non-replicative elementary bodies (EBs) and the replicative, non-infectious reticulate bodies (RBs), which are morphologically and functionally distinct (46–48). EBs enter the host cell and reside in a compartment called inclusion, surrounded by a membrane, where they differentiate into RBs and proliferate. After several rounds of replication, RBs re-differentiate into EBs, which are released into the extracellular environment and can start a new infection cycle. If exposed to stressful conditions, such as antibiotics or nutrient deprivation, chlamydiae can differentiate into the so-called aberrant bodies (ABs), a non-replicative form that has been associated with persistence (49–54). ABs are usually defined as enlarged bacteria in which DNA replication occurs without cell division (55), but it has recently been shown that, at least in the case of *Waddlia*, they can be morphologically different, depending on the type of stress that has caused them (53).

*Chlamydia*-related bacteria also feature larger genomes compared to the Chlamydiaceae (e.g., *Waddlia* has a chromosome that is about double in size compared to the chromosome of *C. trachomatis*), indicating that *Chlamydia*-related species conserve metabolic functions that are lost in the Chlamydiaceae family, which could at least partially explain the broader host-range of the former (37). TA-encoding genes are also among the functions that are present in *Chlamydia*-related but absent in Chlamydiaceae species (8, 37, 39). However, the distribution of TA modules in *Chlamydia*-related microorganisms, as well as their role in obligate intracellular bacteria, remains to be elucidated. We recently identified three type II TA modules that are strongly up regulated in *Waddlia* ABs induced by iron starvation (56). In this work, we aimed to identify type II TA modules in the *Waddlia* genome and to assess their role in ABs induced by different types of stress. Moreover, we investigated whether they encode functional type II toxins by expressing them in a heterologous host, *Escherichia coli*, which allowed the identification of functional HigBA and MazEF modules encoded on the *Waddlia* plasmid (pWc), as well as HigBA and MazEF modules encoded on the chromosome.

## RESULTS

### *Waddlia* genome encodes several putative type II toxin-antitoxin modules

We recently identified three putative type II toxins among the most (fold change >4) upregulated genes in *Waddlia* ABs formed upon iron starvation (56). Two of them are encoded on the pWc plasmid: p0002 has homology to HigB and p0022 shows homology to MazF. Both HigB and MazF are toxins with endoribonuclease activity. We thus checked whether putative antitoxins are encoded next to p0002 and p0022. A hypothetical protein (p0003) with structural similarity to DNA-binding proteins (Table S1) is encoded downstream and in operon with *p0002*. HigA antitoxins are known to bind DNA to repress their own promoter, and the unusual genetic arrangement (with the toxin upstream of the antitoxin) is quite common for the HigBA modules. Therefore, p0003 is a good candidate as putative HigA. On the other hand *p0021*, the gene upstream and in operon with *p0022*, encodes a protein with structural homology to MazE antitoxin from *Mycobacterium tuberculosis* (Table S1). We then wondered whether other putative type II TA modules are encoded on the *Waddlia* chromosome, as it happens for many other bacterial species. A search of the *Waddlia* chromosome for type II TA modules with TAfinder (https://bioinfo-mml.sjtu.edu.cn/TAfinder/TAfinder.php) retrieved five hits (Table 1). Wcw_1348 also has homology to HigB toxins, although it shares limited primary sequence similarity with p0002. The *wcw_1348* gene is in operon with *wcw_1347*, which encodes a protein with predicted structural similarity to HigA from *Streptococcus pneumoniae* but belongs to a different orthogroup than p0003. Thus, the *p0002-p0003* (*Waddlia higBA1*) and *wcw_1348-wcw_1347* (*Waddlia higBA2*) gene pairs were likely acquired in two separate lateral gene transfer events (Fig. S1). The *wcw_1208-wcw_1207* operon encodes another putative MazEF module that shares remarkable homology (>90% identity for both proteins) with p0021 and p0022, respectively, which suggests that the presence of two MazEF modules in *Waddlia* (MazEF1 on pWc and MazEF2 on the chromosome) is due to gene duplication (Fig. S2). The *wcw_0003-wcw_0004* operon encodes for a protein with an Arc-type ribbon-helix-helix domain (found in bacterial and phage-encoded transcriptional repressors) and a putative Doc (death on curing) protein, respectively. Finally, the *wcw_1095-wcw_1094* locus encodes a putative HicAB toxin-antitoxin pair, and the *wcw_1195-wcw_1196* operon encodes a putative DinJ antitoxin and YafQ toxin (Table 1; Table S1). Interestingly, the *hicAB* genes were also among the most upregulated by iron starvation in our transcriptomic analysis (56).

**TABLE 1:** Type II TA modules identified in *Waddlia* genome^*a*^

	Gene	Family
Plasmid		
T	*p0002*	HigB
A	*p0003*	DNA-binding prophage protein
T	*p0022*	MazF
A	*p0021*	MazE
Chromosome		
T	*wcw_0004*	Doc
A	*wcw_0003*	Arc-type ribbon-helix-helix
T	*wcw_1095*	HicA
A	*wcw_1094*	HicB
T	*wcw_1196*	YafQ
A	*wcw_1195*	DinJ
T	*wcw_1207*	MazF
A	*wcw_1208*	MazE
T	*wcw_1348*	HigB
A	*wcw_1347*	HigA/DNA-binding protein

^*a*^ The family indicated is based on annotation (https://chlamdb.ch/) (57) and/or structure prediction. Templates used by the Phyre2 web portal are listed in Table S1.

### Expression of TA-encoding genes is regulated by different stress stimuli during the infection cycle

**Fig 1:**
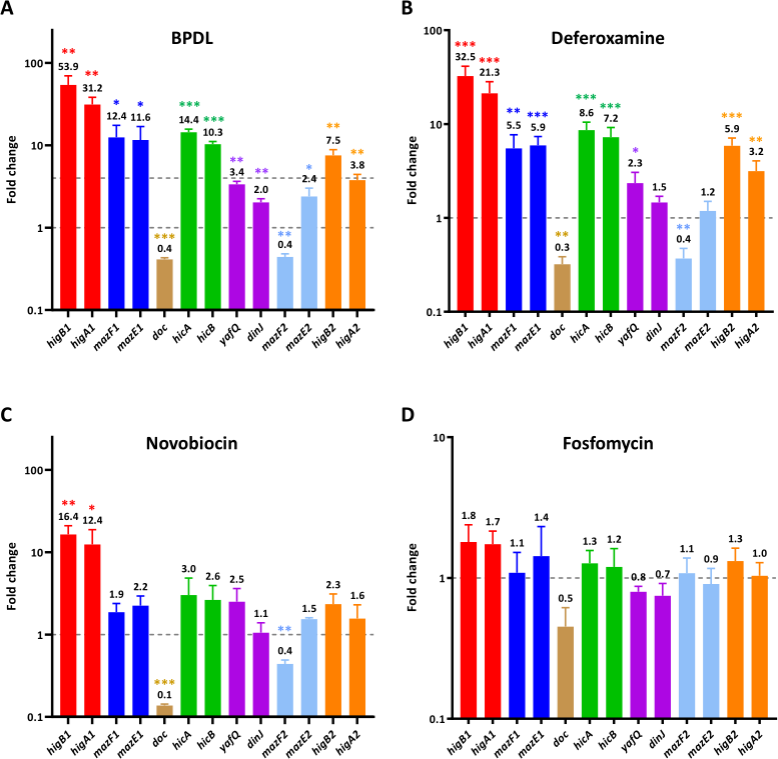
RNA levels of *Waddlia* TA modules in ABs induced by different stress stimuli. RNA levels of TA-encoding genes were analyzed by RT-qPCR in infected cells treated with BPDL (75 µM) (**A**), deferoxamine (260 µg/mL) (**B**), novobiocin (250 µg/mL) (**C**), or fosfomycin (500 µg/mL) (**D**). RNA levels were normalized according to untreated infected cells using the 16S rRNA as endogenous control. Infected cells were treated with drugs at 8 hpi and harvested at 24 hpi. Results represent the means and SDs of three independent experiments. The dashed line (at fold change = 1) represents the RNA level of each gene in the untreated sample. In the case of BPDL, the upper dashed line (at fold change = 4) corresponds to the fold change that we chose as threshold in the analysis of the RNAseq. Statistical significance was determined by paired *t*-test on ΔCt values. *P < 0.05, **P < 0.01, ***P < 0.001.

As TA modules are considered rare in obligate intracellular bacteria and have not been identified in the Chlamydiaceae so far, we wondered whether they might play a role in AB formation specifically in *Chlamydia*-related species. First, in order to confirm the transcriptional data obtained by RNA-seq, we checked the expression level of the genes encoding TA modules upon treatment with two iron chelators, 2,2′-bipyridyl (BPDL, the same used for the RNA-seq) and deferoxamine, which is also widely used for iron starvation (53, 58). As shown in Fig. 1A and B, both treatments determined an upregulation of four TA modules (*higBA1*, *mazEF1*, *hicAB*, and *higBA2*), although deferoxamine proved less efficient than BPDL to induce upregulation. The RNA levels for the other three TA modules (*mazEF2*, *dinJ-yafQ*, and *arc-doc*) did not markedly change upon iron starvation, consistent with what was observed in the RNA-seq (Fig. 1A and B). Unexpectedly, the RNA levels of *mazF2* showed a 2.5-fold decrease upon iron starvation, whereas the RNA levels of *mazE2* slightly increased. As the two genes are encoded in operon, post-transcriptional mechanisms might be responsible for the difference in RNA steady-state levels. We then wondered whether an increased expression of (some) TA modules could be a signature of all types of ABs in *Waddlia*, or if it depends on the stress applied. To investigate the transcriptional changes of TA-encoding genes upon treatment with various drugs that determine the formation of morphologically distinct ABs, as we have previously observed (53), we measured their RNA level by reverse transcription quantitative PCR (RT-qPCR). We detected a significant increase in RNA levels for the *higBA1* module upon treatment of infected cells with novobiocin (an antibiotic that targets the DNA gyrase) but no strong changes in the presence of fosfomycin, which targets MurA, the enzyme that catalyzes the first step of peptidoglycan biosynthesis from UDP-N-acetylglucosamine (Fig. 1C and D).

**Fig 2:**
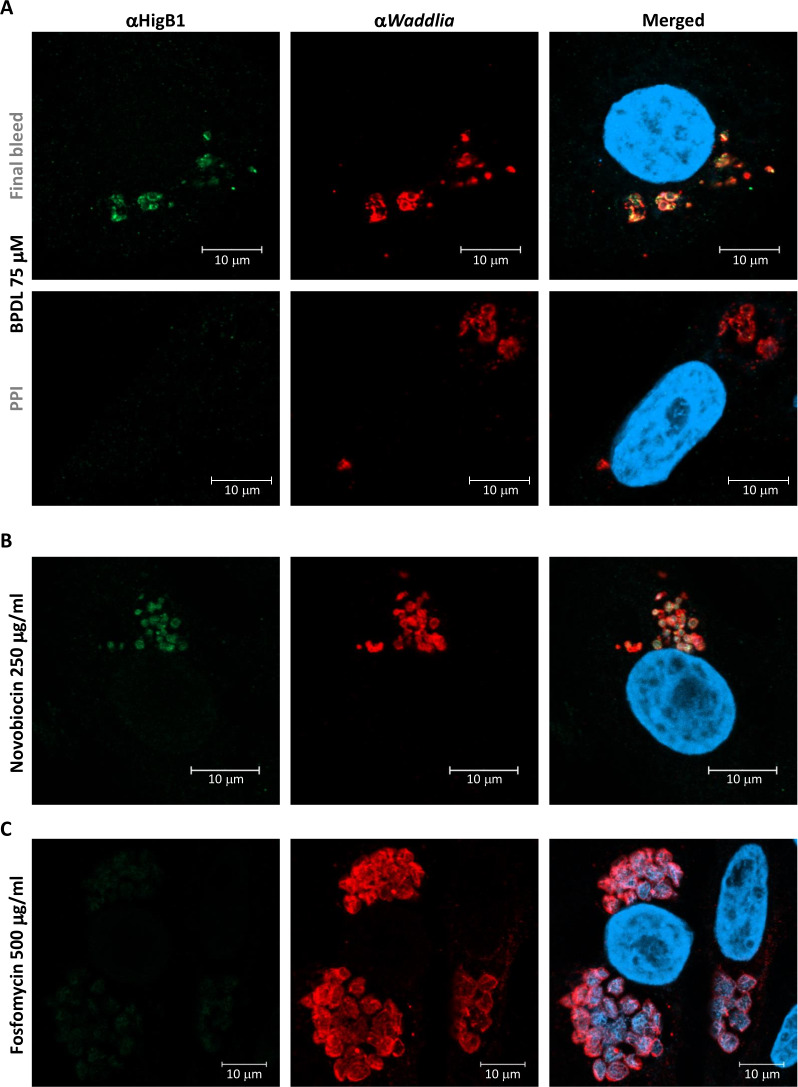
HigB1 accumulates in *Waddlia* ABs induced by exposure to the iron chelator BPDL or novobiocin. Infected cells were treated with BPDL (75 µM) (**A**), novobiocin (250 µg/mL) (**B**), or fosfomycin (500 µg/mL) (**C**) at 8 hpi and fixed 24 hpi. Samples were stained with a polyclonal antibody raised against HigB1 (green), a polyclonal antibody raised against *Waddlia* (red), and 4′,6-diamidino-2-phenylindole (blue). PPI was used with the same settings (dilution and microscope settings) as the final bleed (αHigB1). PPI, pre-immune serum.

Consistently with what was observed at the RNA level, an antibody raised against *Waddlia* HigB1 detected the protein in ABs formed in the presence of BPDL or novobiocin but not in ABs formed upon treatment with fosfomycin (Fig. 2). We tested several other drugs known to induce AB formation in *Waddlia*: three penicillins with different spectrum: penicillin G, piperacillin, and mecillinam; the β-lactamase inhibitor clavulanic acid; and two glycopeptides: teicoplanin and vancomycin (53). Only a small increase (≤3-fold) in RNA levels of the *higBA1* module was detected in presence of piperacillin or mecillinam (Fig. S3), indicating that the induction of TA modules is restricted to specific types of stress.

**Fig 3:**
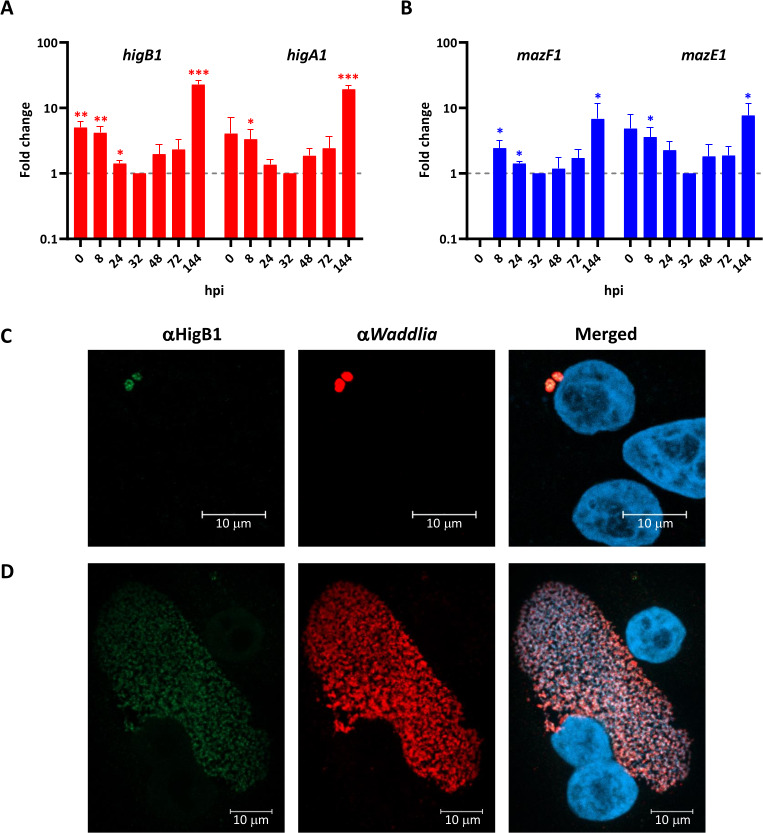
Expression of *Waddlia* HigBA1 and MazEF1 is regulated along the infection cycle. RNA levels for *higBA1* (**A**) and *mazEF1* (**B**) were analyzed by RT-qPCR in infected cells at different time points post-infection and normalized at 32 hpi (dashed line, fold change = 1) using the 16S rRNA as endogenous control. Results are the means and SDs of three independent experiments. Statistical significance was determined by paired *t*-test on δct values. *P < 0.05, **P < 0.01, ***P < 0.001. To visualize the HigB1 protein, infected cells were fixed at 8 hpi (**C**) or 24 hpi (**D**) and stained with a polyclonal antibody raised against HigB1 (green), a polyclonal antibody raised against *Waddlia* (red) and 4′,6-diamidino-2-phenylindole (blue).

We then asked whether expression of TA-encoding genes was regulated along the regular infectious cycle. We thus extracted RNA at different time points after infection of host cells and determined the expression levels of the TA genes by RT-qPCR. The RNA for the plasmid-encoded TA modules, *higBA1* and *mazEF1*, significantly accumulated in EBs compared to RBs, with a 20-fold increase at 144 h post-infection (hpi) compared to 32 hpi (the time point at which the lowest RNA levels were detected) for *higBA1* and a 7-fold increase for *mazEF1* (Fig. 3A and B). The HigB1 protein was also detected by immunofluorescence at 8 hpi (Fig. 3C), when newly differentiated RBs start to replicate, with a strong signal co-localized with that of the anti-*Waddlia* antibody. The immunofluorescence signal of HigB1 decreased in actively replicating RBs at 24 hpi (Fig. 3D), consistent with the lower levels of RNA detected by RT-qPCR. On the other hand, the RNA levels for the chromosome-encoded TA genes were rather stable along the complete infection cycle, with a peak at the beginning of the bacterial replication (8 hpi) that represented between 2.6- and 5.8-fold increases compared to 32 hpi (Fig. S4).

### Toxicity of *Waddlia* type II TA modules expressed in the heterologous host, *E. coli*

Having established that *Waddlia* TA modules are expressed and differentially regulated during growth and/or upon stress, we wanted to determine whether they are functional type II TA modules. As *Waddlia* is genetically intractable, we expressed the different toxins and antitoxins, alone or in combination, in the heterologous host, *E. coli*, from a plasmid and under control of an inducible promoter.

#### *Waddlia* MazEF modules

**Fig 4:**
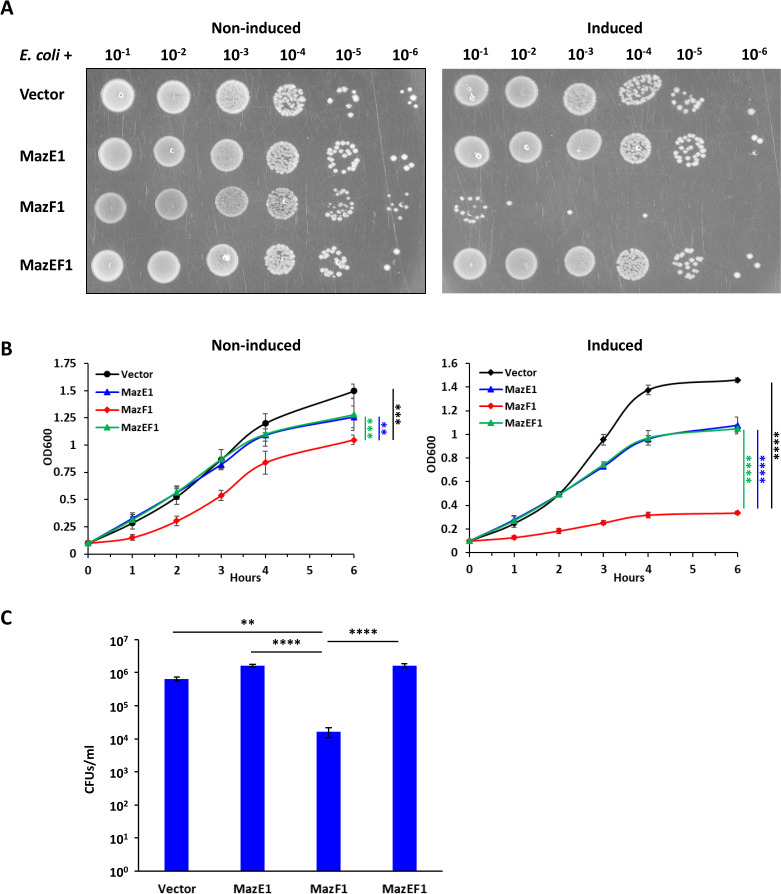
Expression of *Waddlia* MazF1 is deleterious to *E. coli. Waddlia* MazEF1 was expressed, alone or in combination, in *E. coli* under control of an arabinose-inducible promoter on plasmid. (**A**) Efficiency of plating assay showing 10-fold serial dilutions of the different *E. coli* strains on Luria-Bertani medium with and without inducer. (**B**) Growth of the same strains in liquid medium, with (right) and without (left) inducer. In the presence of arabinose, cells expressing *mazF1* grew significantly less than the other strains at all time points. (**C**) Colony-forming units (CFUs) of the different *E. coli* strains after 6-h growth in liquid medium with inducer. In panels B and C, values represent the means and SDs of three independent experiments. Statistical significance was determined by one-way analysis of variance on area under the curve (**B**) or CFU values (**C**). **P < 0.01, ***P < 0.001, ****P < 0.0001.

Expression of either *Waddlia* MazF1 or MazF2 toxins blocked *E. coli* growth on solid and liquid media and strongly reduced colony-forming units (CFUs). The growth could be restored by co-expression of MazE1 or MazE2 antitoxins (Fig. 4; Fig. S5). As the two MazEF modules share >90% identity at the amino acid level, the detrimental effect of MazF1 could be counteracted also by MazE2 and vice versa (Fig. S6A). Physical interaction between *Waddlia* MazE and MazF was assessed in *E. coli* using a bacterial two-hybrid (BACTH) assay based on the functional reconstitution of the adenylate cyclase from *Bordetella pertussis*. Reconstitution of the catalytic domain of the adenylate cyclase, composed of two fragments, T25 and T18, triggers cAMP-dependent β-galactosidase (LacZ) expression (59). We probed for interactions between *Waddlia* MazE and MazF proteins (Fig. S6B) and detected a strong increase in β-galactosidase activity with most combinations (Table S2). This indicates that both MazF toxins can interact with both MazE antitoxins, as expected from the high degree of amino acid identity and the fact that expression of MazE2 could restore growth in an *E. coli* strain expressing MazF1 and vice versa.

**Fig 5:**
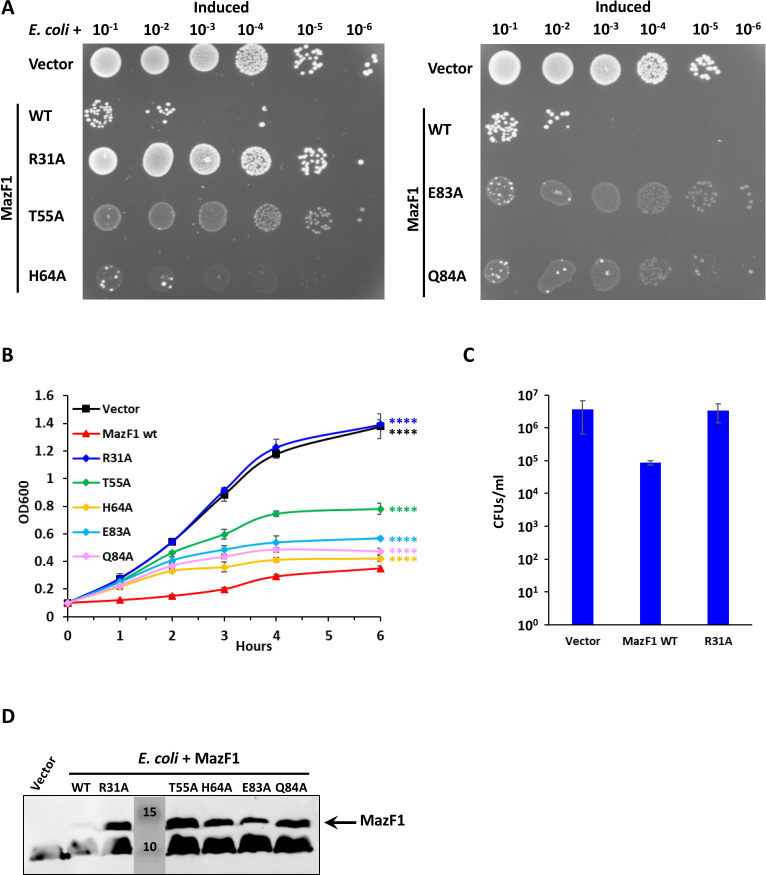
Mutation of arginine 31 strongly reduces MazF1 toxicity in *E. coli*. Different alleles of *Waddlia* MazF1 were expressed in *E. coli* under control of arabinose-inducible promoter on plasmid. (**A**) Efficiency of plating assay showing 10-fold serial dilutions of the different *E. coli* strains on Luria-Bertani medium with inducer. (**B**) Growth of the same strains in liquid medium, with inducer. Growth of cells expressing *mazF1* (R31A) is comparable to growth of cells carrying the empty vector, and *E. coli* spp. expressing the other four *mazF1* alleles also grow more than the strain expressing WT *mazF1*. Statistical significance was determined by one-way ANOVA on area under the curve. ****P < 0.0001 compared to *E. coli* expressing WT *mazF1*. (**C**) CFUs of *E. coli* carrying the empty vector, WT MazF1, or MazF1 R31A after 6-h growth in liquid medium with inducer. CFU values obtained with the empty vector and MazF1 R31A were not significantly different (one-way ANOVA test). (**D**) Immunoblot on *E. coli* expressing MazF1 variants from an arabinose-inducible promoter. Samples were taken after 6 h of induction and normalized according to the OD_600 nm_. Molecular size standards are indicated in kilodalton. For panels B and C, values represent the means and SDs of three independent experiments. Growth of the strains shown in panel A and B without inducer are shown in Fig. S7. ANOVA, analysis of variance; CFU, colony-forming unit.

In the case of MazF from *E. coli* and *Bacillus subtilis* (EcMazF and BsMazF), several residues involved in substrate binding and cleavage have been described (60, 61). Based on sequence alignment, we could identify and mutate five conserved amino acids (Fig. S2C). Arginine 31 corresponds to R25 and R29 in MazF from *B. subtilis* and *E. coli*, respectively. In EcMazF, R29 was proposed as the catalytic residue, acting as a general acid/base, and an R25A mutation completely inactivated BsMazF (60, 61). Threonine 55 corresponds to another residue essential for the activity of BsMazF, and it has been proposed to stabilize the transition state as it is close to the scissile phosphate in EcMazF (60, 61). Histidine 64, glutamate 83 and glutamine 84 correspond to three residues that in BsMazF interact with the RNA substrate and whose mutation determines a complete loss of activity (60). The five mutant alleles of *Waddlia* MazF1 showed a reduced toxicity compared to wild-type (WT) MazF1 and accumulated at substantially higher levels when expressed in *E. coli*. In particular, the R31A mutant was completely inactive, as the *E. coli* strain expressing MazF1 R31A was indistinguishable from the one carrying the empty vector in terms of growth on plate and in liquid, and the CFU phenotype was also restored (Fig. 5; Fig. S7). The same mutations were generated also in MazF2, which provided the same results as for MazF1 (Fig. S8).

#### *Waddlia* HigBA modules

**Fig 6:**
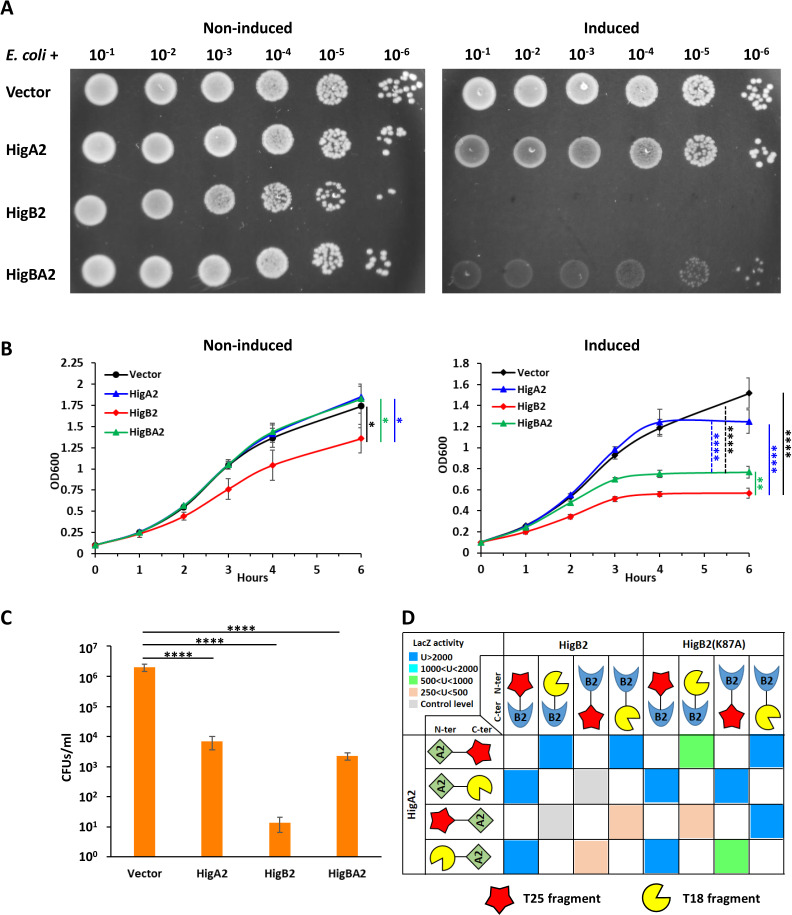
Expression of *Waddlia* HigB2 is deleterious to *E. coli. Waddlia* HigBA2 was expressed, alone or in combination, in *E. coli* under control of arabinose-inducible promoter on plasmid. (**A**) Efficiency of plating assay showing 10-fold serial dilutions of the different *E. coli* strains on LB medium with and without inducer. (**B**) Growth of the same strains in liquid medium, with (right) and without (left) inducer. In he presence of arabinose, cells expressing *higB2* grew significantly less than the other three strains; cells expressing *higBA2* grew also less than cells carrying the empty vector or *higA2* alone. (**C**) Colony-forming units of the different *E. coli* strains after 6-h growth in liquid medium with inducer. (**D**) β-Galactosidase activity, expressed in Miller units (U), of *E. coli* Δ*cyaA* expressing the combinations of HigB2 and HigA2 constructs used for the BACTH assay. Hybrids of the T25 (red star) and T18 (yellow shape) adenylate cyclase fragments with *Waddlia* HigB2 and HigA2 were created as N- and C-terminal fusions, as indicated. The level of β-galactosidase activity for each combination of constructs is indicated by the color of the squares. In panels B and C, values represent the means and SDs of three independent experiments. Statistical significance was determined by one-way ANOVA on area under the curve (**B**) or CFU values (**C**). *P < 0.05, **P < 0.01, ****P < 0.0001. Individual values of β-galactosidase activity used for panel D are listed in Table S2.

**Fig 7:**
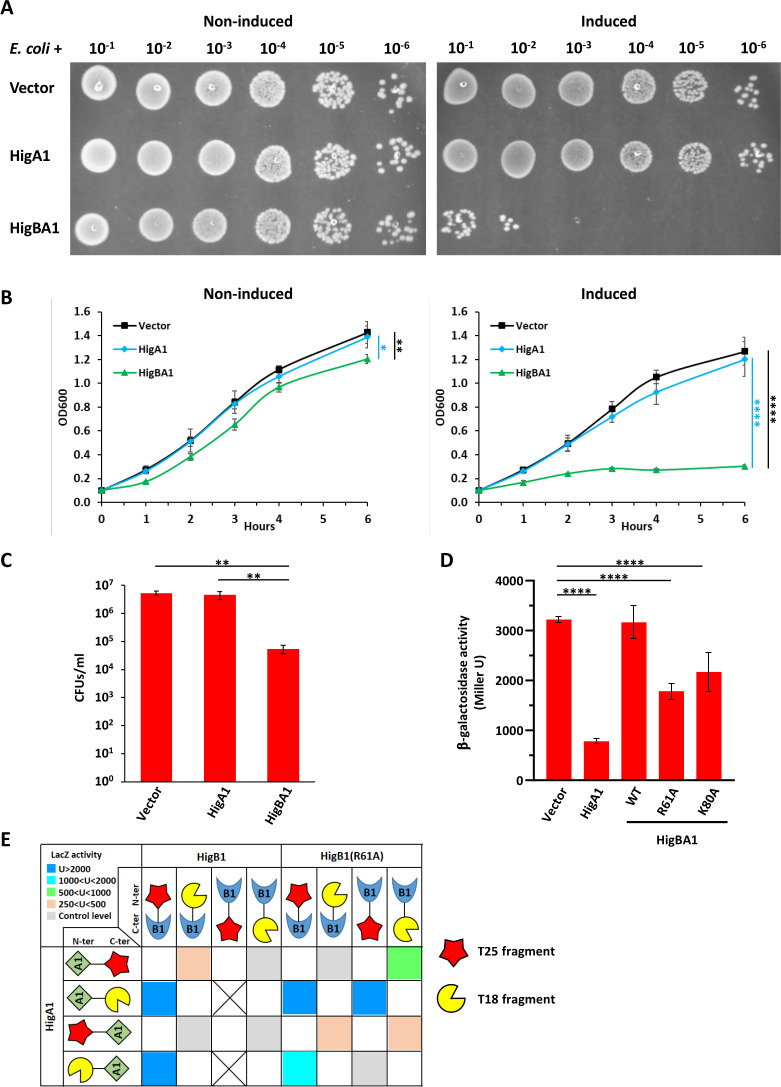
Expression of *Waddlia* HigBA1 is deleterious to *E. coli. Waddlia* HigBA1 and HigA1 were expressed in *E. coli* under control of P_ara_ on plasmid. HigA1 was expressed in *E. coli* with a V5 C-terminal tag to allow detection of the protein on immunoblot (shown in Fig. S9). (**A**) Efficiency of plating assay showing 10-fold serial dilutions of the different *E. coli* strains on Luria-Bertani medium with and without inducer. (**B**) Growth of the same strains in liquid medium, with (right) and without (left) inducer. Cells expressing *higBA1* grew significantly less than the other two strains. (**C**) Colony-forming units of the different *E. coli* strains after 6-h growth in liquid medium with inducer. In panels B and C, values represent the means and SDs of three independent experiments. (**D**) β-Galactosidase activity of the P_higBA1_ transcriptional reporter in *E. coli* cells expressing *higA1* or the *higBA1* operon under control of P_ara_. Values represent the means and SDs of three independent experiments performed with three clones each. (**E**) BACTH assay probing the interaction between *Waddlia* HigB1 and HigA1. β-Galactosidase levels are represented as in Fig. 6D. Statistical significance was determined by one-way ANOVA on area under the curve (**B**), CFU values (**C**), or Miller units (**D**). *P < 0.05, **P < 0.01, ****P < 0.0001. Individual values of β-galactosidase activity used for panel E are listed in Table S2. P_ara_*,* arabinose-inducible promoter.

Both HigB toxins from *Waddlia* appeared to be detrimental to *E. coli*. Efficiency of plating, growth in liquid, and CFUs were all strongly impaired by expression of HigB2, and these phenotypes were partially restored by co-expression of HigA2 (Fig. 6A through C). Interaction between HigA2 and HigB2 was confirmed in *E. coli* by BACTH assay (Fig. 6D). On the other hand, HigB1 could not be expressed in *E. coli* without HigA1, and expression of the HigBA1 module significantly affected *E. coli* growth (Fig. 7A through C). Due to the high toxicity of HigB1, for the BACTH assay, it was possible to detect β-galactosidase activity only with a T25-HigB1 fusion, in combination with T18-HigA1 or HigA1-T18 (Fig. 7E). As expected from the low degree of similarity between the two HigBA modules, no interaction was detected between HigB1 and HigA2 or between HigB2 and HigA1 in BACTH assays (data not shown).

**Fig 8:**
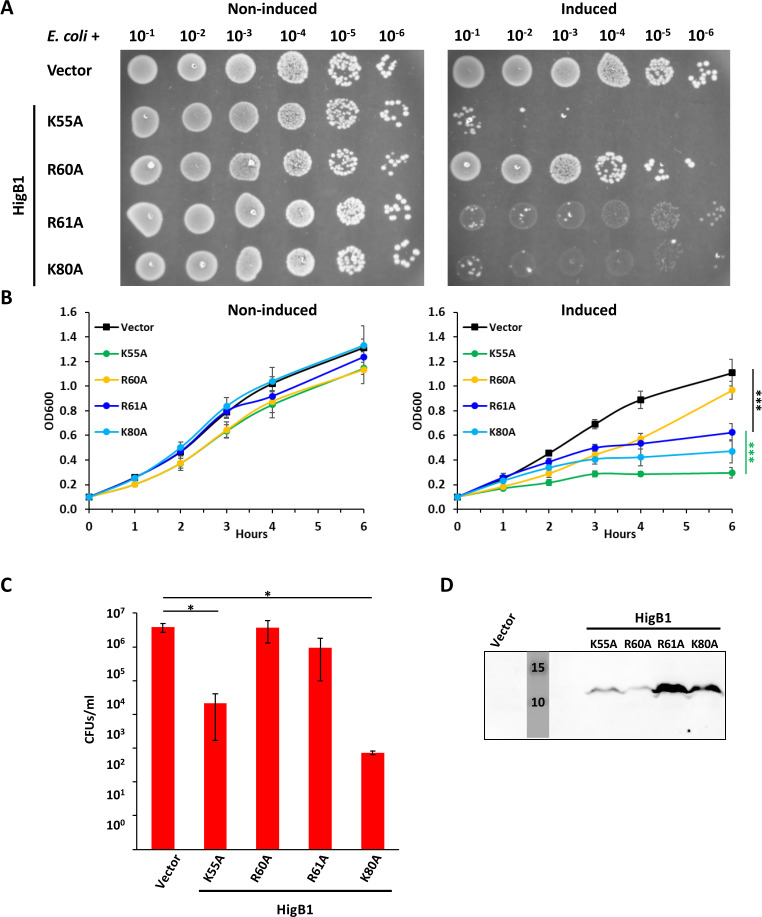
Toxicity of HigB1 is affected by mutation of conserved residues. Different alleles of *Waddlia* HigB1 were expressed in *E. coli* under control of P_ara_ on plasmid. (**A**) Efficiency of plating assay showing 10-fold serial dilutions of the different *E. coli* strains on Luria-Bertani medium with and without inducer. (**B**) Growth of the same strains in liquid medium, with (right) and without (left) inducer. In the presence of arabinose, cells expressing *higB1* R61A grew significantly less than cells carrying the empty vector and more than cells expressing *higB1* K55A. (**C**) Colony-forming units of *E. coli* carrying the empty vector or HigB1 variants after 6-h growth in liquid medium with inducer. (**D**) Immunoblot on *E. coli* expressing HigB1 variants from P_ara_*.* Samples were taken after 6 h of induction and normalized according to the OD_600 nm_. Molecular size standards are indicated in kilodalton. In panels B and C, values represent the means and SDs of three independent experiments. Statistical significance was determined by one-way ANOVA on area under the curve (**B**) or CFU values (**C**). *P < 0.05, ***P < 0.001.

*Waddlia* HigB1 and HigB2 are predicted to have a folding similar to HigB from *S. pneumoniae* (Table S1), whose active site residues have been described (62). Based on this structural similarity, we sought to identify residues important for the activity of *Waddlia* HigB toxins. We generated four point mutations in HigB1 (K55A, R60A, R61A, and K80A) and three point mutations in HigB2 (R60A, R68A, and K87A). In the case of HigB1, the variants were expressed either alone or in operon with HigA1, in order to compare them with the WT HigB1 that could be expressed in *E. coli* only from the complete *higBA1* operon (Fig. 8; Fig. S9). All the variants accumulated in *E. coli* at much higher levels than the WT HigB1, indicating at least a partial decrease in toxicity. In particular, the HigB1 R61A allele only slightly affected *E. coli* growth when expressed alone or together with HigA1, despite accumulation at high levels compared to the other alleles. The K80A mutation also partially decreased HigB1 toxicity, whereas the effect of the K55A mutation was mild, and the R60A mutation only slightly attenuated the phenotype compared to WT HigB1 (Fig. S9). However, all the variants could be expressed in *E. coli* also in the absence of HigA1, in contrast to WT HigB1 (Fig. 8). Moreover, as accumulation of the HigB1 R61A allele was tolerated in *E. coli*, it allowed the detection of a stronger interaction with HigA1 in a BACTH assay (Fig. 7E).

The ability of HigA1 to repress its own promoter, alone or in complex with HigB1, was assessed in *E. coli* using a promoter-reporter assay (P_higBA1_*-lacZ*), which confirmed negative auto-regulation of P_higBA1_ by HigA1 (Fig. 7D). Expression of the HigBA1 complex had no effect on the activity of the P_higBA1_ promoter, but the levels of HigA1 protein were also severely reduced in the presence of HigB1 (Fig. S9D). We therefore confirmed the reduced ability of the HigBA1 complex to repress P_higBA1_ by expressing the HigB1(R61A)-HigA1 and HigB1(K80A)-HigA1 complexes, which accumulate HigA1 at levels comparable to the strain expressing HigA1 alone (Fig. 7D; Fig. S9D).

**Fig 9:**
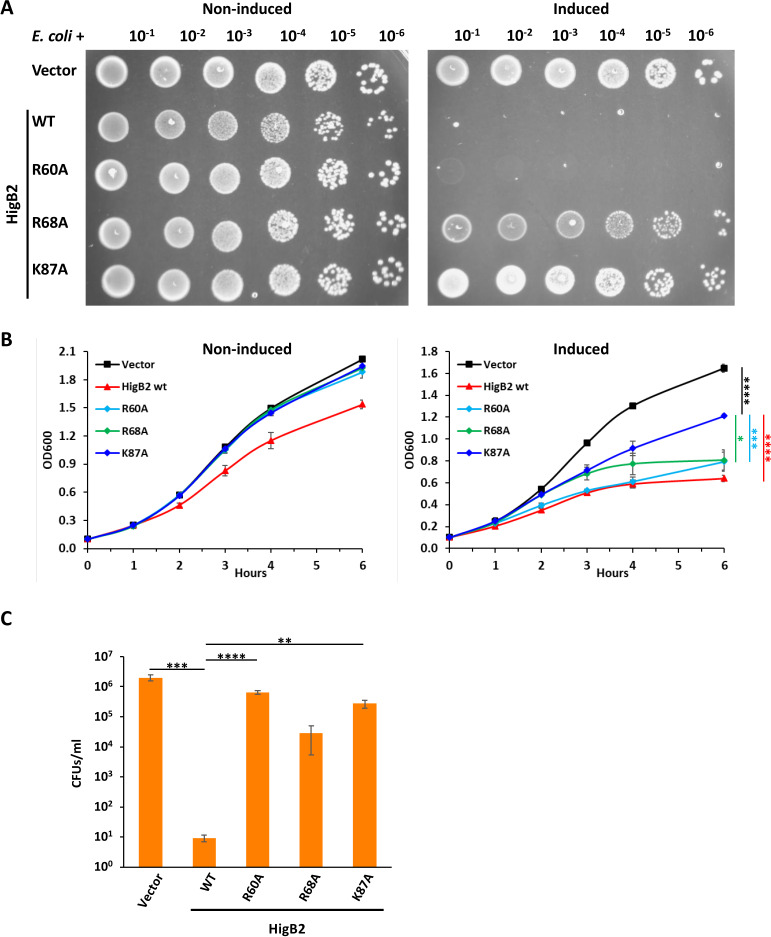
Toxicity of HigB2 is affected by mutation of conserved residues. Different alleles of *Waddlia* HigB2 were expressed in *E. coli* under control of P_a_*_ra_* on plasmid. (**A**) Efficiency of plating assay showing 10-fold serial dilutions of the different *E. coli* strains on Luria-Bertani medium with and without inducer. (**B**) Growth of the same strains in liquid medium, with (right) and without (left) inducer. In the presence of arabinose, cells expressing *higB2* K87A grew less than cells carrying the empty vector but significantly more than cells expressing *higB2* WT, R60A, or R68A. Without inducer, *E. coli* carrying the empty vector or expressing one of the *higB2* mutant alleles grow more than the strain expressing WT *higB2* (*P* < 0.0001). (**C**) Colony-forming units of *E. coli* carrying the empty vector or HigB2 variants after 6-h growth in liquid medium with inducer. In panels B and C, values represent the means and SDs of three independent experiments. Statistical significance was determined by one-way ANOVA on area under the curve (**B**) or CFU values (**C**). *P < 0.05, **P < 0.01, ***P < 0.001, ****P < 0.0001.

In the case of HigB2, the K87A variant restored *E. coli* growth compared to a strain expressing WT HigB2, whereas the R60A mutation had only a slight effect, and the R68A showed an intermediate phenotype (Fig. 9). As the phenotype observed for the heterologous expression of HigB2 in *E. coli* could also be due to different stability/accumulation of the alleles, we expressed the HigB2 allele also with an N-terminal V5-tag for immunodetection. Expression of the V5-tagged HigB2 seemed less deleterious for *E. coli* growth than the untagged version; however, all three point mutants showed decreased toxicity compared to the WT protein, despite the fact that they accumulated at much higher levels (Fig. S10). In this case, *E. coli* expressing the R68A or K87A HigB2 were indistinguishable from *E. coli* carrying the empty vector, whereas expression of the R60A allele determined an intermediate phenotype compared to the empty vector and WT HigB2.

#### Chromosome-encoded TA modules

**Fig 10:**
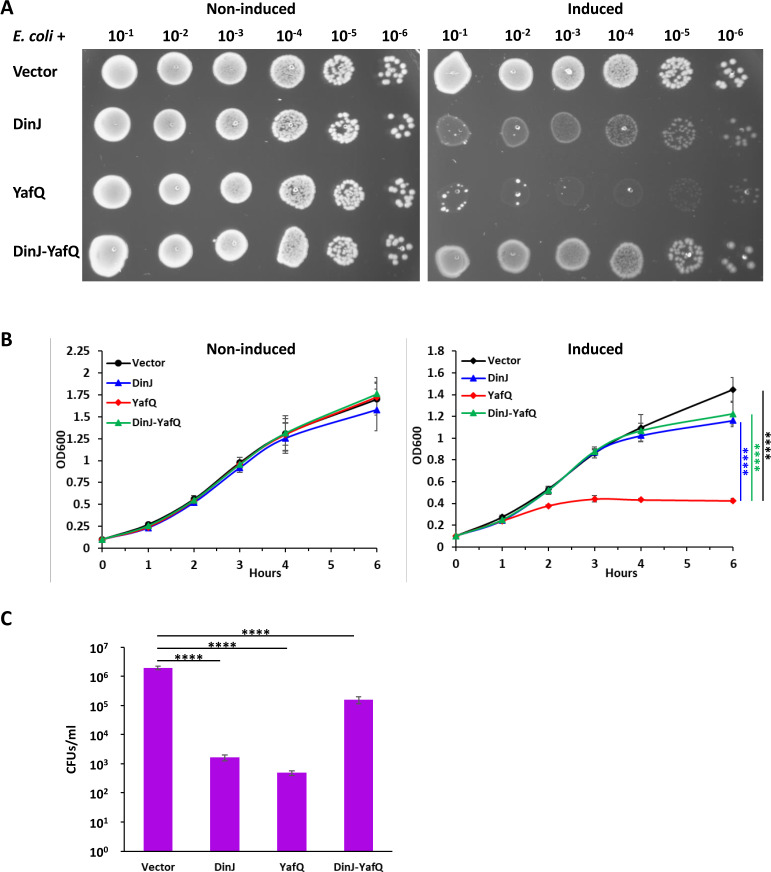
Expression of *Waddlia* YafQ in *E. coli* reduces growth. *Waddlia* DinJ and YafQ were expressed, alone or in combination, in *E. coli* under control of P_ara_ on plasmid. (**A**) Efficiency of plating assay showing 10-fold serial dilutions of the different *E. coli* strains on Luria-Bertani medium with and without inducer. (**B**) Growth of the same strains in liquid medium, with (right) and without (left) inducer. In the presence of arabinose, cells expressing *yafQ* grew significantly less than the other three strains. (**C**) Colony-forming units of the different *E. coli* strains after 6-h growth in liquid medium with inducer. In panels B and C, values represent the means and SDs of three independent experiments. Statistical significance was determined by one-way ANOVA on area under the curve (**B**) or CFU values (**C**). ****P < 0.0001.

The putative Doc and HicA toxins from *Waddlia* had no effect on *E. coli* growth (Fig. S11 and S12), although we cannot exclude that they were not correctly expressed/folded. We detected a decrease in *E. coli* growth and CFUs upon expression of the putative HicB antitoxin, which could be due to its DNA-binding activity or to interference with *E. coli* TA modules. Expression of the putative YafQ toxin was deleterious for *E. coli* growth both on plate and in liquid (Fig. 10) and significantly reduced CFUs. We observed a significant decrease in CFUs also upon expression of the putative antitoxin DinJ alone and, to a lesser extent, upon expression of the *Waddlia dinJ-yafQ* operon (Fig. 10).

## DISCUSSION

TA modules are considered rare in obligate intracellular bacteria and so far have never been investigated in members of the Chlamydiales. We identified seven putative type II TA modules in the *Chlamydia*-related species *Waddlia chondrophila* and assessed their expression profile in different conditions, as well as their functionality in a heterologous host. Among the seven putative type II TA pairs identified in the *Waddlia* genome, HigBA1 and MazEF1 are encoded on the pWc plasmid, whereas five other TA loci are found on the chromosome, including another HigBA and another MazEF module. The presence of type II TA modules is not unique to *Waddlia*, among *Chlamydia*-related species. A search of several available genomes with TAfinder identified putative type II TA loci on chromosomes and plasmids from different representative members belonging to distinct families in the Chlamydiales (Table 2). We expect that the continuously increasing number of available genomes in the Chlamydiales order will further expand the panel of TA modules present and their evolutionary history. TA-encoding genes could be remnants from the acquisition of mobile genetic elements. However, they are conserved in genomes that undergo a strong reductive selection pressure, such as those of obligate intracellular species, which suggests that these modules are still functional and play a role in the biology of these microorganisms. Conservation, and even duplication, of TA modules despite a strong genome reduction has been observed also in *Rickettsia* and *Wolbachia* species (32–34, 63).

**TABLE 2:** Type II TA modules identified in representatives of *Chlamydia*-related species^*a*^

Species	Family
Plasmid-encoded TA modules	
*Estrella lausannensis*	RelEB
*Rhabdochlamydia porcellionis*	VapBC
*Protochlamydia naegleriophila* KNic	HigBA (2×)
	RelEB (2×)
	Doc-Phd
	PIN-like domain
Chromosome-encoded TA modules	
*Rhabdochlamydia porcellionis*	1 TA module
*Rhabdochlamydia oedothoracis*	Doc-Phd (2×)
	2 TA modules
*Simkania negevensis*	RelEB
*Neochlamydia* sp. S13	PIN-like domain
	RelEB (7×)
*Parachlamydia acanthamoebae* UV7	Doc
	2 TA modules
*Protochlamydia naegleriophila* KNic	1 TA module

^*a*^ List of putative type II TA modules identified by TAfinder (https://bioinfo-mml.sjtu.edu.cn/TAfinder/TAfinder.php). The third column indicates if the prediction attributed the modules to a known TA family.

We showed that the two HigBA modules and the two MazEF modules from *Waddlia* are functional when expressed in *E. coli*, as the toxin is deleterious for *E. coli* growth, unless the cognate antitoxin is present. Moreover, point mutation of conserved residues shown to be important for the toxic activity of MazF and HigB from *E. coli*, *B. subtilis*, or *S. pneumoniae* (60–62) also impaired toxicity of *Waddlia* MazF and HigB.

Consistently with the fact that TA-encoding genes can be not only conserved but also even expanded in obligate intracellular bacteria (64), MazEF1 and MazEF2 share a high amino acid sequence identity and likely originate from a single acquisition event, with the duplication occurring then in *Waddlia* (Fig. S2). Gene duplication is crucial for bacterial adaptation (65) as it allows the duplicated genes to evolve toward new functions. It is therefore possible that the two MazEF modules play a role in distinct processes in *Waddlia*. As they also show distinct expression profiles, MazEF1 could be involved in plasmid maintenance and in response to some stressful conditions (such as iron starvation and DNA damage), whereas MazEF2 could be important for other conditions encountered *in vivo*, such as the adaptation to different hosts. Moreover, there could be cross-regulation between the two MazEF pairs, as in *E. coli* MazE2 can inhibit the toxicity of MazF1 and vice versa. Cross-regulation between homologous and also non-homologous TA modules has been shown in numerous cases, and it can happen both at the expression level and at the protein level [reviewed in reference (66)]. On the other hand, HigBA1 and HigBA2 were clearly acquired separately by *Waddlia*, and there is no cross-regulation, at least at the protein level. The *Waddlia* DinJ-YafQ pair is also functional in *E. coli*, whereas the other two modules encoded on the chromosome, Arc-Doc and HicAB, are not. Alignment of amino acid sequences from different Doc toxins with *Waddlia* Doc actually revealed that the motif described as essential for the activity of the Doc toxins [amino acid sequence HxFx(D/N)(A/G)NKR] (67, 68) is not conserved in the protein from *Waddlia*. On the other hand, Doc homologs encoded in several *Parachlamydia* and *Protochlamydia* spp. feature a conserved putative active site, suggesting that only *Waddlia* Doc lost its classical activity, the ability to phosphorylate the elongation factor EfTu on a conserved threonine residue (67, 69). Similarly, *Waddlia* HicA appear to be truncated compared to other HicA homologs, including a putative HicA from *Parachlamydia*. Taken together, these data suggest that in *Waddlia*, some chromosome-encoded TA modules are going toward the loss of their original function and could become pseudogenes (in a genome reduction process) or acquire new functions.

The function of TA modules is still somehow controversial, with three main biological activities supported by experimental data [reviewed in references (1, 11)]: post-segregational killing, defense against bacteriophages, and persistence. Being encoded on pWc, *Waddlia* HigBA1 and MazEF1 could therefore be involved in plasmid maintenance, with their mRNA accumulating in EBs in order to ensure that the TA pairs—and thus the plasmid—are maintained in the following infection cycle. Moreover, most of the chromosome-encoded TA modules we identified are close to transposases, which are relatively abundant in the *Waddlia* genome (37) and may represent marks of lateral gene transfer events. TA modules have been proposed to contribute to the maintenance of integrative mobile genetic elements as well (70, 71). Evidence is accumulating also for a role of TA systems in the defense against bacteriophages, either by abortive infection, a mechanism that impairs bacteriophage propagation through TA activation and altruistic suicide of infected cells before phage replication (72–74), or via disruption of phage replication by activated bacterial toxins [reviewed in reference (20)]. Although at the moment there are no bacteriophages known to infect *Waddlia*, it is possible that these bacteria, being able to infect a wide range of hosts including free-living amoebae, encounter other microorganisms in their natural environment and have evolved innate immunity systems, such as TA pairs, to fight against bacteriophages or other viruses/bacteria that compete for the same replicative niche. Finally, TA modules have often been associated with persistence, based on the hypothesis that upon exposure to stress, the labile antitoxin would be selectively degraded and the activity of the free toxin would determine the phenotypic transition of a subpopulation of bacteria into a state of dormancy that would allow survival to the adverse conditions (75, 76). This hypothesis was also supported by the transcriptional upregulation of numerous TA modules upon exposure to different abiotic stress stimuli (26, 77), consistent with what we also observed in ABs induced by iron starvation or novobiocin. However, this hypothesis has been questioned because it was shown that even a substantial increase in TA genes transcription did not result in significant levels of active toxin (17). Moreover, deletion of TA modules does not necessarily impact persister cell formation (18, 19, 78, 79). As no tools are available for genetic manipulation of *Waddlia*, we could not assess whether deletion of TA-encoding genes affects response to iron starvation or antibiotic treatment.

Nevertheless, our results show that the expression of TA modules is induced by iron starvation and DNA damage, whereas the β-lactams and glycopeptides tested (drugs that mainly affect the cell envelope) had a much more limited effect. The distinct expression profile of TA genes following exposure to different stress conditions corroborates previous results indicating that *Waddlia* ABs are morphologically and functionally different, depending on the trigger (53). They also suggest that Chlamydiaceae and *Chlamydia*-related species, despite sharing a conserved biphasic developmental cycle, evolved different strategies to adapt to their specific hosts and react to adverse conditions, consistently with different transcription profiles (52, 80). *Chlamydia*-related bacteria possess larger genomes compared to Chlamydiaceae, which provides them with enhanced metabolic capabilities and is often related to the broader host range of *Chlamydia*-related species (35, 37, 48). TA modules are part of this accessory genome, contributing to the adaptation and survival of *Chlamydia*-related species in hosts such as free-living amoebae, that they likely share with other microorganisms, being thus exposed to toxins and possibly bacteriophages. Further work will be required to elucidate the role(s) of TA modules in *Chlamydia*-related bacteria, and this would undoubtedly benefit from the development of tools for the genetic manipulation of these species.

## MATERIALS AND METHODS

### Cell culture and bacterial strains

HEp-2 (ATCC CCL-23) and Vero (ATCC CCL-81) cells were grown in high-glucose Dulbecco’s modified minimal essential medium (DMEM; PAN-Biotech, Aidenbach, Germany) supplemented with 10% fetal bovine serum (FBS; Gibco, Thermo Fisher Scientific, Waltham, MA, USA) at 37°C in the presence of 5% CO_2_. *Acanthamoeba castellanii* (ATCC 30010) was cultured in peptone/yeast extract/glucose (PYG) medium at 25°C. *Waddlia chondrophila* (strain ATCC VR-1470) was co-cultivated with *A. castellanii* in PYG medium at 32°C. *Escherichia coli* strains (Top10, except for BACTH assay that were carried out in a Δ*cyaA* strain) were grown at 37°C in Luria-Bertani (LB) medium, supplemented with antibiotics when appropriate (kanamycin, 50 µg/mL; spectinomycin, 90 µg/mL; tetracycline, 10 µg/mL; and ampicillin, 100 µg/mL). Antibiotics were purchased from AppliChem (Darmstadt, Germany).

### Antibodies against *Waddlia* HigB1 and MazF toxins

Polyclonal mouse antibodies against *Waddlia* HigB1 (antigen peptide DIKQASNILRKLVKS, corresponding to amino acids 86–100) and *Waddlia* MazF (antigen peptide DPDPVKGNEIGKKVR, corresponding to amino acids 17–31) were obtained from Eurogentec (Liège, Belgium).

### Infection procedure and aberrant body induction

HEp-2 and Vero cells were seeded at 4 × 10^6^ cells per 25-cm^2^ flask (for RNA extraction) or at 2.5 × 10^5^ cells per well in 24-well plates (for immunofluorescence) the day before infection. *Waddlia* suspension was prepared by filtering a 5-day-old co-culture through a 5-μM pore filter (Millipore, Carrigtwohill, Ireland) to eliminate amoebal cysts and trophozoites. Mammalian cells were infected with the bacterial filtrate diluted 100-fold (in case of subsequent treatment with drugs that induce aberrant body formation) or 1,000-fold (for kinetics of untreated infections). Infected cells were centrifuged at 1,790 × *g* for 10 min at room temperature and incubated at 37°C with 5% CO_2_ for 15 min. The medium containing non-internalized bacteria was then removed and replaced with fresh DMEM supplemented with 10% FBS.

In order to induce aberrant body formation, drugs were added at 2 hpi (teicoplanin, 250 µg/mL, and vancomycin, 500 µg/mL) or 8 hpi (BPDL, 75 µM; deferoxamine, 260 µg/mL; novobiocin, 250 µg/mL; penicillin G, 1,000 µg/mL; piperacillin, 500 µg/mL; mecillinam, 200 µg/mL; clavulanate, 900 µg/mL; and fosfomycin, 500 µg/mL). Clavulanic acid, deferoxamine, mecillinam, novobiocin, penicillin G, fosfomycin, piperacillin, teicoplanin, and 2,2′-bipyridyl were purchased from Sigma-Aldrich (Buchs, Switzerland). Vancomycin was purchased from AppliChem.

### RNA extraction and quantitative RT-PCR

*Waddlia*-infected cells were harvested in TRIzol (AmbionR, Life Technologies, Thermo Fisher Scientific, Waltham, MA, USA) at different times post infection, as previously described (56). RNA-extraction was performed according to the manufacturer’s instructions. RNA was re-suspended in 60 µL of water and treated with the DNA-free DNA removal kit (Invitrogen, Thermo Fisher Scientific) to remove DNA contaminations. Random primers and the GoScript Reverse Transcription kit (Promega, Duebendorf, Switzerland) were used to obtain cDNA. Gene expression was analyzed by quantitative PCR on 4 µL of fivefold diluted cDNA using iTaq SYBR Green (Bio-Rad, Cressier, Switzerland), and the primers listed in Table 3. Reactions were conducted on a QuantStudio3 system (Applied Biosystems, Thermo Fisher Scientific) using the following cycling conditions: 10 min at 95°C, 40 cycles of 15 s at 95°C, and 1 min at 60°C. Results were analyzed (with the ΔΔCt method) using 16S rRNA as endogenous control at 32 hpi (in the case of kinetic experiments) or the untreated sample (in the case of aberrant bodies) as reference sample. For fold changes ≤0.5 or ≥2, significance was assessed by paired *t*-test on ΔCt values.

**TABLE 3:** Oligonucleotides used in this study

Primer	Oligonucleotides used for real-time PCR (5′−3′)	Concentration (nM)
WadF4	GGC CCT TGG GTC GTA AAG TTC T	300
WadR4	CGGAGTTAGCCGGTGCTTCT	
q_p0002F1	AGA ATG GCT AGC AAC GCA AAC	200
q_p0002R1	GCT CCC ATA GAT CCG CAG AC	
q_p0003F1	GGA CTT CAC AAA GCC GAA CC	150
q_p0003R1	ACC TCC ATA GCT CCT TCG GG	
q_p0021F2	CAT CGA ACA TGC CTT GGA GAC T	350
q_p0021R2	CCA TCG GCT ATA GTT CCT TCT AA	
q_p0022F2	TAA TCC GCT TCA AGG TGA GG	300
q_p0022R2	GGA CAA TGA TAA TAA GGC CTG ACA	
q0004F1	TGC CAG GAA TAC GCG ACA AA	350
q0004R1	CCC TTT TCC TCC TGC ACA GT	
q1094F1	CAG CGT AGA AAT CCC TCT GGT	300
q1094R1	CGC TCT TTT CCC CGA TCC TT	
q1095F1	AGA TGT CGA AAA GCA GCT CA	350
q1095R1	ACA ACA GTT TTT GCT TCT CCG T	
q1195F1	ACA AAG TCC GTC TCA GCG TT	300
q1195R1	GTG GCA TCC TGT TTC TCG CT	
q1196F1	AGA AAT ACC GGC ACC AGC AC	300
q1196R1	AGC TTC CCT CAA GGT TGT GG	
q1207F1	GCT TTT TGA CCC CGA TCC G	200
q1207R1	CGC CTT CTG GTG GAT CAA TG	
q1208F1	ATC GAA CAT GCC TTG GAA GTG	350
q1208R1	ATC AGC TAT AGT CCC TTC CAG	
q1347F2	AAA TGT TGC TGA AGC GCA GG	300
q1347R2	GTG TGC CAT TTC GGA TTG CC	
q1348F1	AGA ACT CTA CGA AAC CGC CG	200
q1348R1	CAG GGT TTT GCA ATC GCC AA	
	Oligonucleotides used for plasmid construction (Sequence 5’−3’, restriction sites underlined)	
p0002F_Spe	aaa ACT AGT GAT GAA TCG ATA TAA GCT TTA TGA GAC CG	
p0002_stop_Kpn	aaa GGT ACC TCA ACT TTT GAC AAG TTT TCT AAG TAT G	
p0002R_Kpn	aaa GGT ACC GCA CTT TTG ACA AGT TTT CTA AGT ATG	
p0003F_Xba	aaa TCT AGA GAT GGG CAA AAT AAA GAC ATC AAG C	
p0003_stop_Kpn	aaa GGT ACC TTA GTG TGT AGA GGA ATA CAT AAT C	
p0003R_Kpn	aaa GGT ACC GCG TGT GTA GAG GAA TAC ATA ATC	
p0021F_Xba	aaa TCT AGA GAT GGC ACA AAC AGC AAG GAT CTC	
p0021_stop_Kpn	aaa GGT ACC TTA TTC TTC TTC AAA GCC ATC GG	
p0021R_Kpn	aaa GGT ACC GCT TCT TCT TCA AAG CCA TCG G	
p0022F_Xba	aaa TCT AGA GAT GGC TTT GAA GAA GAA TAA TCC GC	
p0022_stop_Kpn	aaa GGT ACC CTA AAT ATC AAT CCA CAG AAG ATC GTT C	
p0022R_Kpn	aaa GGT ACC GCA ATA TCA ATC CAC AGA AGA TCG TTC	
w1207F_Xba	aaa TCT AGA GAT GGC TTT GAA GAA GAA TCA TCC	
w1207_stop_Kpn	aaa GGT ACC TTA GAT GTC CAT CCA AAG AAG ATC G	
w1207R_Kpn	aaa GGT ACC GCG ATG TCC ATC CAA AGA AGA TCG	
w1208F_Xba	aaa TCT AGA GAT GGC GCA AAC AGC AAG AAT C	
w1208_stop_Kpn	aaa GGT ACC TCA CCC TGA AGA GGA TGA TTC	
w1208R_Kpn	aaa GGT ACC GCC CCT GAA GAG GAT GAT TC	
w1347F_Xba	aaa TCT AGA GAT GGC AAG AAG TAA AAA ATA TCA AG	
w1347_stop_Kpn	aaa GGT ACC TTA ACT TAA CCT AAG GTT CAA TCC C	
w1347R_Kpn	aaa GGT ACC GCA CTT AAC CTA AGG TTC AAT CCC	
w1348F_Xba	aaa TCT AGA GAT GGA TAA AAT CGA AAT AGA ACT C	
w1348_stop_Kpn	aaa GGT ACC TTA CTT CTT GCC ATG ACC TAT CTC	
w1348R_Kpn	aaa GGT ACC GCC TTC TTG CCA TGA CCT ATC TC	
p0002F_Nco	AAA CCA TGG CAA ATC GAT ATA AGC TTT ATG AGA CCG	
p0002R_Spe	AAA ACT AGT CAA CTT TTG ACA AGT TTT CTA AGT ATG	
p0003F_Nco	AAA CCA TGG GCA AAA TAA AGA CAT CAA GC	
p0003_V5_Sal	AAA GTC GAC TTA CGT AGA ATC GAG ACC GAG GAG AGG GTT AGG GAT AGG CTT ACC GTG TGT AGA GGA ATA CAT AAT C	
p0021F_Nco	AAA CCA TGG CAC AAA CAG CAA GGA TCT C	
p0021R_Xba	AAA TCT AGA GAT TAT TCT TCT TCA AAG CCA TCG GC	
p0022F_Nco	AAA CCA TGG CTT TGA AGA AGA ATA ATC CGC	
p0022R_Xba	AAA TCT AGA CTA AAT ATC AAT CCA CAG AAG ATC GTT C	
w0003F_Nco	AAA CCA TGG TTA TGC GCA AAG ACA CCG TC	
w0003R_Xba	AAA TCT AGA GTC ATC TAA CAG AAA GAT CTT TC	
w0004F_Nco	AAA CCA TGG TGA TGA CAA TAA AAT TTC TAA CAG TC	
w0004R_Xba	AAA TCT AGA GGG CTT ACC TAG AGG TTG GTT G	
w1094F_Nco	AAA CCA TGG CAA TGT TAA TAG ACG CAA AAA TCA TT	
w1094R_Xba	AAA TCT AGA GTC ACT CCA TTC TCA ATA CAA TG	
w1095F_Nco	AAA CCA TGG TGT ATA ATA AAT TCA TGA AGA AAA AAG	
w1095R_Xba	AAA TCT AGA TTA ATA TGC CTT TAT AGG AAC AAC AGT T	
w1195F_Nco	AAA CCA TGG TCA TGA ACC GCC ACA AAG TCC G	
w1195R_Xba	AAA TCT AGA CTT AAT CTT CAG CAT TCG GAT CG	
w1196F_Nco	AAA CCA TGG GAA TGC TGA AGA TTA AGA ACT CG	
w1196R_Xba	AAA TCT AGA TTC TAA AAT AGC TCT GAA TGG GA	
w1207F_Nco	AAA CCA TGG CTT TGA AGA AGA ATC ATC C	
w1207R_Xba	AAA TCT AGA GGG TGT CGT CAA TTA GAT GTC C	
w1208F_Nco	AAA CCA TGG CGC AAA CAG CAA GAA TC	
w1208R_Xba	AAA TCT AGA TTC ACC CTG AAG AGG ATG ATT C	
w1347F_Nco	AAA CCA TGG CAA GAA GTA AAA AAT ATC AAG	
w1347R_Xba	AAA TCT AGA TTA ACT TAA CCT AAG GTT CAA TCC C	
w1348F_Nco	AAA CCA TGG ATA AAA TCG AAA TAG AAC TC	
w1348F_Kpn	AAA GGT ACC ATG GAT AAA ATC GAA ATA GAA CTC	
w1348R_Xba	AAA TCT AGA TTA CTT CTT GCC ATG ACC TAT CTC	
PhigBA1_EcoRI	AAA GAA TTC CACTCCAGTGGCGAGATGATAA	
PhigBA1_XbaI	AAA TCT AGA GCTAGCCATTCTAAGTACTCGT	
p0002_K55A	GAT CTA TGG GAG CTG GCA TTC AAT GAT GGC AGG	
p0002_K55A_as	CCT GCC ATC ATT GAA TGC CAG CTC CCA TAG ATC	
p0002_R60A	AAA TTC AAT GAT GGC GCG CGG ATT TAT TAT GTG	
p0002_R60A_as	CAC ATA ATA AAT CCG CGC GCC ATC ATT GAA TTT	
p0002_R61A	TTC AAT GAT GGC AGG GCG ATT TAT TAT GTG CTA	
p0002_R61A_as	TAG CAC ATA ATA AAT CGC CCT GCC ATC ATT GAA	
p0002_K80A	TTA TTA GGA GGA AAT GCA AAT GGG CAA AAT AAA	
p0002_K80A_as	TTT ATT TTG CCC ATT TGC ATT TCC TCC TAA TAA	
w1348_R60A	GGA GTT TGC GAG CTT GCG ATT CAC TAC GGG CCC	
w1348_R60A_as	GGG CCC GTA GTG AAT CGC AAG CTC GCA AAC TCC	
w1348_R68A	TAC GGG CCC GGA ATT GCA ATT TAT TAC GGG AAG	
w1348_R68A_as	CTT CCC GTA ATA AAT TGC AAT TCC GGG CCC GTA	
w1348_K87A	TTT TGC GGT GGC GAC GCA GGC TCC CAA GAC AGG	
w1348_K87A_as	CCT GTC TTG GGA GCC TGC GTC GCC ACC GCA AAA	
p0022_R31A	ATT GGG AAG AAG GTT GCT CCT GCT CTT GTC GTC	
p0022_R31A_as	GAC GAC AAG AGC AGG AGC AAC CTT CTT CCC AAT	
p0022_T55A	ATC ATT GTC CCC ATC GCA AGC AAG GAC AAG AAA AT	
p0022_T55A_as	ATT TTC TTG TCC TTG CTT GCG ATG GGG ACA ATG AT	
p0022_H64A	GAC AAG AAA ATT CCT TCC GCT ATT CGG ATT GAG CCA	
p0022_H64A_as	TGG CTC AAT CCG AAT AGC GGA AGG AAT TTT CTT GTC	
w1207_R31A	ATC GGG AAG AAG GTT GCT CCT GCT CTT GTT GTC	
w1207_R31A_as	GAC AAC AAG AGC AGG AGC AAC CTT CTT CCC GAT	
w1207_T55A	ATT GTT GTC CCT ATC GCA AGC AAA GAC AAA AAA AT	
w1207_T55A_as	ATT TTT TTG TCT TTG CTT GCG ATA GGG ACA ACA AT	
w1207_H64A	GAC AAA AAA ATT CCC TCG GCT ATT CGC ATT GAT CCA	
w1207_H64A_as	TGG ATC AAT GCG AAT AGC CGA GGG AAT TTT TTT GTC	
mazF_E83A	AGT TTT GCT GTA TGC GCG CAA GTT CGG TCT ATT	
mazF_E83A_as	AAT AGA CCG AAC TTG CGC GCA TAC AGC AAA ACT	
mazF_Q84A	TTT GCT GTA TGC GAG GCA GTT CGG TCT ATT AGC	
mazF_Q84A_as	GCT AAT AGA CCG AAC TGC CTC GCA TAC AGC AAA	
BAD_fwd	CTA CCT GAC GCT TTT TAT CGC AA	
BAD_rev	GCG TTC TGA TTT AAT CTG TAT CAG G	

### Immunofluorescence

For immunofluorescence upon iron starvation, infected cells grown on glass coverslips were treated with BPDL 75 (μM) at 8 hpi and fixed with 4% paraformaldehyde (at room temperature for 15 min) 24 hpi, washed three times with PBS and incubated in blocking solution (10% fetal calf serum [FCS], 0.1% saponin, and 0.04% sodium azide in PBS) at 4°C for at least 2 h. For immunofluorescence of untreated, novobiocin-, and fosfomycin-treated samples, after the fixation step, coverslips were incubated 5 min at room temperature in PBS containing 0.5% Triton X-100 and then for 30 min at room temperature with Image-iT FX signal enhancer (Invitrogen, Thermo Fisher Scientific), before a blocking step (in PBS containing 10% FCS, 0.3% Triton and 0.2% sodium azide) of 15 min at 37°C.

For immunofluorescence staining, coverslips were incubated at room temperature with a polyclonal mouse antibody (diluted 1/100) obtained against a WcHigB1 peptide and a rabbit anti-*Waddlia* antibody (diluted 1/2,000) (81) in blocking solution for 2 h in a humidified chamber. As negative control for the anti-HigB1 antibody, a pre-immune serum was used at the same dilution as the final bleed. After three washing steps in PBS containing 0.1% saponin, coverslips were incubated at room temperature for 1 h with 15-ng/mL (150 ng/mL for BPDL-treated samples) 4′,6-diamidino-2-phenylindole (Molecular Probes, Thermo Fisher Scientific), a 1/1,000 dilution of Alexa Fluor 488-conjugated goat anti-mouse (Life technologies, Thermo Fisher Scientific), and a 1/1,000 dilution of Alexa Fluor 594-conjugated goat anti-rabbit (Life Technologies, Thermo Fisher Scientific). After washing twice with PBS 0.1% saponin, once with PBS, and once with deionized water, the coverslips were mounted onto glass slides with Mowiol (Sigma-Aldrich). Confocal images were obtained using a Zeiss LSM 900 microscope (Zeiss, Feldbach, Switzerland).

### Bacterial adenylate cyclase two-hybrid (BACTH) assays

Interactions between toxins and antitoxins were investigated using the BACTH system (Euromedex, France). The assays were performed according to the manufacturer instructions. Briefly, the genes of interest (*higA1*, *higB1* WT, R61A, *higA2*, *higB2* WT and K87A, *mazE1*, *mazF1*, *mazE2*, and *mazF2*) were cloned into plasmid, allowing fusion with the T25 or T18 fragment of the adenylate cyclase, in N-terminal (pKNT25/pUT18) or C-terminal (pKT25/pUT18C) position. Different combinations of T18 and T25 fusion plasmids were co-transformed in *E. coli*Δ*cyaA* strain. The β-galactosidase activity was measured after an overnight culture of three independent co-transformants, in LB medium supplemented with ampicillin, 100 µg/mL; kanamycin, 25 µg/mL; and isopropyl β-D-1-thiogalactopyranoside, 0.5 mM. Co-transformation with empty pK(N)T25 and pUT18(C) plasmids served as negative control for basal β-galactosidase activity. The β-galactosidase activity obtained with the proteins of interest was compared to the negative control by using *t*-test (Table S2).

### Immunoblots

*E. coli* samples from cultures expressing *Waddlia* TA modules were collected by centrifugation, and pellets were re-suspended in SDS-PAGE loading buffer. Samples were normalized for equivalent loading using OD_600 nm_ and heated at 95°C for 5 min before loading on SDS-PAGE and transfer onto nitrocellulose membrane (Amersham, Cytiva, Marlborough, MA, USA). Membranes were blocked for 3 h in Tris-buffered saline (10-mM Tris-HCl, 150-mM NaCl) containing 0.05% Tween-20 and 5% dry milk. Proteins of interest were detected using polyclonal mouse antibodies against *Waddlia* HigB1 or MazF (1/500 dilution), or a mouse monoclonal anti-V5 epitope antibody (Invitrogen, Thermo Fisher Scientific) (1/5,000 dilution) and incubated at 4°C overnight. Horseradish peroxidase-conjugated secondary antibodies, goat anti-mouse IgG (Bio-Rad), and donkey anti-rabbit IgG (Promega) were applied for 1 h at room temperature. Immunoblots were revealed with ECL Prime Western Blotting Detection Reagent (Amersham, Cytiva) using an ImageQuant LAS4000 mini system (Amersham, Cytiva).

### Protein sequence analysis

Phylogenies for the ortholog groups were generated on the Chlamydiae Database website (https://chlamdb.ch/) (57).

Secondary structure predictions were generated using the Phyre2 web portal for protein modeling (82).

### Heterologous expression in *E. coli*

Plasmids were constructed as described in the supplemental information. To assess the effect of *Waddlia* TA modules on *E. coli* (Top10) growth in liquid medium, overnight cultures were diluted to an initial OD_600 nm_ of 0.1 in a medium containing appropriate antibiotics, with or without arabinose 0.3%. Growth was monitored by measuring OD_600 nm_ at 1, 2, 3, 4, and 6 h. Area under the curve analysis followed by one-way analysis of variance (ANOVA) test was used to compare the growth of strains expressing different TA genes.

Survival of *E. coli* cells after 6-h induction (in liquid medium with arabinose 0.3%) of *Waddlia* TA modules was assessed by counting CFUs. Cultures were normalized according to the OD_600 nm_, and serial dilutions were plated on LB agar (supplemented with antibiotics but without arabinose) and incubated overnight. Colonies were counted about 20 h after plating. Statistical analysis of CFU values was performed by using one-way ANOVA test.

The effect of *Waddlia* TA modules on *E. coli* (Top10) growth on solid medium was assessed by the efficiency of plating assays. Overnight cultures were diluted in LB medium to an OD_600 nm_ = 0.5, and 10-fold serial dilutions were thus plated on LB agar supplemented with appropriate antibiotics, with and without arabinose 0.3% (5 μL per spot). Photos were taken 20–24 h after plating.

### β-galactosidase activity assays

The ability of HigA1 to repress its own promoter (P_higBA1_) in the heterologous host, *E. coli*, was assessed by β-galactosidase assays. Overnight cultures were diluted in LB medium supplemented with antibiotics and arabinose 0.3% (to induce expression of *higA1* or *higBA1* from P_ara_) at an initial OD660_nm_ of 0.05 and grown for 3 h (final OD660_nm_ = 0.2–0.5). β-Galactosidase assays were performed at 30°C on 50 µL of cells. Cells were lysed with 30 µL of chloroform and mixed with Z buffer (60-mM Na_2_HPO_4_, 40-mM NaH_2_PO_4_, 10-mM KCl, 1-mM MgSO_4_, dithiothreitol 1 mM, SDS 0.0037% pH 7) to a final volume of 800 µL. Reaction was started and timed following the addition of 200 µL ofo-nitrophenyl-β-D-galactopyranoside of 4 mg/mL in 0.1-M potassium phosphate (pH 7). Upon medium-yellow color development, the reaction was stopped by adding 400 µL of Na_2_CO_3_. OD_420 nm_ of the supernatant was recorded, and Miller units were calculated as follows: *U* = (OD_420 nm_ × 1,000) / (OD_660 nm_ × *t* (min) × vol (mL)). Error was computed as standard deviation. Data are from three biological replicates.

Oligonucleotides and plasmids used are listed in Tables 3 and 4, respectively.

**TABLE 4:** Plasmids used in this study

Plasmid	Relevant characteristics	Reference or source
pKT25	Low-copy plasmid for C-ter fusions to the T25 adenylate cyclase fragment, Kan^R^	Euromedex
pKNT25	Low-copy plasmid for N-ter fusions to the T25 adenylate cyclase fragment, Kan^R^	Euromedex
pUT18	High-copy plasmid for N-ter fusions to the T18 adenylate cyclase fragment, Amp^R^	Euromedex
pUT18C	High-copy plasmid for C-ter fusions to the T18 adenylate cyclase fragment, Amp^R^	Euromedex
pBAD22	Low-copy plasmid with arabinose-inducible promoter, Kana^R^	(83)
pBAD101	Low-copy plasmid with arabinose-inducible promoter, Strep^R^ Spec^R^	(84)
pRKlac290	*lacZ* transcriptional fusion vector, Tet^R^	(85)
pSA202	pKT25-*mazF2*, Kan^R^	This work
pSA203	pKT25-*mazE2*, Kan^R^	This work
pSA204	pKT25-*mazE1*, Kan^R^	This work
pSA205	pKT25-*mazF1*, Kan^R^	This work
pSA206	pKNT25-*mazF2*, Kan^R^	This work
pSA207	pKNT25-*mazE2*, Kan^R^	This work
pSA208	pKNT25-*mazE1*, Kan^R^	This work
pSA209	pKNT25-*mazF1*, Kan^R^	This work
pSA210	pUT18-*mazF2*, Amp^R^	This work
pSA211	pUT18-*mazE2*, Amp^R^	This work
pSA212	pUT18-*mazE1*, Amp^R^	This work
pSA213	pUT18-*mazF1*, Amp^R^	This work
pSA214	pUT18C-*mazF2*, Amp^R^	This work
pSA215	pUT18C-*mazE2*, Amp^R^	This work
pSA216	pUT18C-*mazE1*, Amp^R^	This work
pSA217	pUT18C-*mazF1*, Amp^R^	This work
pSA224	pKT25-*higB1*, Kan^R^	This work
pSA225	pKT25-*higA1*, Kan^R^	This work
pSA227	pKT25-*higA2*, Kan^R^	This work
pSA228	pKT25-*higB2*, Kan^R^	This work
pSA229	pKNT25-*higB1*, Kan^R^	This work
pSA230	pKNT25-*higA1*, Kan^R^	This work
pSA232	pKT25-*higA2*, Kan^R^	This work
pSA233	pKT25-*higB2*, Kan^R^	This work
pSA234	pUT18-*higB1*, Amp^R^	This work
pSA235	pUT18-*higA1*, Amp^R^	This work
pSA237	pUT18-*higA2*, Amp^R^	This work
pSA238	pUT18-*higB2*, Amp^R^	This work
pSA239	pUT18C-*higB1*, Amp^R^	This work
pSA240	pUT18C-*higA1*, Amp^R^	This work
pSA242	pUT18C-*higA2*, Amp^R^	This work
pSA243	pUT18C-*higB2*, Amp^R^	This work
pSA316	pKT25-*higB2*(K87A), Kan^R^	This work
pSA319	pKNT25-*higB2*(K87A), Kan^R^	This work
pSA322	pUT18-*higB2*(K87A), Amp^R^	This work
pSA325	pUT18C-*higB2*(K87A), Amp^R^	This work
pSA326	pKT25-*higB1*(R61A), Kan^R^	This work
pSA327	pKNT25-*higB1*(R61A), Kan^R^	This work
pSA328	pUT18-*higB1*(R61A), Amp^R^	This work
pSA329	pUT18C-*higB1*(R61A), Amp^R^	This work
pSA153	pBAD101 derivative carrying *Wc_arc* ORF, Spec^R^	This work
pSA154	pBAD101 derivative carrying *Wc_doc* ORF, Spec^R^	This work
pSA155	pBAD101 derivative carrying *Wc_arc-doc* operon, Spec^R^	This work
pSA156	pBAD101 derivative carrying *Wc_hicB* ORF, Spec^R^	This work
pSA157	pBAD101 derivative carrying *Wc_hicA* ORF, Spec^R^	This work
pSA158	pBAD101 derivative carrying *Wc_hicAB* operon, Spec^R^	This work
pSA159	pBAD101 derivative carrying *Wc_dinJ* ORF, Spec^R^	This work
pSA160	pBAD101 derivative carrying *Wc_yafQ* ORF, Spec^R^	This work
pSA161	pBAD101 derivative carrying *Wc_dinJ-yafQ* operon, Spec^R^	This work
pSA62	pBAD101 derivative carrying *Wc_higA1* ORF, Spec^R^	This work
pSA101	pBAD101 derivative carrying *Wc_higB1* ORF, Spec^R^	This work
pSA334	pBAD101 derivative carrying *Wc_higBA1* (V5-tagged HigA1) operon, Spec^R^	This work
pSA279	pBAD101 derivative carrying *Wc_higA2* ORF, Spec^R^	This work
pSA280	pBAD101 derivative carrying *Wc_higB2* ORF, Spec^R^	This work
pSA281	pBAD101 derivative carrying *Wc_higBA2* operon, Spec^R^	This work
pSA103	pBAD101 derivative carrying *Wc_mazE1* ORF, Spec^R^	This work
pSA193	pBAD22 derivative carrying *Wc_mazE1* ORF, Kana^R^	This work
pSA104	pBAD101 derivative carrying *Wc_mazF1* ORF, Spec^R^	This work
pSA105	pBAD101 derivative carrying *Wc_mazEF1* operon, Spec^R^	This work
pSA163	pBAD101 derivative carrying *Wc_mazE2* ORF, Spec^R^	This work
pSA197	pBAD22 derivative carrying *Wc_mazE2* ORF, Kana^R^	This work
pSA162	pBAD101 derivative carrying *Wc_mazF2* ORF, Spec^R^	This work
pSA164	pBAD101 derivative carrying *Wc_mazEF2* operon, Spec^R^	This work
pSA292	pBAD101 derivative carrying *Wc_higB1* (K55A), Spec^R^	This work
pSA293	pBAD101 derivative carrying *Wc_higB1* (R60A), Spec^R^	This work
pSA294	pBAD101 derivative carrying *Wc_higB1* (R61A), Spec^R^	This work
pSA295	pBAD101 derivative carrying *Wc_higB1* (K80A), Spec^R^	This work
pSA330	pBAD101 derivative carrying *Wc_higBA1* operon (with *higB1* K55A variant and HigA1_V5), Spec^R^	This work
pSA331	pBAD101 derivative carrying *Wc_higBA1* operon (with *higB1* R60A variant and HigA1_V5), Spec^R^	This work
pSA332	pBAD101 derivative carrying *Wc_higBA1* operon (with *higB1* R61A variant and HigA1_V5), Spec^R^	This work
pSA333	pBAD101 derivative carrying *Wc_higBA1* operon (with *higB1* K80A variant and HigA1_V5), Spec^R^	This work
pSA289	pBAD22 derivative carrying *Wc-higB2* (R60A), Kana^R^	This work
pSA290	pBAD22 derivative carrying *Wc-higB2* (R68A), Kana^R^	This work
pSA291	pBAD22 derivative carrying *Wc-higB2* (K87A), Kana^R^	This work
pSA89	pBAD101 derivative for V5 N-terminal tag, Spec^R^	This work
pSA296	pSA89 derivative carrying *Wc-higB2*, Spec^R^	This work
pSA297	pSA89 derivative carrying *Wc-higB2* (R60A), Spec^R^	This work
pSA298	pSA89 derivative carrying *Wc-higB2* (R68A), Spec^R^	This work
pSA299	pSA89 derivative carrying *Wc-higB2* (K87A), Spec^R^	This work
pSA256	pBAD101 derivative carrying *Wc_mazF1* (E83A), Spec^R^	This work
pSA257	pBAD101 derivative carrying *Wc_mazF1* (Q84A), Spec^R^	This work
pSA258	pBAD101 derivative carrying *Wc_mazF2* (E83A), Spec^R^	This work
pSA259	pBAD101 derivative carrying *Wc_mazF1* (Q84A), Spec^R^	This work
pSA264	pBAD101 derivative carrying *Wc_mazF1* (T55A), Spec^R^	This work
pSA265	pBAD101 derivative carrying *Wc_mazF1* (R31A), Spec^R^	This work
pSA266	pBAD101 derivative carrying *Wc_mazF1* (H64A), Spec^R^	This work
pSA267	pBAD101 derivative carrying *Wc_mazF2* (T55A), Spec^R^	This work
pSA268	pBAD101 derivative carrying *Wc_mazF2* (R31A), Spec^R^	This work
pSA269	pBAD101 derivative carrying *Wc_mazF2* (H64A), Spec^R^	This work
pSA67	pRKlac290 derivative carrying P*_higBA1_-lacZ*, Tet^R^	This work

## Data Availability

Unprocessed immunoblots, efficiency of plating assays, and microscopy images have been deposited at Mendeley Data and are available at https://data.mendeley.com/datasets/27khnmwmt3/1.

## SUPPLEMENTAL MATERIAL

Figures S1-S4 aem.00681-23-s0001.pdf Orthogroup phylogeny of *Waddlia* HigBA and MazEF proteins*.* RNA levels of TAs in aberrant bodies and during *Waddlia* infection cycle.

Figure S5 aem.00681-23-s0002.pdf Expression of *Waddlia* MazEF2 in *E. coli*.

Figure S6 aem.00681-23-s0003.pdf Interaction between MazE1/2 and MazF1/2.

Figure S7 aem.00681-23-s0004.pdf Toxicity of MazF1 point mutants in *E. coli*.

Figure S8 aem.00681-23-s0005.pdf Toxicity of MazF2 point mutants in *E. coli*.

Figure S9 aem.00681-23-s0006.pdf Toxicity of HigB1 point mutants in *E. coli*.

Figure S10 aem.00681-23-s0007.pdf Toxicity of V5-HigB2 point mutants in *E. coli*.

Figure S11 aem.00681-23-s0008.pdf Expression of Arc-Doc in *E. coli*.

Figure S12 aem.00681-23-s0009.pdf Expression of HicAB in *E. coli*.

Supplemental information aem.00681-23-s0010.docx Legends to supplemental figures, supplemental Tables 1 and 2, Supplemental methods (plasmid construction).

ASM does not own the copyrights to Supplemental Material that may be linked to, or accessed through, an article. The authors have granted ASM a non-exclusive, world-wide license to publish the Supplemental Material files. Please contact the corresponding author directly for reuse.
