# Supplementary figures and images for: The Chlamydia-related Waddlia chondrophila encodes functional type II toxin-antitoxin systems
Source: Appl Environ Microbiol. 2024 Jan 12;90(2):e00681-23. doi: 10.1128/aem.00681-23 (PMC10880633; doi:10.1128/aem.00681-23)

A

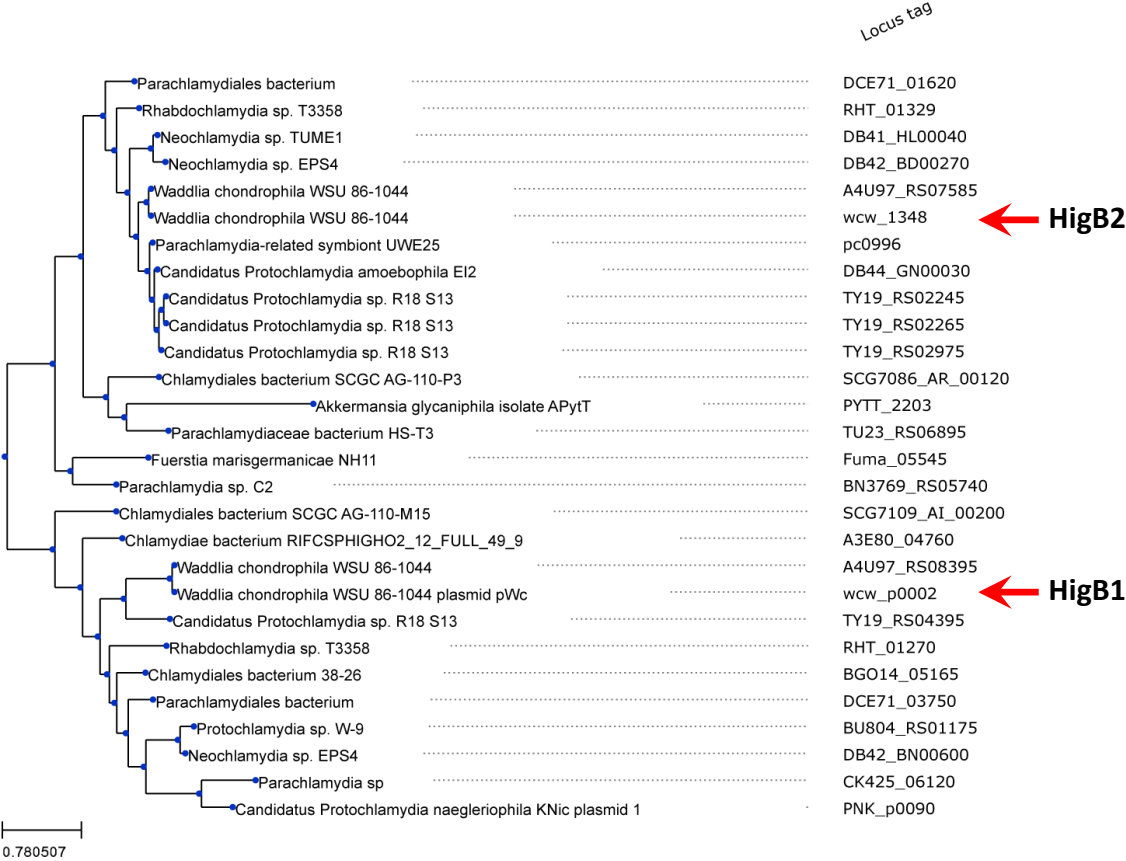

B

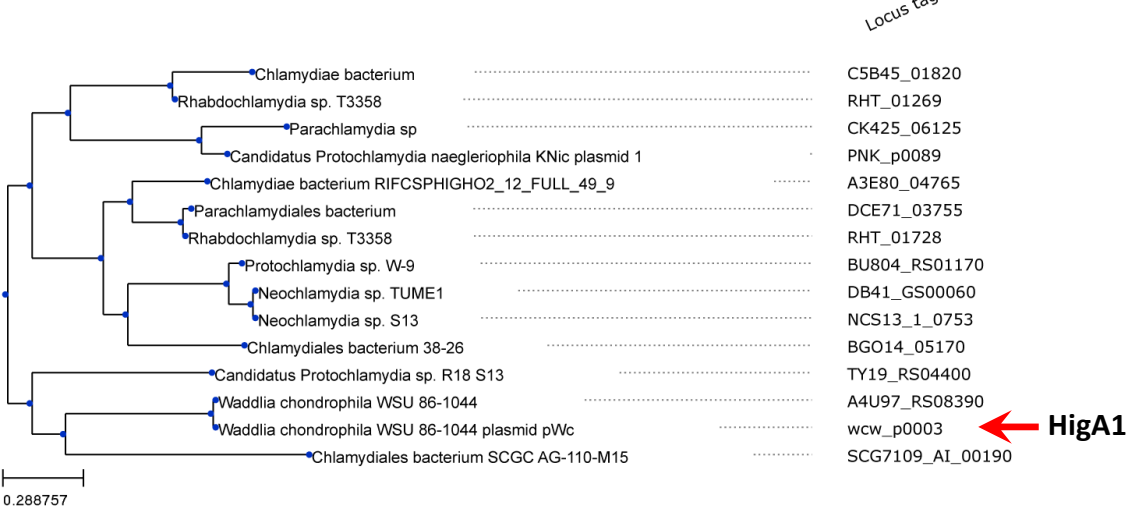

C

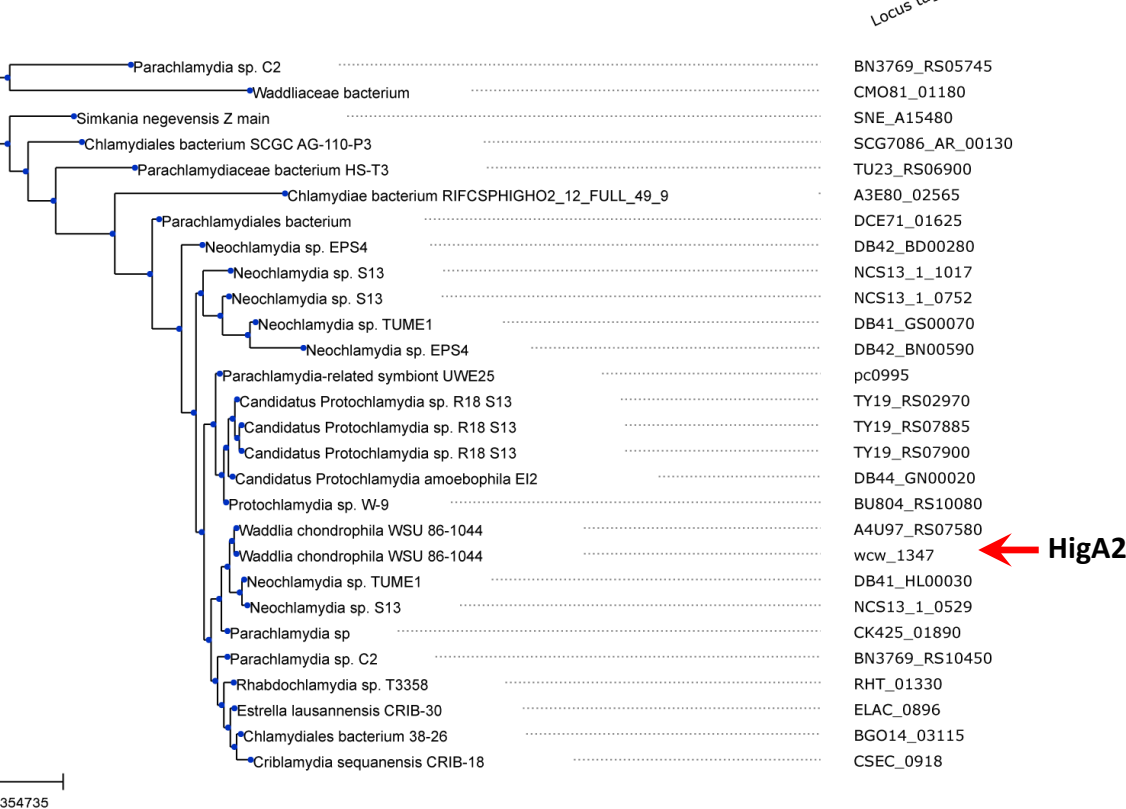

A

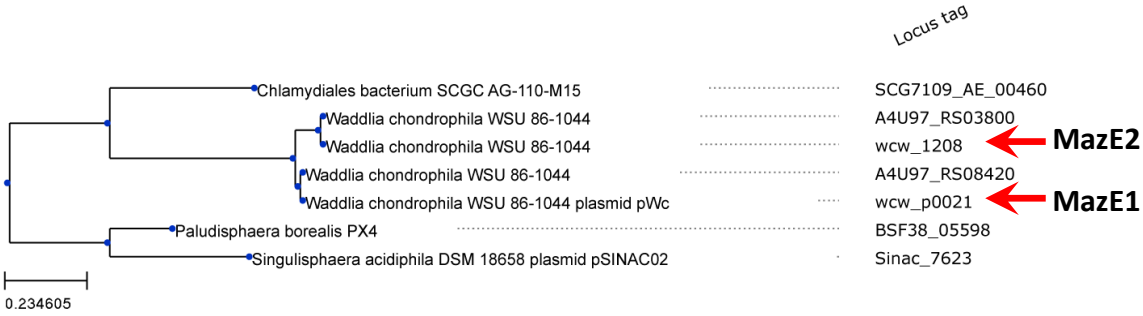

B

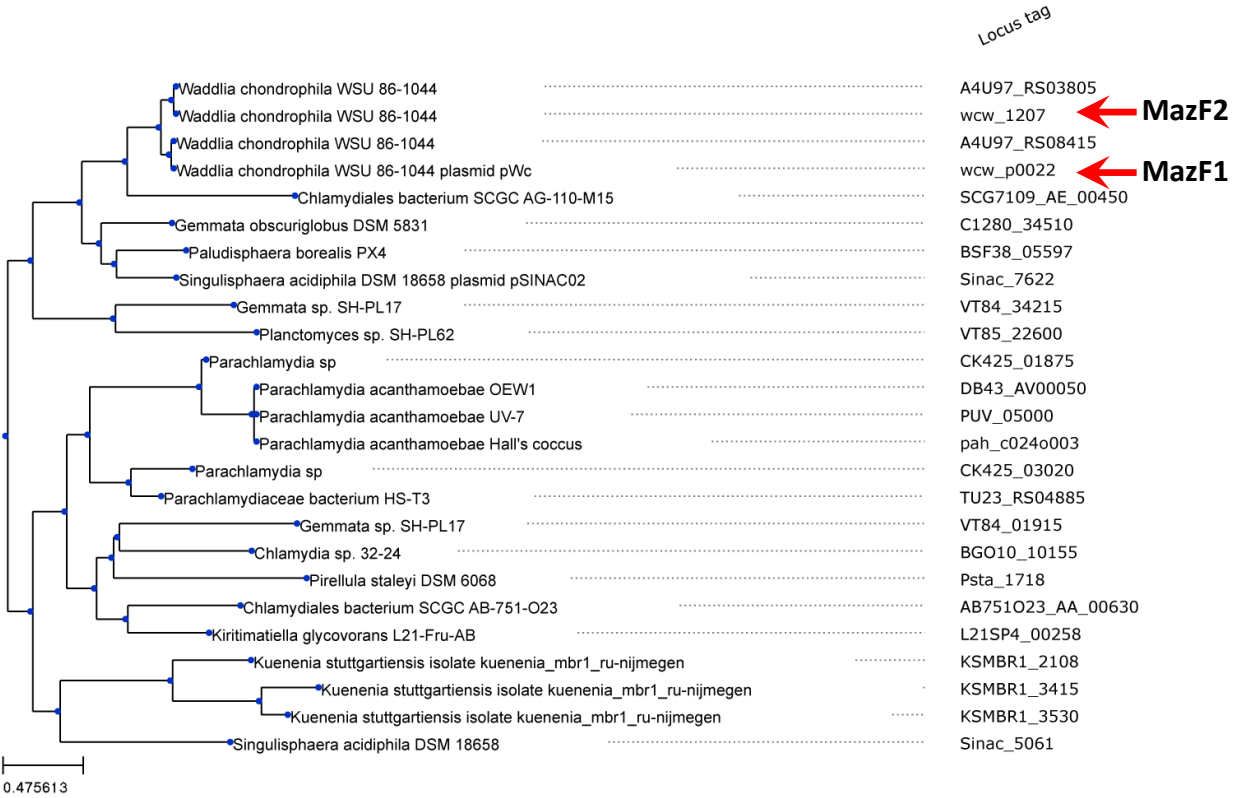

C

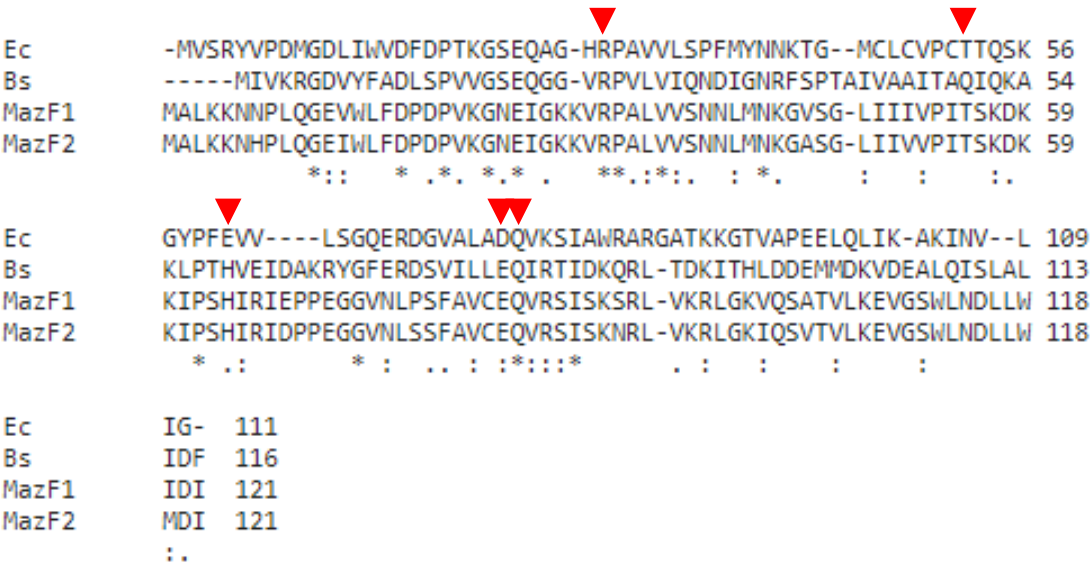

Fig S3

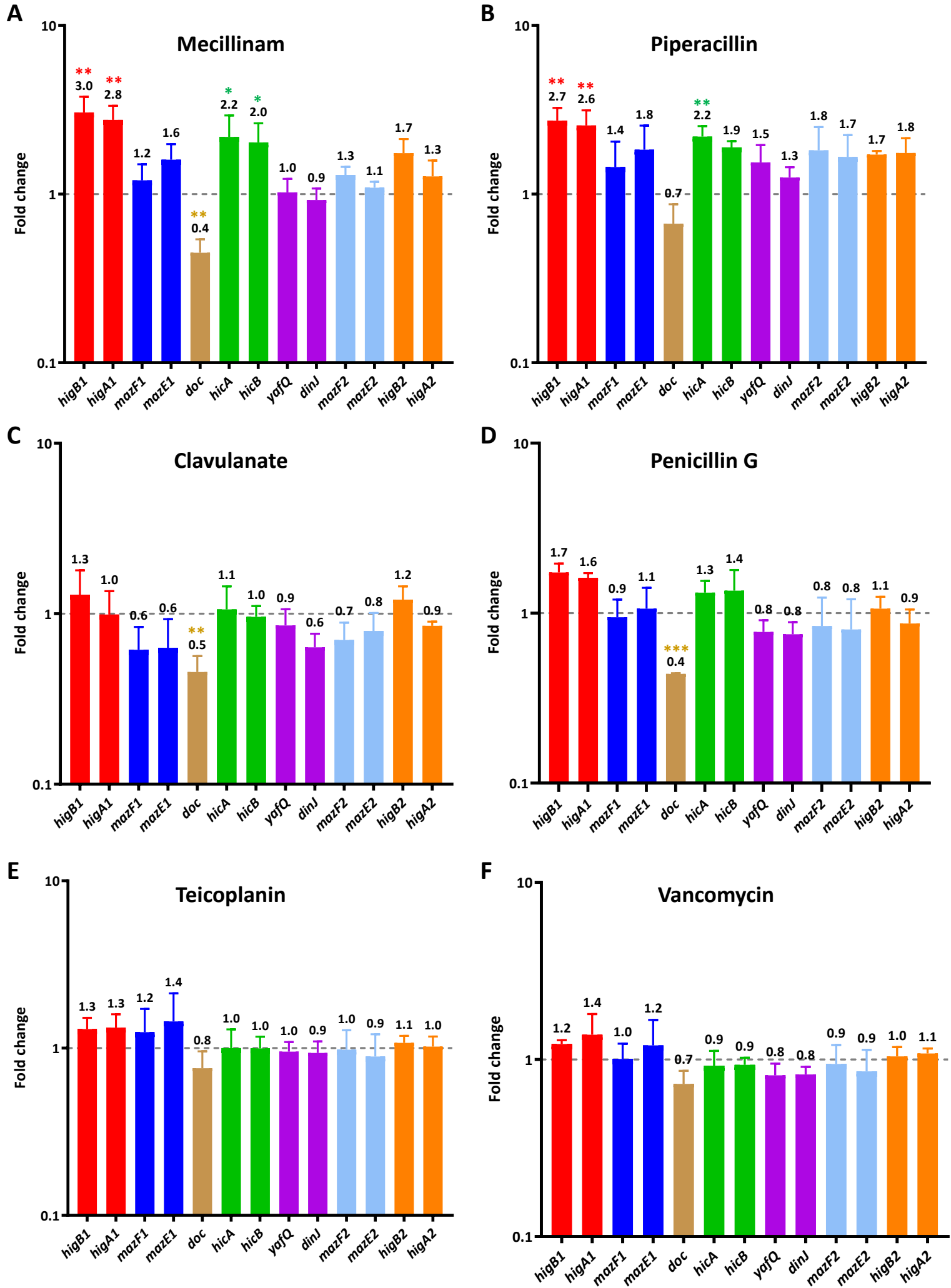

Fig S4

A

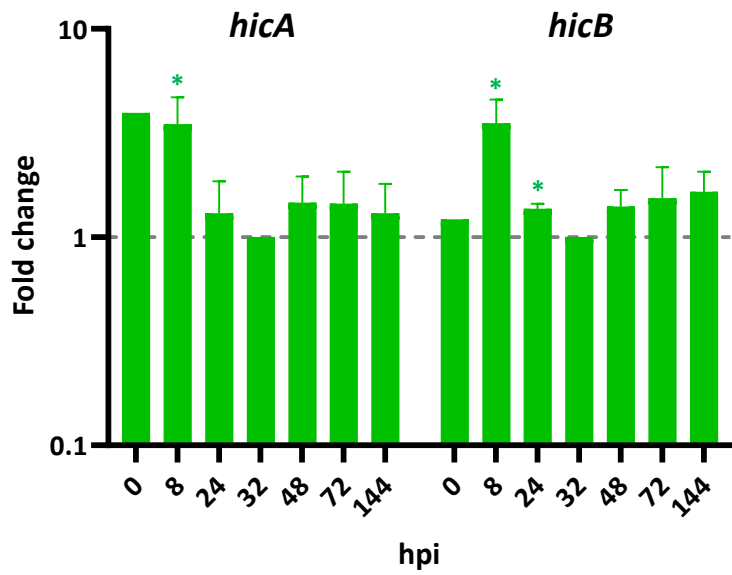

B

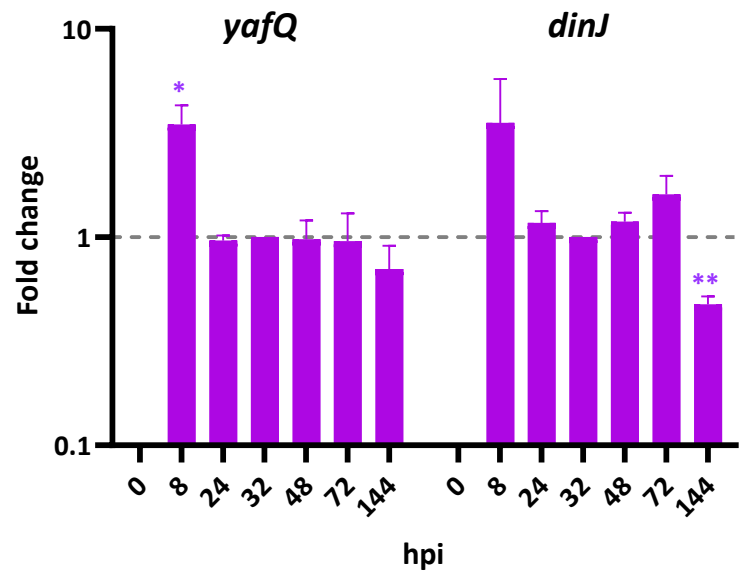

C

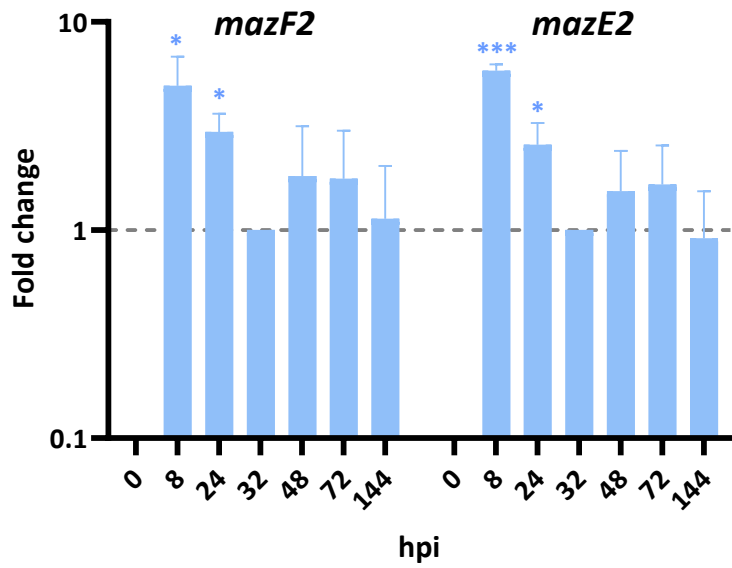

D

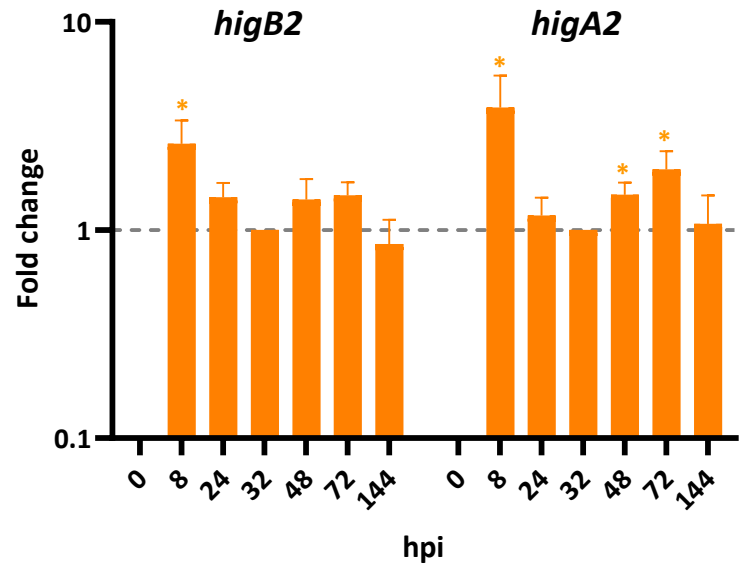

E

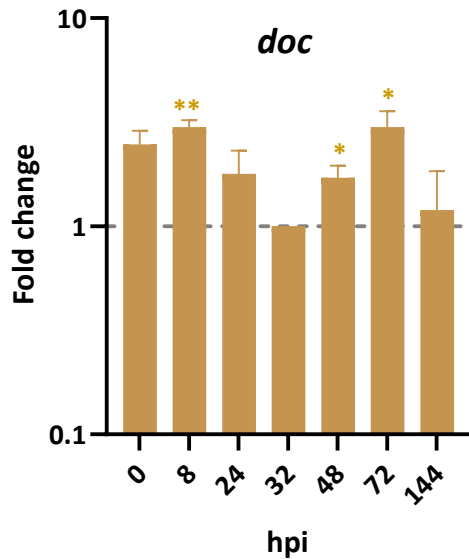

Supplement: Figures S1-S4 — Orthogroup phylogeny of Waddlia HigBA and MazEF proteins. RNA levels of TAs in aberrant bodies and during Waddlia infection cycle. [file aem.00681-23-s0001.pdf]

A

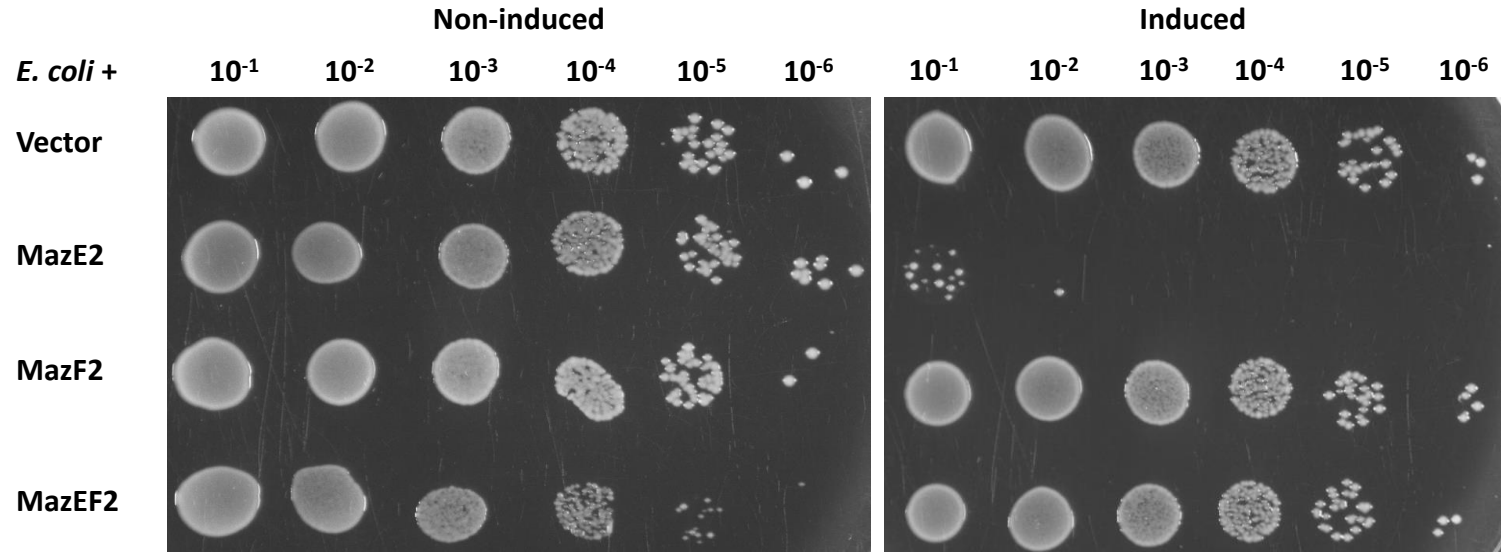

B

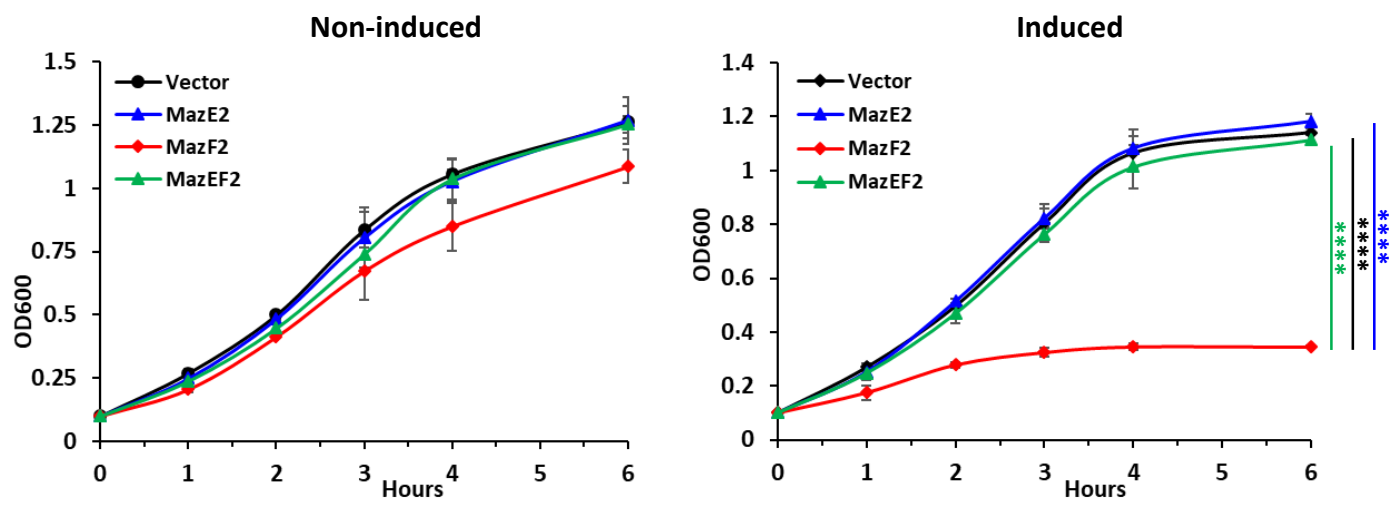

C

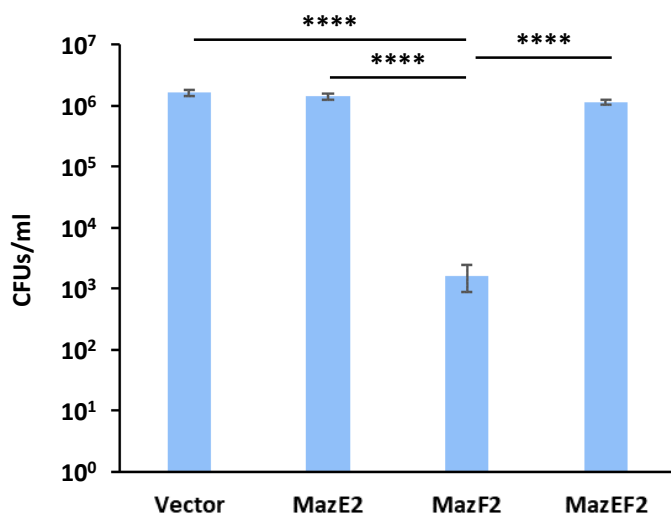

Supplement: Figure S5 — Expression of Waddlia MazEF2 in E. coli. [file aem.00681-23-s0002.pdf]

Fig S6

A

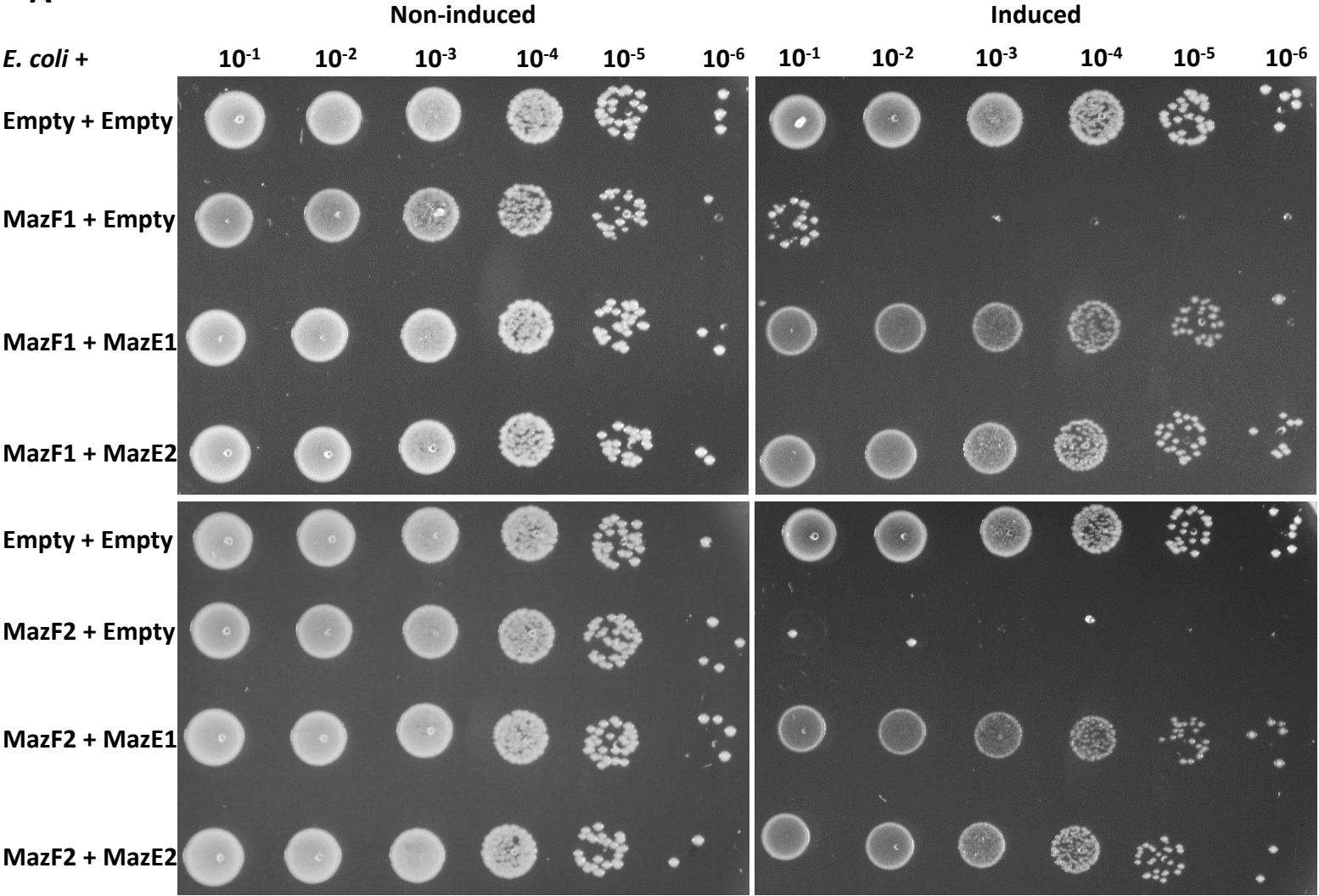

B

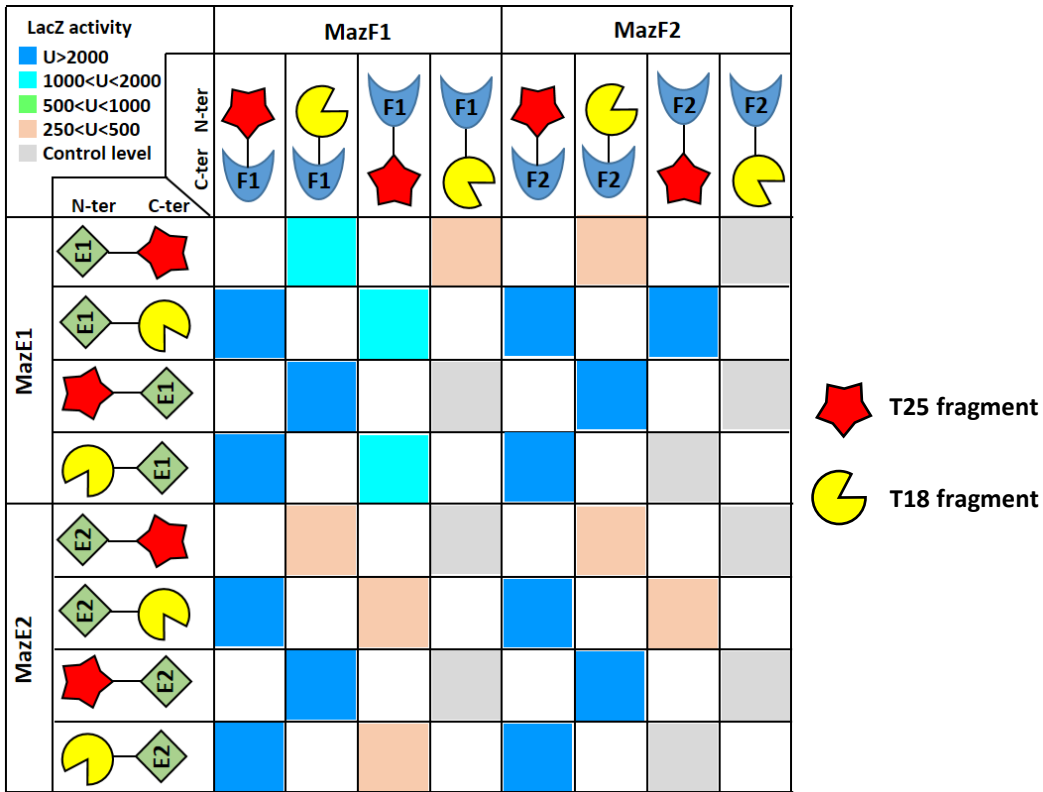

Supplement: Figure S6 — Interaction between MazE1/2 and MazF1/2. [file aem.00681-23-s0003.pdf]

**Fig S7****A**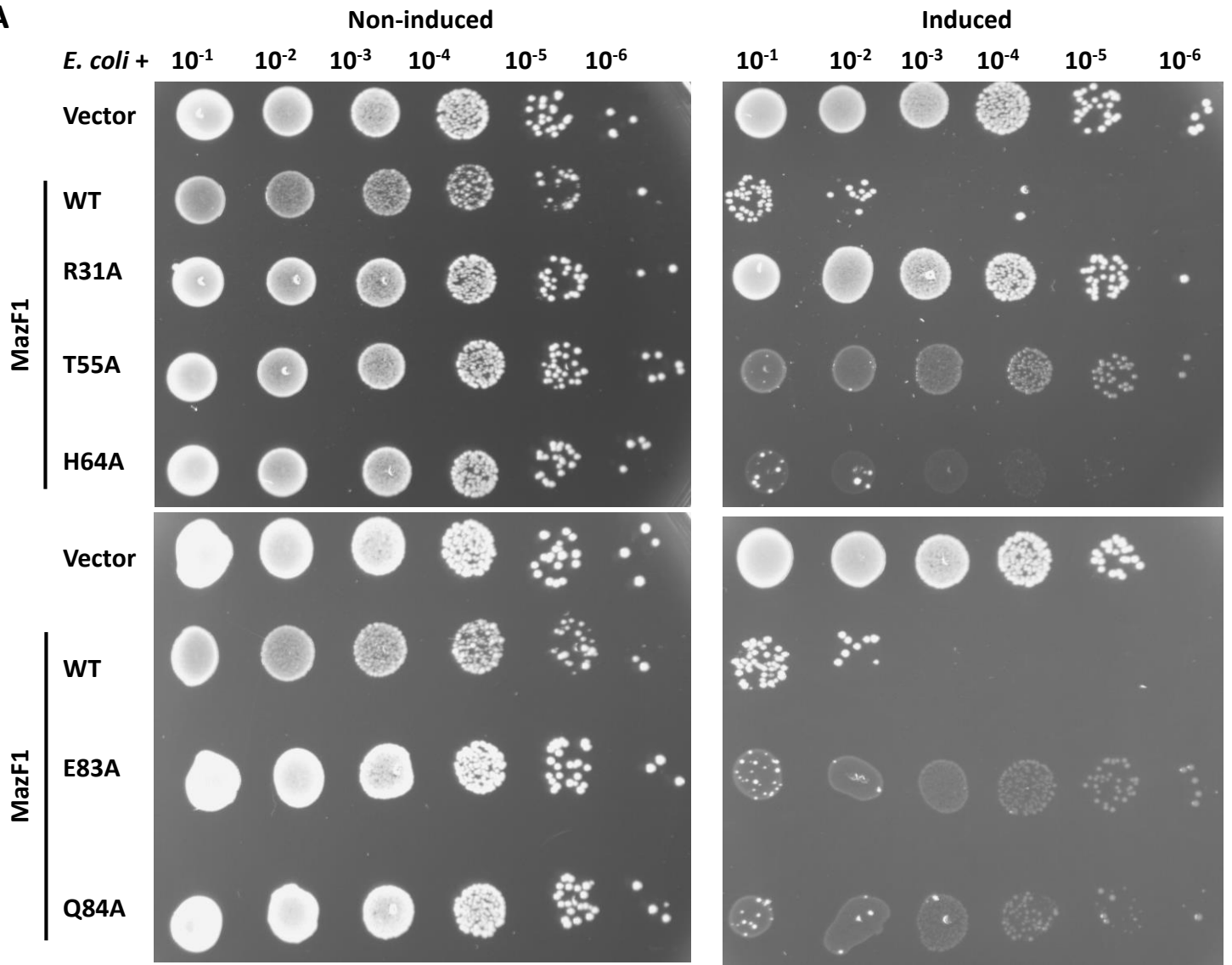**B**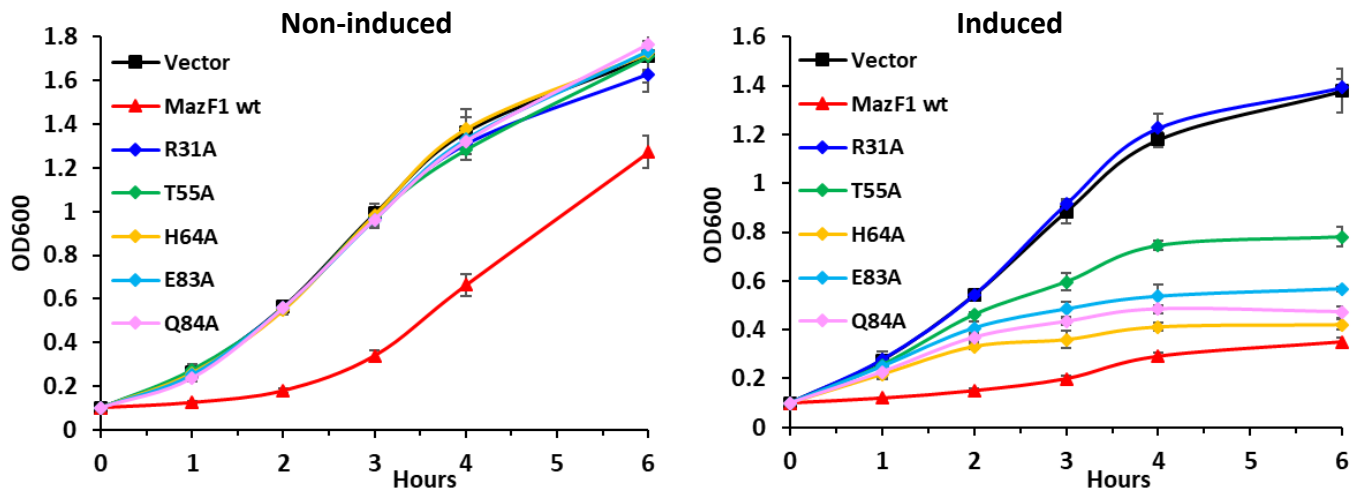

Supplement: Figure S7 — Toxicity of MazF1 point mutants in E. coli. [file aem.00681-23-s0004.pdf]

**Fig S8****A**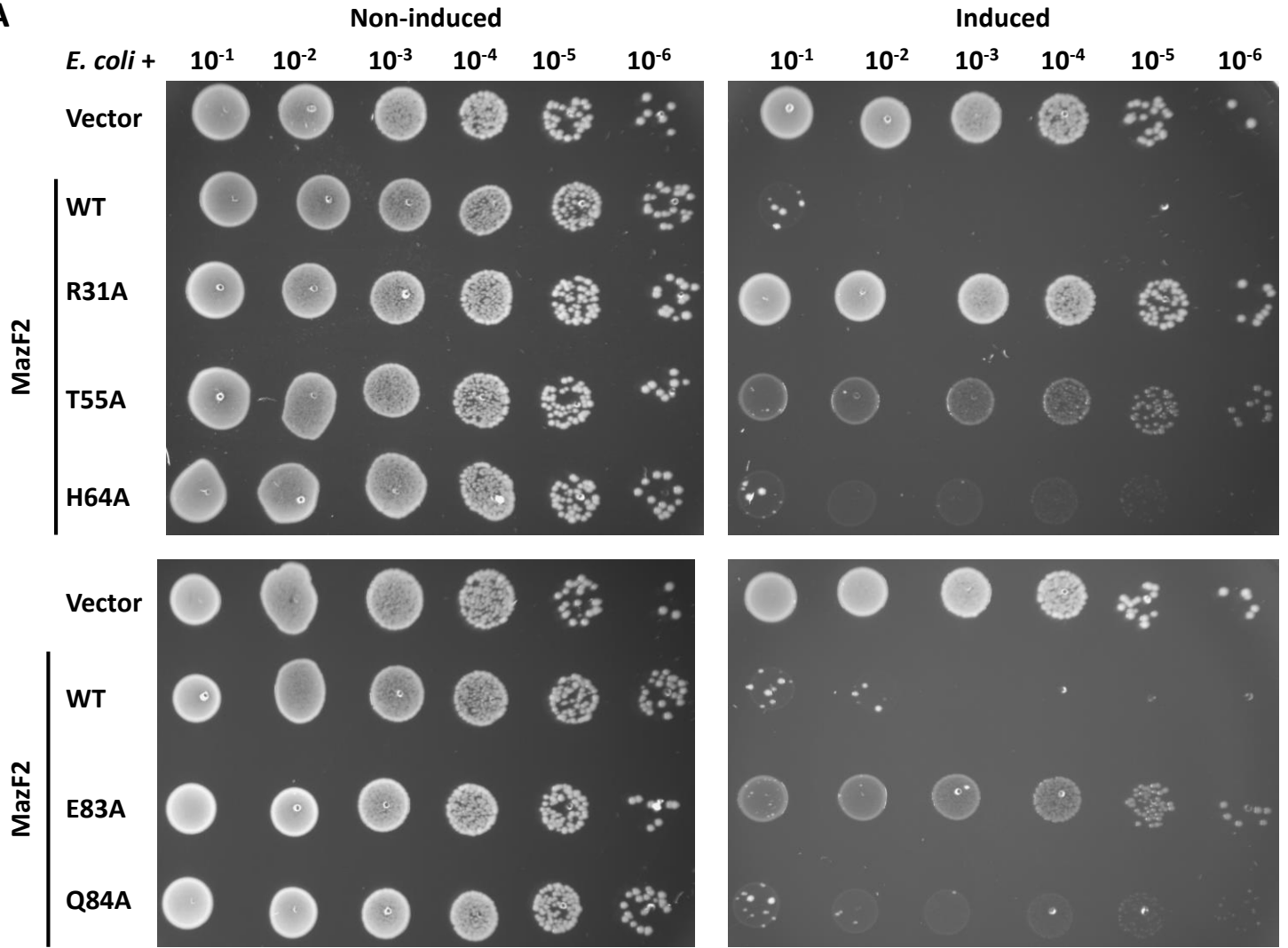**B**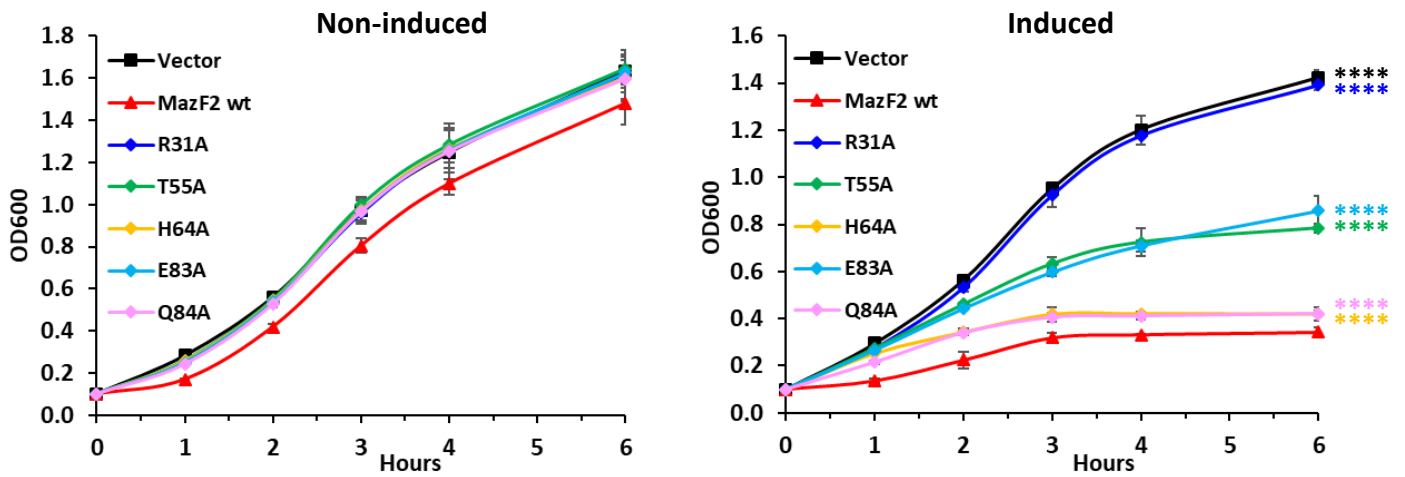

Supplement: Figure S8 — Toxicity of MazF2 point mutants in E. coli. [file aem.00681-23-s0005.pdf]

**Fig S9****A**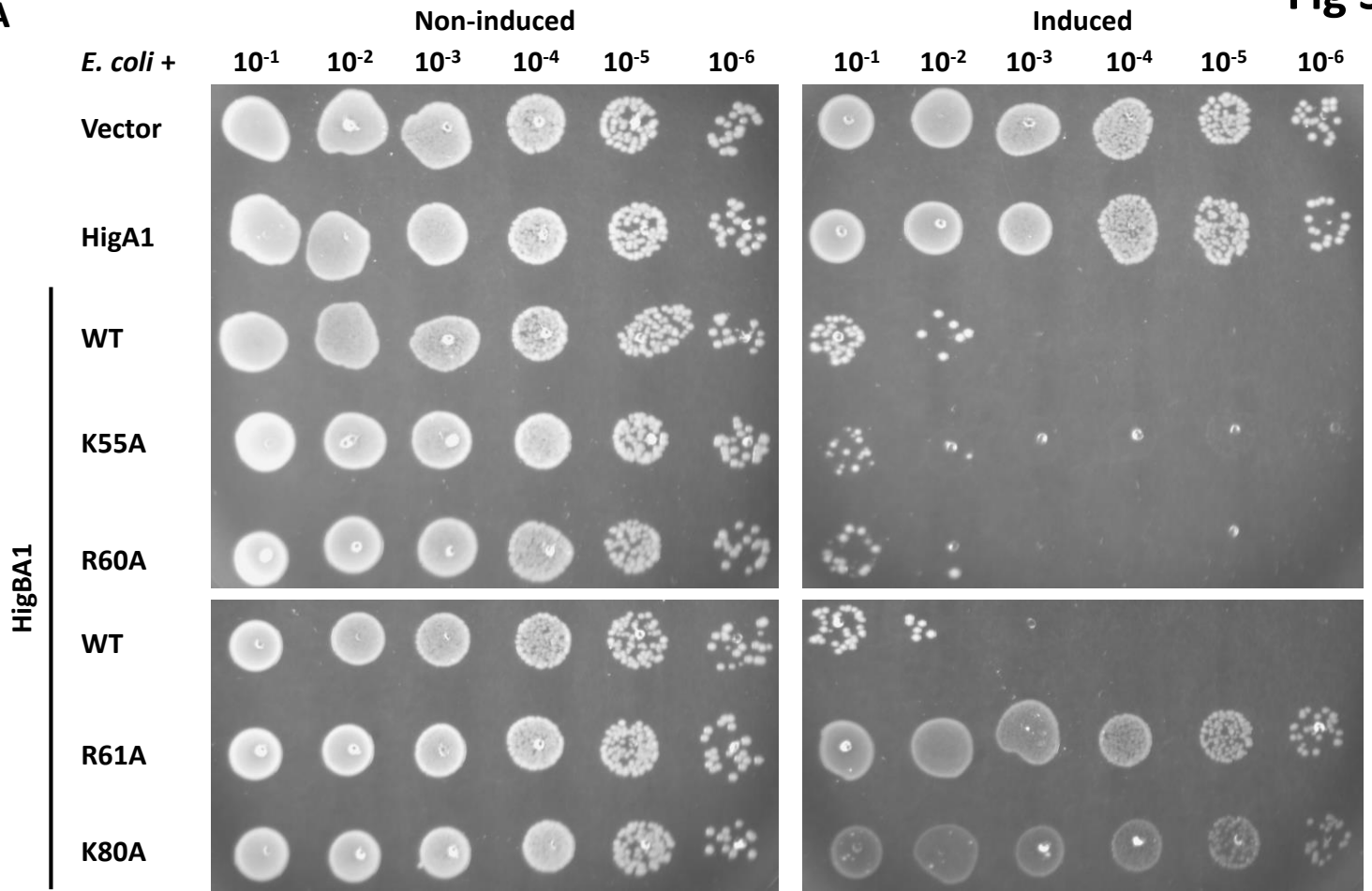**B**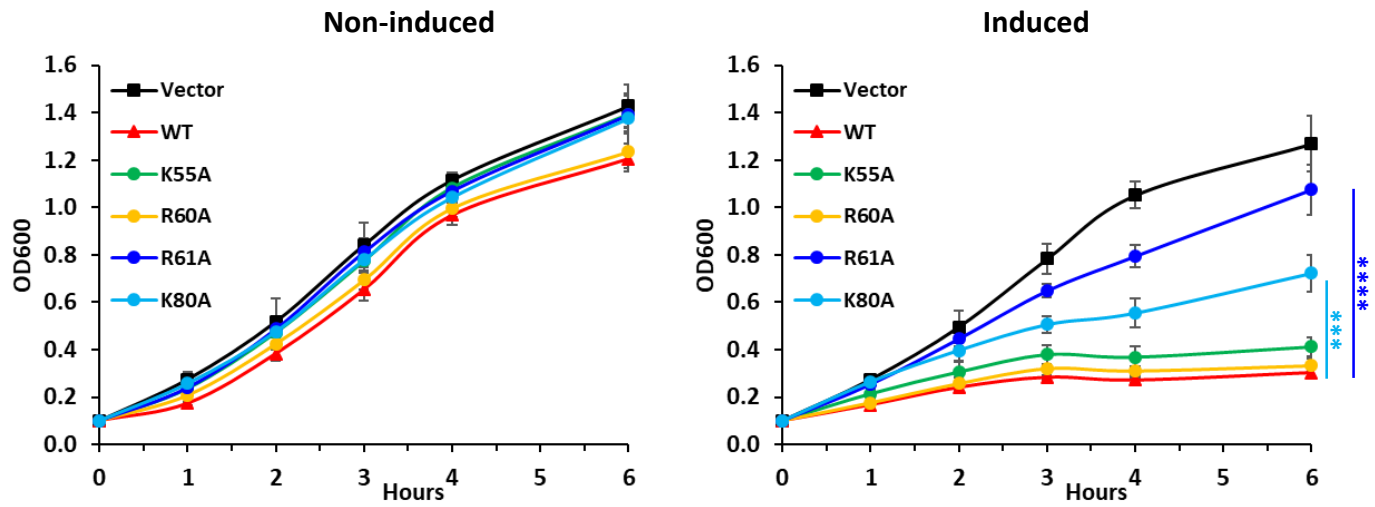**C**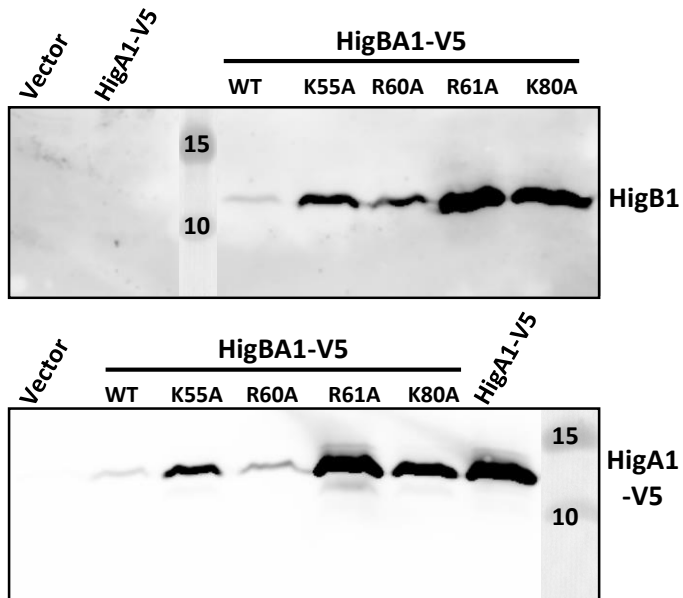**D**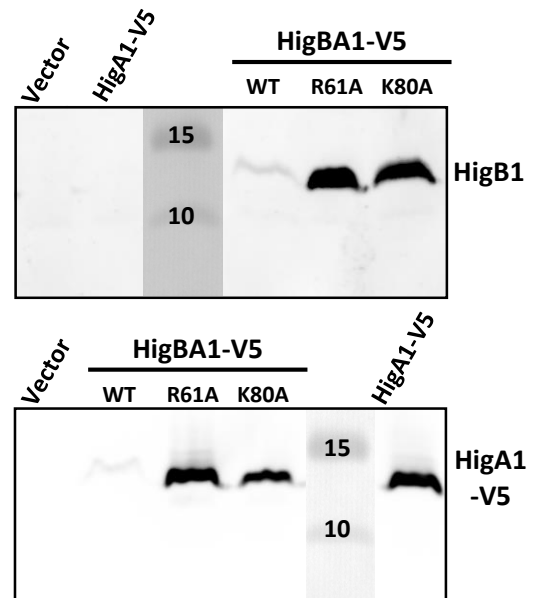

Supplement: Figure S9 — Toxicity of HigB1 point mutants in E. coli. [file aem.00681-23-s0006.pdf]

**A**

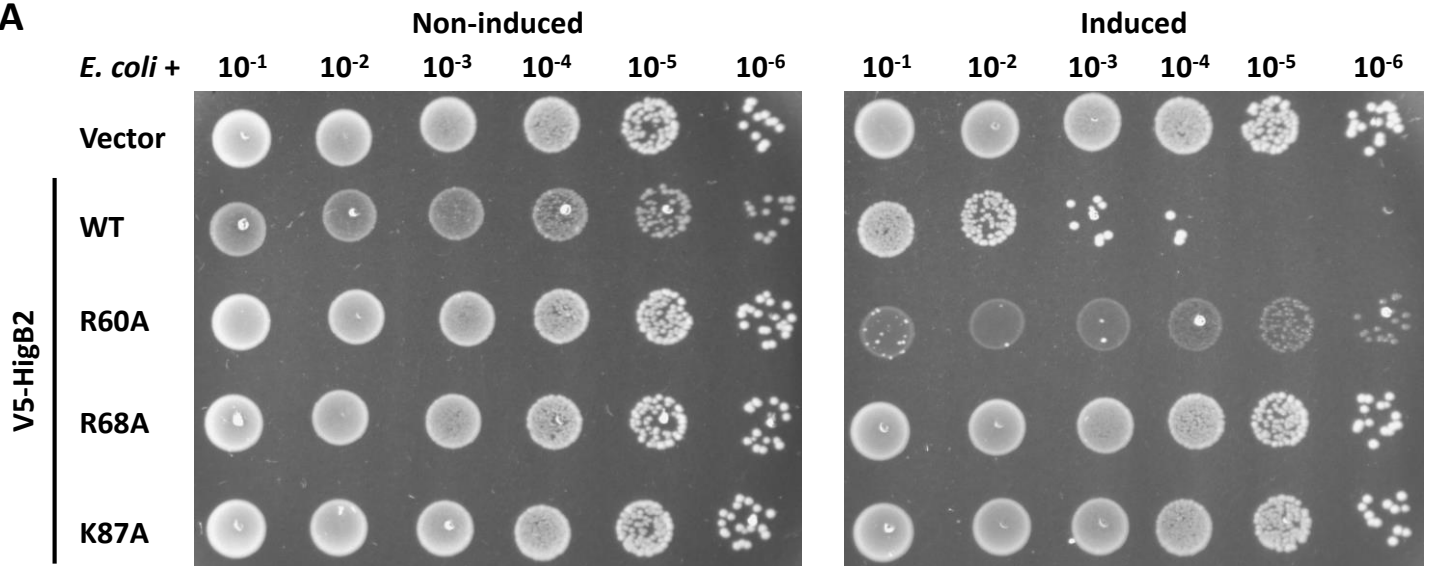

**B**

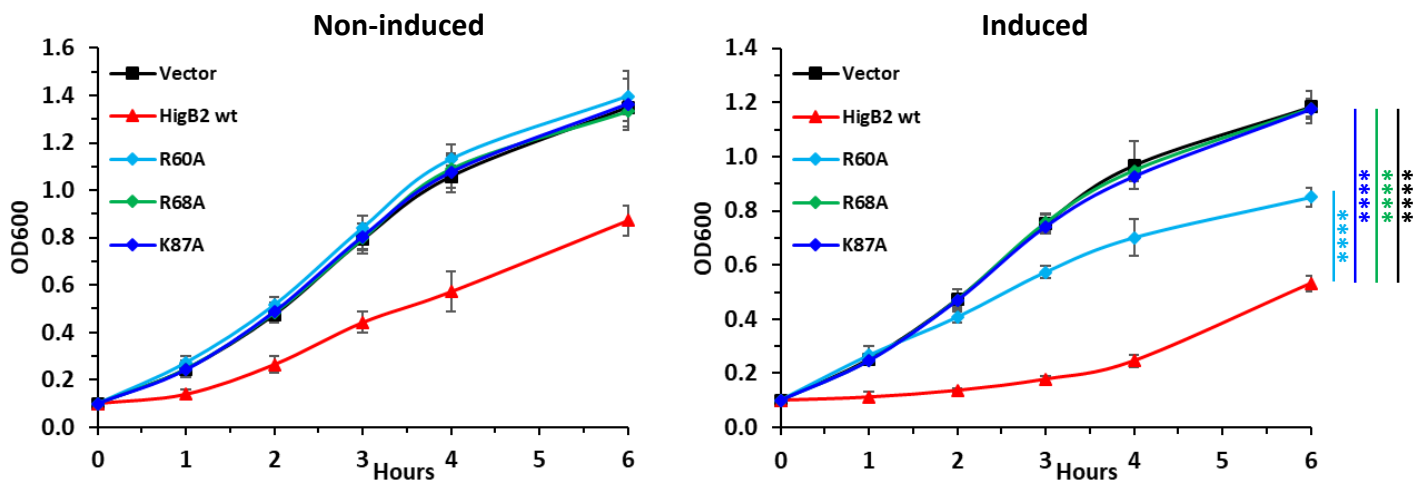

**C**

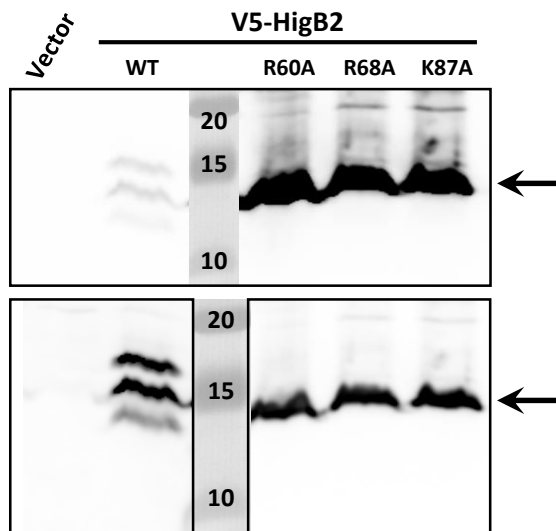

Supplement: Figure S10 — Toxicity of V5-HigB2 point mutants in E. coli. [file aem.00681-23-s0007.pdf]

A

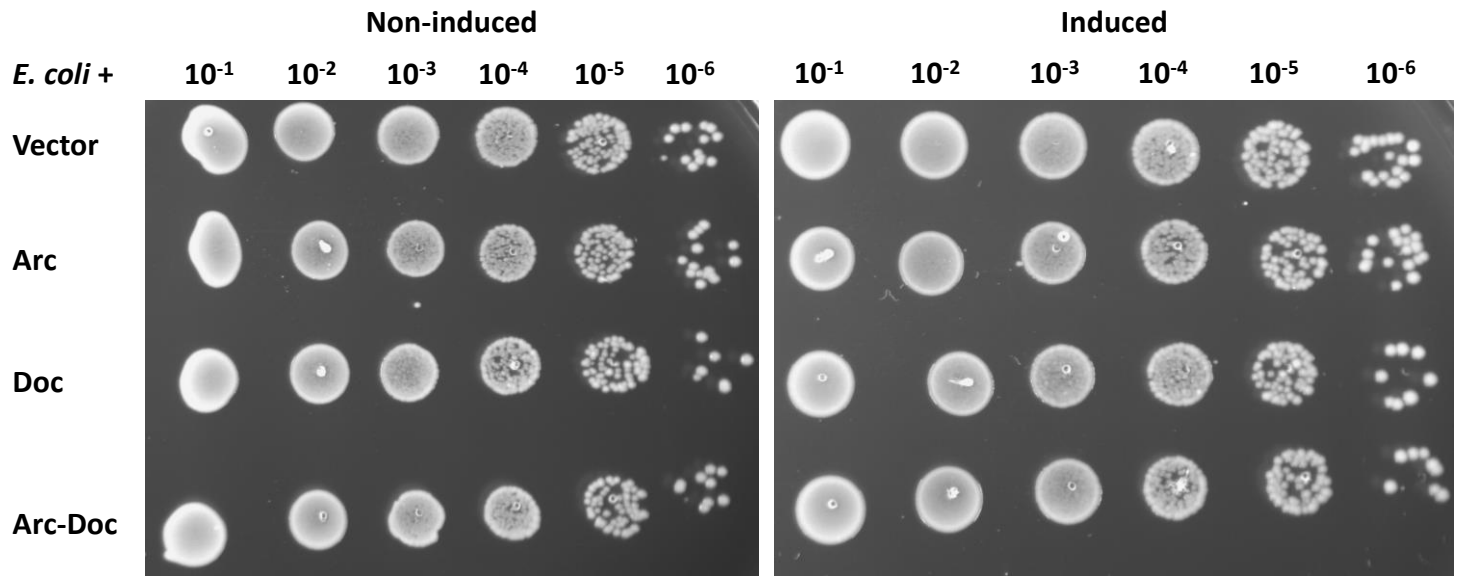

B

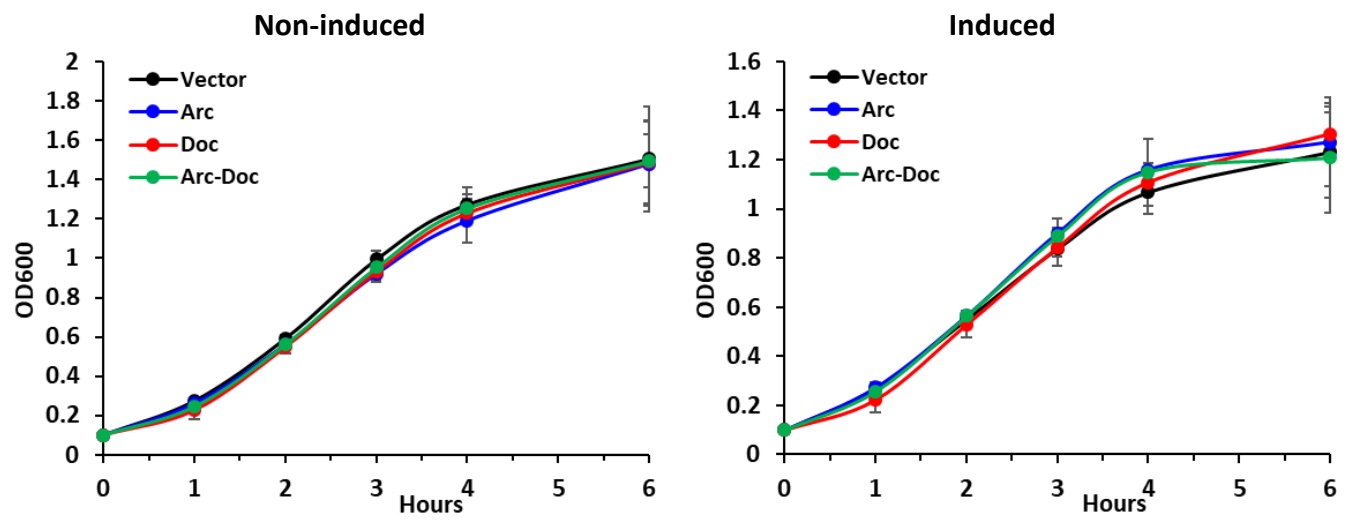

C

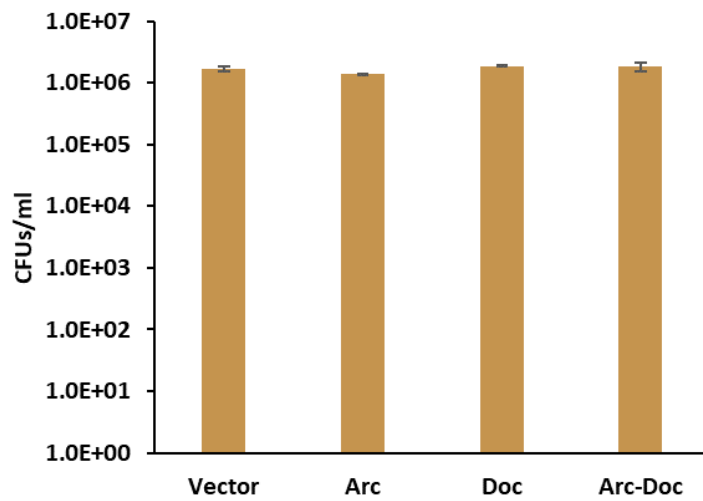

Supplement: Figure S11 — Expression of Arc-Doc in E. coli. [file aem.00681-23-s0008.pdf]

**A**

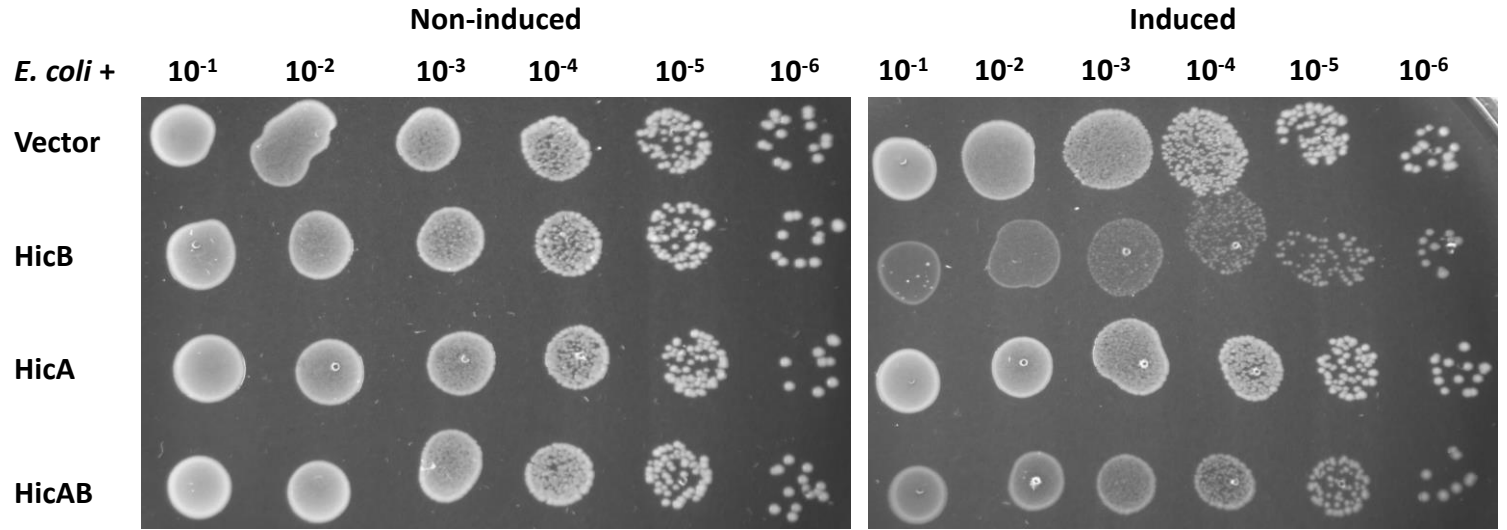

**B**

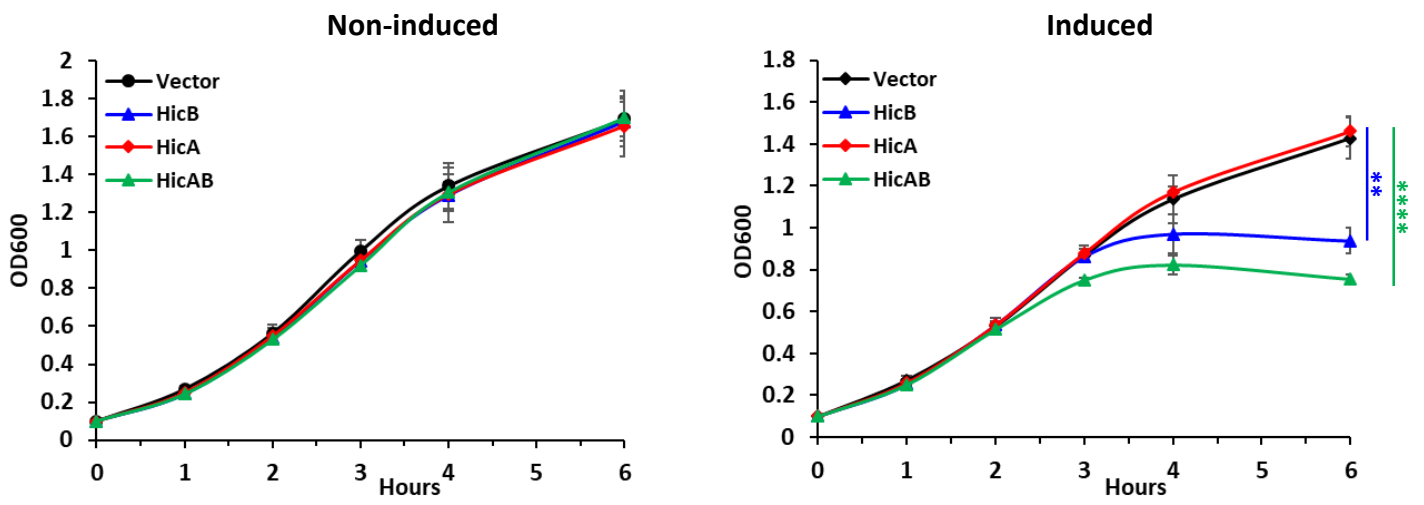

**C**

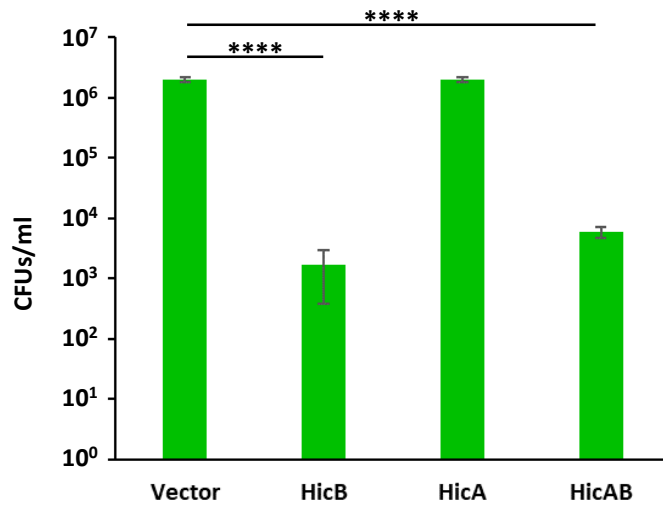

Supplement: Figure S12 — Expression of HicAB in E. coli. [file aem.00681-23-s0009.pdf]
