# Supplementary material for: The Chlamydia-related Waddlia chondrophila encodes functional type II toxin-antitoxin systems
Source: Appl Environ Microbiol. 2024 Jan 12;90(2):e00681-23. doi: 10.1128/aem.00681-23 (PMC10880633; doi:10.1128/aem.00681-23)
Supplement: Supplemental information — Legends to supplemental figures, supplemental Tables 1 and 2, Supplemental methods (plasmid construction). [file aem.00681-23-s0010.docx]

**Legends to Supplementary Figures**

**Supplementary Figure 1. HigBA1 and HigBA2 modules were likely acquired separately by *Waddlia*.**

Orthogroup phylogeny of *Waddlia* HigB1/2 (A), HigA1 (B) and HigA2 (C), derived from the chlamdb database (<https://chlamdb.ch/>). HigB1 and HigB2 belong to the same orthogroup but they are distantly related, whereas HigA1 and HigA2 belong to different orthogroups. Taken together, these data clearly indicate that the two HigBA modules were acquired separately by *Waddlia*.

**Supplementary Figure 2. MazEF1 and MazEF2 modules are the result of gene duplication in *Waddlia*.**

Orthogroup phylogeny of *Waddlia* MazE1/2 (A) and MazF1/2 HigA1 (B), derived from the chlamdb database (<https://chlamdb.ch/>). The two MazEF modules are closely related and most likely the result of a single acquisition event followed by duplication in *Waddlia*. (C) Amino acid sequence alignment of MazF1 and MazF2 with MazF from *E. coli* (Ec) and *B. subtilis* (Bs). The conserved residues shown to be important for MazF activity and targeted by site specific mutagenesis in MazF1/2 are indicated by red arrowheads.

**Supplementary Figure 3. RNA levels of *Waddlia* TA modules in ABs induced by different stress stimuli.**

RNA levels of TA encoding genes were analysed by RT-qPCR in infected cells treated with mecillinam 200 μg/ml (A), piperacillin 500 μg/ml (B), clavulanate 900 μg/ml (C), penicillin G 1000 μg/ml (D), teicoplanin 250 μg/ml (E) or vancomycin 500 μg/ml (F). RNA levels were normalised according to untreated infected cells, using the 16SrRNA as endogenous control. Infected cells were treated with drugs 8 hpi (2 hpi in the case of teicoplanin and vancomycin) and harvested 24 hpi. Results represent the mean and SD of three independent experiments. The dashed line (at fold change = 1) represents the RNA level of each gene in the untreated sample. Statistical significance was determined by paired t-test on ΔCt values. *: P<0.05; **: P<0.01; ***: P<0.001.

**Supplementary Figure 4. Expression of *Waddlia* TA modules along the infection cycle.**

RNA levels for *hicAB* (A) and *yafQ-dinJ* (B), *mazEF2* (C), *higBA2* (D) and *doc* (E) were analysed by RT-qPCR in infected cells at different time points post-infection, and normalised at 32 hpi (dashed line, fold change = 1) using the 16SrRNA as endogenous control. The *arc* gene (*wcw_0004*) was not included in the analysis due to technical issues (different pairs of primers were tested but none was suitable for qPCR). Results are the mean and SD of three independent experiments. Statistical significance was determined by paired t-test on ΔCt values. *: P<0.05; **: P<0.01; ***: P<0.001.

**Supplementary Figure 5. Expression of *Waddlia* MazF2 is deleterious to *E. coli*.**

*Waddlia* MazEF2 were expressed, alone or in combination, in *E. coli* under control of P*_ara_* on plasmid. (A) Efficiency of plating assay showing 10-fold serial dilutions of the different *E. coli* strains on LB medium with and without inducer. (B) Growth of the same strains in liquid medium, with (right) and without (left) inducer. In presence of arabinose, cells expressing *mazF2* grew significantly less than the other strains. (C) Colony forming units of the different *E. coli* strains after 6 h growth in liquid medium with inducer. In panels B and C, values represent the mean and SD of three independent experiments. Statistical significance was determined by one-way ANOVA on area under the curve (B) or CFU values (C). ****: P<0.0001.

**Supplementary Figure 6. Interactions between *Waddlia* MazE1/2 and MazF1/2 in *E. coli*.**

(A) Efficiency of plating assay showing 10-fold serial dilutions of *E. coli* strains expressing different combinations of *Waddlia* MazE and MazF from P*_ara_* on plasmid, on LB medium with and without inducer. (B) BACTH assay probing the interaction between *Waddlia* MazF1/MazF2 and MazE1/MazE2. β-galactosidase levels are represented as in Fig. 6D. Individual values of β-galactosidase activity used for panel B are listed in Suppl. Table 2.

**Supplementary Figure 7. Toxicity of MazF1 variants in *E. coli*.**

Different alleles of *Waddlia* MazF1 (shown in Fig 5) were expressed in *E. coli* under control of P*_ara_* on plasmid. (A) Efficiency of plating assay showing 10-fold serial dilutions of the different *E. coli* strains on LB medium with and without inducer. (B) Growth of the same strains in liquid medium, with (right) and without (left) inducer. Values represent the mean and SD of three independent experiments. With and without inducer, *E. coli* carrying the empty vector or expressing one of the *mazF1* mutant alleles grow more than the strain expressing WT *mazF1* (P<0.0001, as determined by one-way ANOVA on the area under the curve values for each strain compared to *E. coli* expressing WT *mazF1*).

**Supplementary Figure 8. Toxicity of MazF2 variants.**

Different alleles of *Waddlia* MazF2 were expressed in *E. coli* under control of P*_ara_* on plasmid. (A) Efficiency of plating assay showing 10-fold serial dilutions of the different *E. coli* strains on LB medium with and without inducer. (B) Growth of the same strains in liquid medium, with (right) and without (left) inducer. Values represent the mean and SD of three independent experiments. Growth of cells expressing *mazF2* R31A is indistinguishable from that of cells carrying the empty vector and *E. coli* expressing the other four *mazF2* alleles also grow more than the strain expressing WT *mazF2*. Statistical significance was determined by one-way ANOVA on area under the curve. ****: P<0.0001 compared to *E. coli* expressing WT *mazF2*.

**Supplementary Figure 9. Toxicity of HigBA1 carrying point mutations in HigB1.**

Different alleles of *Waddlia* HigB1 were expressed in *E. coli* in operon with HigA1 (carrying a C-terminal V5 tag), under control of P*_ara_* on plasmid. (A) Efficiency of plating assay showing 10-fold serial dilutions of the different *E. coli* strains on LB medium with and without inducer. (B) Growth of the same strains in liquid medium, with (right) and without (left) inducer. Values represent the mean and SD of three independent experiments. Statistical significance was determined by one-way ANOVA on area under the curve. In presence of arabinose, *E. coli* cells expressing the *higB1* R61A or K80A allele grow more than *E. coli* expressing WT *higB1*(***: P<0.001; ****: P<0.0001). Growth of the strains expressing *higBA1* alleles is significantly lower than growth of the strain carrying the empty vector (WT, R60A, K55A, K80A P<0.0001; R61A P<0.05). (C) Immunoblot against HigB1 (upper) or HigA-V5 (lower) on *E. coli* expressing HigBA1 from P*_ara_*. Samples were taken after 6 h of induction and normalised according to the OD_600nm_. (D) Immunoblot against HigB1 (upper) or HigA-V5 (lower) on the *E. coli* strains used for the promoter-probe assay shown in Fig. 7D. In panels C and D, molecular size standards are indicated in kDa.

**Supplementary Figure 10. Toxicity of V5-HigB2 variants in *E. coli*.**

Different alleles of *Waddlia* HigB2 were expressed, with an N-terminal V5 tag, in *E. coli* under control of P*_ara_* on plasmid. (A) Efficiency of plating assay showing 10-fold serial dilutions of the different *E. coli* strains on LB medium with and without inducer. (B) Growth of the same strains in liquid medium, with (right) and without (left) inducer. Values represent the mean and SD of three independent experiments. Statistical significance was determined by one-way ANOVA on area under the curve. Without inducer, *E. coli* cells expressing WT *higB2* grow significantly less than the other four strains (P<0.0001). In presence of arabinose, growth of *E. coli* cells expressing *higB2* R60A, R68A or K87A is higher than growth of *E. coli* expressing WT *higB2* (****: P<0.0001). (C) Immunoblot on *E. coli* expressing V5-HigB2 variants from P*_ara_*. Samples were taken after 6 h of induction and normalised according to the OD_600nm_. Molecular size standards are indicated in kDa. In order to obtain a similar intensity for the bands corresponding to the different variants, in the lower left panel (WT allele) was subjected to a 12-fold longer exposure than for the upper panel, whereas for the lower right panel (R60A, R68A and K87A alleles) the exposure was 10-fold shorter than for the upper panel.

**Supplementary Figure 11. Expression of *Waddlia* Doc does not affect growth in *E. coli*.**

*Waddlia* Arc and Doc were expressed, alone or in combination, in *E. coli* under control of P*_ara_* on plasmid. (A) Efficiency of plating assay showing 10-fold serial dilutions of the different *E. coli* strains on LB medium with and without inducer. (B) Growth of the same strains in liquid medium, with (right) and without (left) inducer. (C) Colony forming units of the different *E. coli* strains after 6 h growth in liquid medium with inducer. In panels B and C, values are the mean and SD of three independent experiments. Statistical analysis was carried out by one-way ANOVA on area under the curve (B) or CFU values (C).

**Supplementary Figure 12. Expression of *Waddlia* HicA does not affect growth in *E. coli*.**

*Waddlia* HicA and HicB were expressed, alone or in combination, in *E. coli* under control of P*_ara_* on plasmid. (A) Efficiency of plating assay showing 10-fold serial dilutions of the different *E. coli* strains on LB medium with and without inducer. (B) Growth of the same strains in liquid medium, with (right) and without (left) inducer. (C) Colony forming units of the different *E. coli* strains after 6 h growth in liquid medium with inducer. In panels B and C, values are the mean and SD of three independent experiments. Statistical significance was determined by one-way ANOVA on area under the curve (B) or CFU values (C). **: P<0.001; **** P<0.0001.

**Supplementary Methods**

**Plasmids construction**

Plasmids used for the BACTH experiments were constructed as follows:

pSA202 and pSA214: *mazF2* ORF was amplified with primers w1207F_Xba/w1207_stop_Kpn, digested with *Xba*I and *Kpn*I and ligated into pKT25 or pUT18C, restricted with the same enzymes.

pSA203 and pSA215: *mazE2* ORF was amplified with primers w1208F_Xba/w1208_stop_Kpn, digested with *Xba*I and *Kpn*I and ligated into pKT25 or pUT18C, restricted with the same enzymes.

pSA204 and pSA216: *mazE1* ORF was amplified with primers p0021F_Xba/p0021_stop_Kpn, digested with *Xba*I and *Kpn*I and ligated into pKT25 or pUT18C, restricted with the same enzymes.

pSA205 and pSA217: *mazF1* ORF was amplified with primers p0022F_Xba/p0022_stop_Kpn, digested with *Xba*I and *Kpn*I and ligated into pKT25 or pUT18C, restricted with the same enzymes.

pSA206 and pSA210: *mazF2* ORF was amplified with primers w1207F_Xba/w1207R_Kpn, digested with *Xba*I and *Kpn*I and ligated into pKNT25 or pUT18, restricted with the same enzymes.

pSA207 and pSA211: *mazE2* ORF was amplified with primers w1208F_Xba/w1208R_Kpn, digested with *Xba*I and *Kpn*I and ligated into pKNT25 or pUT18, restricted with the same enzymes.

pSA208 and pSA212: *mazE1* ORF was amplified with primers p0021F_Xba/p0021R_Kpn, digested with *Xba*I and *Kpn*I and ligated into pKNT25 or pUT18, restricted with the same enzymes.

pSA209 and pSA213: *mazF1* ORF was amplified with primers p0022F_Xba/p0022R_Kpn, digested with *Xba*I and *Kpn*I and ligated into pKNT25 or pUT18, restricted with the same enzymes.

pSA224 and pSA239: *higB1* ORF was amplified with primers p0002F_Spe/p0002_stop_Kpn, digested with *Spe*I and *Kpn*I and ligated into pKT25 or pUT18C, restricted with the *Xba*I and *Kpn*I.

pSA225 and pSA240: *higA1* ORF was amplified with primers p0003F_Xba/p0003_stop_Kpn, digested with *Xba*I and *Kpn*I and ligated into pKT25 or pUT18C, restricted with the same enzymes.

pSA227 and pSA242: *higA2* ORF was amplified with primers w1347F_Xba/w1347_stop_Kpn, digested with *Xba*I and *Kpn*I and ligated into pKT25 or pUT18C, restricted with the same enzymes.

pSA228 and pSA243: *higB2* ORF was amplified with primers w1348F_Xba/w1348_stop_Kpn, digested with *Xba*I and *Kpn*I and ligated into pKT25 or pUT18C, restricted with the same enzymes.

pSA229 and pSA234: *higB1* ORF was amplified with primers p0002F_Spe/p0002R_Kpn, digested with *Spe*I and *Kpn*I and ligated into pKNT25 or pUT18, restricted with the *Xba*I and *Kpn*I.

pSA230 and pSA235: *higA1* ORF was amplified with primers p0003F_Xba/p0003R_Kpn, digested with *Xba*I and *Kpn*I and ligated into pKNT25 or pUT18, restricted with the same enzymes.

pSA232 and pSA237: *higA2* ORF was amplified with primers w1347F_Xba/w1347R_Kpn, digested with *Xba*I and *Kpn*I and ligated into pKNT25 or pUT18, restricted with the same enzymes.

pSA233 and pSA238: *higB2* ORF was amplified with primers w1348F_Xba/w1348R_Kpn, digested with *Xba*I and *Kpn*I and ligated into pKNT25 or pUT18, restricted with the same enzymes.

pSA316 and pSA325: *higB2* K87A ORF was amplified with primers w1348F_Xba/w1348_stop_Kpn, digested with *Xba*I and *Kpn*I and ligated into pKT25 or pUT18C, restricted with the same enzymes.

pSA319 and pSA322: *higB2* K87A ORF was amplified with primers w1348F_Xba/w1348R_Kpn, digested with *Xba*I and *Kpn*I and ligated into pKNT25 or pUT18, restricted with the same enzymes.

pSA326 and pSA329: *higB1* R61A ORF was amplified with primers p0002F_Spe/p0002_stop_Kpn, digested with *Spe*I and *Kpn*I and ligated into pKT25 or pUT18C, restricted with the *Xba*I and *Kpn*I.

pSA327 and pSA328: *higB1* R61A ORF was amplified with primers p0002F_Spe/p0002R_Kpn, digested with *Spe*I and *Kpn*I and ligated into pKNT25 or pUT18, restricted with the *Xba*I and *Kpn*I.

**Plasmids for inducible expression in *E. coli* were created as follows:**

pSA153: *Wc_arc* ORF was amplified by PCR with primers w0003F_Nco and w0003R_Xba, digested with *Nco*I and *Xba*I, and ligated into pBAD101, restricted with the same enzymes.

pSA154: *Wc_doc* ORF was amplified by PCR with primers w0004F_Nco and w0004R_Xba, digested with *Nco*I and *Xba*I, and ligated into pBAD101, restricted with the same enzymes.

pSA155: *Wc_arc-doc* operon was amplified by PCR with primers w0003F_Nco and w0004R_Xba, digested with *Nco*I and *Xba*I, and ligated into pBAD101, restricted with the same enzymes.

pSA156: *Wc_hicB* ORF was amplified by PCR with primers w1094F_Nco and w1094R_Xba, digested with *Nco*I and *Xba*I, and ligated into pBAD101, restricted with the same enzymes.

pSA157: *Wc_hicA* ORF was amplified by PCR with primers w1095F_Nco and w1095R_Xba, digested with *Nco*I and *Xba*I, and ligated into pBAD101, restricted with the same enzymes.

pSA158: *Wc_hicAB* operon was amplified by PCR with primers w1095F_Nco and w1094R_Xba, digested with *Nco*I and *Xba*I, and ligated into pBAD101, restricted with the same enzymes.

pSA159: *Wc_dinJ* ORF was amplified by PCR with primers w1195F_Nco and w1195R_Xba, digested with *Nco*I and *Xba*I, and ligated into pBAD101, restricted with the same enzymes.

pSA160: *Wc_yafQ* ORF was amplified by PCR with primers w1196F_Nco and w1196R_Xba, digested with *Nco*I and *Xba*I, and ligated into pBAD101, restricted with the same enzymes.

pSA161: *Wc_dinJ-yafQ* operon was amplified by PCR with primers w1195F_Nco and w1196R_Xba, digested with *Nco*I and *Xba*I, and ligated into pBAD101, restricted with the same enzymes.

pSA62: *Wc_higA1* ORF was amplified by PCR with primers p0003F_Nco and p0003_V5_Sal, digested with *Nco*I and *Sal*I, and ligated into pBAD101, restricted with the same enzymes.

pSA101: *Wc_higB1* ORF was amplified by PCR with primers p0002F_Nco and p0002R_Spe, digested with *Nco*I and *Spe*I, and ligated into pBAD101, restricted with *Nco*I and *Xba*I.

pSA334: *Wc_higBA1* operon was amplified by PCR with primers p0002F_Nco and p0003_V5_Sal, digested with *Nco*I and *Sal*I, and ligated into pBAD101, restricted with the same enzymes.

pSA279: *Wc_higA2* ORF was amplified by PCR with primers w1347F_Nco and w1347R_Xba, digested with *Nco*I and *Xba*I, and ligated into pBAD22, restricted with the same enzymes.

pSA280: *Wc_higB2* ORF was amplified by PCR with primers w1348F_Nco and w1348R_Xba, digested with *Nco*I and *Xba*I, and ligated into pBAD22, restricted with the same enzymes.

pSA281: *Wc_higBA2* operon was amplified by PCR with primers w1348F_Nco and w1347R_Xba, digested with *Nco*I and *Xba*I, and ligated into pBAD22, restricted with the same enzymes.

pSA103: *Wc_mazE1* ORF was amplified by PCR with primers p0021F_Nco and p0021R_Xba, digested with *Nco*I and *Xba*I, and ligated into pBAD101, restricted with the same enzymes.

pSA193: *Wc_mazE1* ORF was amplified by PCR with primers p0021F_Nco and p0021R_Xba, digested with *Nco*I and *Xba*I, and ligated into pBAD22, restricted with the same enzymes.

pSA104: *Wc_mazF1* ORF was amplified by PCR with primers p0022F_Nco and p0022R_Xba, digested with *Nco*I and *Xba*I, and ligated into pBAD101, restricted with the same enzymes.

pSA105: *Wc_mazEF1* operon was amplified by PCR with primers p0021F_Nco and p0022R_Xba, digested with *Nco*I and *Xba*I, and ligated into pBAD101, restricted with the same enzymes.

pSA163: *Wc_mazE2* ORF was amplified by PCR with primers w1208F_Nco and w1208R_Xba, digested with *Nco*I and *Xba*I, and ligated into pBAD101, restricted with the same enzymes.

pSA197: *Wc_mazE2* ORF was amplified by PCR with primers w1208F_Nco and w1208R_Xba, digested with *Nco*I and *Xba*I, and ligated into pBAD22, restricted with the same enzymes.

pSA162: *Wc_mazF2* ORF was amplified by PCR with primers w1207F_Nco and w1207R_Xba, digested with *Nco*I and *Xba*I, and ligated into pBAD101, restricted with the same enzymes.

pSA164: *Wc_mazEF2* operon was amplified by PCR with primers w1208F_Nco and w1207R_Xba, digested with *Nco*I and *Xba*I, and ligated into pBAD101, restricted with the same enzymes.

**Site directed mutagenesis of HigB and MazF**

Point mutations were obtained by using overlap extension PCR. A first PCR was used to generate overlapping gene segments with the desired mutation. These fragments were subsequently used as template for a second PCR to obtain the full-length ORF containing the desired mutation.

For mutant alleles of HigB1:

pSA292: a first PCR was carried out with primers BAD_fwd/p0002_K55A_as and p0002_K55A/BAD_rev on pSA101 as template. The DNA fragments obtained were used as template for a second PCR with primers BAD_fwd/BAD_rev. The product generated in the second PCR was digested with *Nco*I and *Sal*I and ligated into pBAD101 cut with the same enzymes.

pSA293: a first PCR was carried out with primers BAD_fwd/p0002_R60A_as and p0002_R60A/BAD_rev on pSA101 as template. The DNA fragments obtained were used as template for a second PCR with primers BAD_fwd/BAD_rev. The product generated in the second PCR was digested with *Nco*I and *Sal*I and ligated into pBAD101 cut with the same enzymes.

pSA294: a first PCR was carried out with primers BAD_fwd/p0002_R61A_as and p0002_R61A/BAD_rev on pSA101 as template. The DNA fragments obtained were used as template for a second PCR with primers BAD_fwd/BAD_rev. The product generated in the second PCR was digested with *Nco*I and *Sal*I and ligated into pBAD101 cut with the same enzymes.

pSA295: a first PCR was carried out with primers BAD_fwd/p0002_K80A_as and p0002_K80A/BAD_rev on pSA101 as template. The DNA fragments obtained were used as template for a second PCR with primers BAD_fwd/BAD_rev. The product generated in the second PCR was digested with *Nco*I and *Sal*I and ligated into pBAD101 cut with the same enzymes.

pSA330: a first PCR was carried out with primers BAD_fwd/p0002_K55A_as and p0002_K55A/BAD_rev on pSA334 as template. The DNA fragments obtained were used as template for a second PCR with primers BAD_fwd/BAD_rev. The product generated in the second PCR was digested with *Nco*I and *Sal*I and ligated into pBAD101 cut with the same enzymes.

pSA331: a first PCR was carried out with primers BAD_fwd/p0002_R60A_as and p0002_R60A/BAD_rev on pSA334 as template. The DNA fragments obtained were used as template for a second PCR with primers BAD_fwd/BAD_rev. The product generated in the second PCR was digested with *Nco*I and *Sal*I and ligated into pBAD101 cut with the same enzymes.

pSA332: a first PCR was carried out with primers BAD_fwd/p0002_R61A_as and p0002_R61A/BAD_rev on pSA334 as template. The DNA fragments obtained were used as template for a second PCR with primers BAD_fwd/BAD_rev. The product generated in the second PCR was digested with *Nco*I and *Sal*I and ligated into pBAD101 cut with the same enzymes.

pSA333: a first PCR was carried out with primers BAD_fwd/p0002_K80A_as and p0002_K80A/BAD_rev on pSA334 as template. The DNA fragments obtained were used as template for a second PCR with primers BAD_fwd/BAD_rev. The product generated in the second PCR was digested with *Nco*I and *Sal*I and ligated into pBAD101 cut with the same enzymes.

For mutant alleles of HigB2:

pSA289: a first PCR was carried out with primers BAD_fwd/w1348_R60A_as and w1348_R60A/BAD_rev on pSA280 as template. The DNA fragments obtained were used as template for a second PCR with primers BAD_fwd/BAD_rev. The product generated in the second PCR was digested with *Nco*I and *Xba*I and ligated into pBAD22 cut with the same enzymes.

pSA290: a first PCR was carried out with primers BAD_fwd/w1348_R68A_as and w1348_R68A/BAD_rev on pSA280 as template. The DNA fragments obtained were used as template for a second PCR with primers BAD_fwd/BAD_rev. The product generated in the second PCR was digested with *Nco*I and *Xba*I and ligated into pBAD22 cut with the same enzymes.

pSA291: a first PCR was carried out with primers BAD_fwd/w1348_K87A_as and w1348_K87A/BAD_rev on pSA280 as template. The DNA fragments obtained were used as template for a second PCR with primers BAD_fwd/BAD_rev. The product generated in the second PCR was digested with *Nco*I and *Xba*I and ligated into pBAD22 cut with the same enzymes.

pSA296: *Wc_higB2* WT ORF was amplified by PCR with primers w1348F_Kpn and w1348R_Xba on pSA280 as template, digested with *Kpn*I and *Xba*I, and ligated into pSA89 (a pBAD101 derivative carrying a V5 tag between *Nco*I and *Kpn*I restriction sites), restricted with the same enzymes.

pSA297: *Wc_higB2* R60A ORF was amplified by PCR with primers w1348F_Kpn and w1348R_Xba on pSA289 as template, digested with *Kpn*I and *Xba*I, and ligated into pSA89, restricted with the same enzymes.

pSA298: *Wc_higB2* R68A ORF was amplified by PCR with primers w1348F_Kpn and w1348R_Xba on pSA290 as template, digested with *Kpn*I and *Xba*I, and ligated into pSA89, restricted with the same enzymes.

pSA299: *Wc_higB2* K87A ORF was amplified by PCR with primers w1348F_Kpn and w1348R_Xba on pSA291 as template, digested with *Kpn*I and *Xba*I, and ligated into pSA89, restricted with the same enzymes.

For mutant alleles of MazF:

pSA256: a first PCR was carried out with primers BAD_fwd/mazF_E83A_as and mazF_E83A/BAD_rev on pSA104 as template. The DNA fragments obtained were used as template for a second PCR with primers BAD_fwd/BAD_rev. The product generated in the second PCR was digested with *Nco*I and *Xba*I and ligated into pBAD101, cut with the same enzymes.

pSA257: a first PCR was carried out with primers BAD_fwd/mazF_Q84A_as and mazF_Q84A/BAD_rev on pSA104 as template. The DNA fragments obtained were used as template for a second PCR with primers BAD_fwd/BAD_rev. The product generated in the second PCR was digested with *Nco*I and *Xba*I and ligated into pBAD101, cut with the same enzymes.

pSA258: a first PCR was carried out with primers BAD_fwd/mazF_E83A_as and mazF_E83A/BAD_rev on pSA162 as template. The DNA fragments obtained were used as template for a second PCR with primers BAD_fwd/BAD_rev. The product generated in the second PCR was digested with *Nco*I and *Xba*I and ligated into pBAD101, cut with the same enzymes.

pSA259: a first PCR was carried out with primers BAD_fwd/mazF_Q84A_as and mazF_Q84A/BAD_rev on pSA162 as template. The DNA fragments obtained were used as template for a second PCR with primers BAD_fwd/BAD_rev. The product generated in the second PCR was digested with *Nco*I and *Xba*I and ligated into pBAD101, cut with the same enzymes.

pSA264: a first PCR was carried out with primers BAD_fwd/p0022_T55A_as and p0022_T55A/BAD_rev on pSA104 as template. The DNA fragments obtained were used as template for a second PCR with primers BAD_fwd/BAD_rev. The product generated in the second PCR was digested with *Nco*I and *Xba*I and ligated into pBAD101, cut with the same enzymes.

pSA265: a first PCR was carried out with primers BAD_fwd/p0022_R31A_as and p0022_R31A/BAD_rev on pSA104 as template. The DNA fragments obtained were used as template for a second PCR with primers BAD_fwd/BAD_rev. The product generated in the second PCR was digested with *Nco*I and *Xba*I and ligated into pBAD101, cut with the same enzymes.

pSA266: a first PCR was carried out with primers BAD_fwd/p0022_H64A_as and p0022_H64A/BAD_rev on pSA104 as template. The DNA fragments obtained were used as template for a second PCR with primers BAD_fwd/BAD_rev. The product generated in the second PCR was digested with *Nco*I and *Xba*I and ligated into pBAD101, cut with the same enzymes.

pSA267: a first PCR was carried out with primers BAD_fwd/w1207_T55A_as and w1207_T55A/BAD_rev on pSA162 as template. The DNA fragments obtained were used as template for a second PCR with primers BAD_fwd/BAD_rev. The product generated in the second PCR was digested with *Nco*I and *Xba*I and ligated into pBAD101, cut with the same enzymes.

pSA268: a first PCR was carried out with primers BAD_fwd/w1207_R31A_as and w1207_R31A/BAD_rev on pSA162 as template. The DNA fragments obtained were used as template for a second PCR with primers BAD_fwd/BAD_rev. The product generated in the second PCR was digested with *Nco*I and *Xba*I and ligated into pBAD101, cut with the same enzymes.

pSA269: a first PCR was carried out with primers BAD_fwd/w1207_H64A_as and w1207_H64A/BAD_rev on pSA162 as template. The DNA fragments obtained were used as template for a second PCR with primers BAD_fwd/BAD_rev. The product generated in the second PCR was digested with *Nco*I and *Xba*I and ligated into pBAD101, cut with the same enzymes.

To construct the P*_higBA1_*-*lacZ* transcriptional reporter (pSA67), a 295 bp fragment encompassing the *higBA1* promoter region was amplified by PCR with primers PhigBA1_EcoRI and PhigBA1_XbaI, digested with *Eco*RI and *Xba*I, and ligated into plac290, restricted with the same enzymes.

**Suppl. Table 1. Templates used by the Phyre2 web portal to generate secondary structure predictions of *Waddlia* putative toxin-antitoxin proteins.**

For each protein, the first 3 templates are listed. Secondary structure predictions were generated on the Phyre2 web portal between the 14^th^ and 28^th^ February 2022.

| ***Waddlia* protein** | **Template** | **Alignment Coverage** | **Confidence** | **% Identity** |
| --- | --- | --- | --- | --- |
| **HigB1** | HigB toxin from *Streptococcus pneumoniae* (PDB 6AF3 chain C) | 88% | 99.6 | 20 |
|  | RelB from *Staphylococcus aureus* (PDB 7BWF chain C) | 76% | 97.0 | 18 |
|  | HigB toxin from *Vibrio cholerae* (PDB 5JA9 chain B) | 92% | 94.7 | 15 |
| **HigA1** | Prophage Lp1 protein 11 (PDB 3B7H chain A) | 30% | 54.4 | 24 |
|  | Transcriptional regulator from *Enterococcus faecalis* (PDB 3T76 chain A) | 30% | 27.2 | 26 |
|  | SigL from *Mycobacterium tuberculosis* (PDB 3HUG chain A) | 58% | 26.3 | 14 |
| **MazF1** | MazF9 from *Mycobacterium tuberculosis* (PDB 5HJZ chain A) | 91% | 99.9 | 35 |
|  | MazF7 from *Mycobacterium tuberculosis* (PDB 5WYG chain B) | 93% | 99.9 | 35 |
|  | MazF from *Staphylococcus aureus* (PDB 4MZP chain C) | 92% | 100 | 32 |
| **MazE1** | MazE7 from *Mycobacterium tuberculosis* (PDB 6A6X chain C) | 91% | 96.1 | 21 |
|  | Transcriptional repressor from *Streptococcus agalactiae* (PDB 1EA4 chain K) | 44% | 94.4 | 30 |
|  | Transcriptional repressor from *Streptococcus agalactiae* (PDB 2CPG chain A) | 44% | 94.2 | 30 |
| **HigB2** | HigB toxin from *Streptococcus pneumoniae* (PDB 6AF3 chain C) | 88% | 100 | 26 |
|  | HigB1 from *Mycobacterium tuberculosis* (PDB 7AWK chain A) | 83% | 99.2 | 19 |
|  | YoeB from *Staphylococcus aureus* (PDB 7V6W chain A) | 71% | 96.9 | 19 |
| **HigA2** | ClgR from *Corynebacterium glutamicum* (PDB 3F52 chain A) | 56% | 98.7 | 24 |
|  | HigA toxin from *Streptococcus pneumoniae* (PDB 6AF3 chain B) | 95% | 99.4 | 16 |
|  | NGO0477 protein from *Neisseria gonorrhoeae* (PDB 3KXA chain D) | 96% | 98.7 | 14 |
| **MazF2** | MazF9 from *Mycobacterium tuberculosis* (PDB 5HJZ chain A) | 91% | 99.9 | 35 |
|  | MazF7 from *Mycobacterium tuberculosis* (PDB 5WYG chain B) | 93% | 99.9 | 36 |
|  | MazF from *Staphylococcus aureus* (PDB 4MZP chain C) | 92% | 100 | 34 |
| **MazE2** | MazE7 from *Mycobacterium tuberculosis* (PDB 6A6X chain C) | 86% | 95.9 | 20 |
|  | Transcriptional repressor from *Streptococcus agalactiae* (PDB 1EA4 chain K) | 41% | 93.9 | 30 |
|  | Transcriptional repressor from *Streptococcus agalactiae* (PDB 2CPG chain A) | 41% | 93.8 | 30 |
| **HicA** | YcfA-like from *Thermus thermophilus* (PDB 1WHZ chain A) | 71% | 97.6 | 27 |
|  | HicA toxin from *Burkholderia pseudomallei* (PDB 4C26 chain A) | 71% | 98.3 | 23 |
|  | HicA3 toxin from *Yersinia pestis* (PDB 4P78 chain D) | 70% | 97.9 | 21 |
| **HicB** | HicB3 toxin from *E. coli* K-12 (PDB 6U0I chain C) | 93% | 98.2 | 23 |
|  | Repressor from *Staphylococcus* *aureus* (PDB 6H49 chain A) | 32% | 94.4 | 28 |
|  | DNA-binding protein from *Enterococcus* *faecalis* (PDB 1UTX chain A) | 32% | 96.0 | 27 |
| **YafQ** | HP0892 protein from *Helicobacter pylori* (PDB 2OTR chain A) | 98% | 100 | 47 |
|  | RelE-like from *Helicobacter pylori* (PDB 1Z8M chain A) | 98% | 100 | 44 |
|  | YafQ from *E. coli* K-12 (PDB 4Q2U chain H) | 98% | 100 | 35 |
| **DinJ** | DinJ from *E. coli* K-12 (PDB 4Q2U chain M) | 57% | 96.9 | 24 |
|  | Pvc16 protein from *Photorhabdus asymbiotica* (PDB 6J0F chain E) | 18% | 84.8 | 53 |
|  | Afp16 protein from *Serratia entomophila* (PDB 6RAP chain E) | 20% | 81.7 | 42 |
| **Doc** | Doc protein from prophage P1 (PDB 3DD7 chain A) | 94% | 100 | 22 |
|  | Fic-domain protein from *Bacteroides thetaiotaomicron* (PDB 3CUC chain B) | 83% | 96.9 | 23 |
|  | Fic-domain protein from *Shewanella oneidensis* (PDB 3EQX chain B) | 90% | 97.1 | 19 |
| **Arc** | Arc repressor from *E. coli* (PDB 1U9P chain A) | 72% | 88.5 | 12 |
|  | ParG protein from *Salmonella enterica* (PDB 1P94 chain A) | 51% | 71.5 | 22 |
|  | AtaR protein from *E. coli* (PDB 6AJN chain F) | 43% | 62.6 | 16 |

**Suppl. Table 2. β-galactosidase values obtained for the BACTH assays.**

These data were used to generate Figure 6D, Figure 7E and Suppl. Figure 6B.

| **Plasmid 1 (T25)** | **Plasmid 2 (T18)** | **Miller Units** | **Average** | **SD** | **P value (if ≤0.05)^a^** |
| --- | --- | --- | --- | --- | --- |
| pKNT25-*mazE1* | pUT18-*mazF1* | 504  575  295 | 458 | 145 | 0.0174 |
| pKNT25-*mazF1* | pUT18-*mazE1* | 1437  1366  1571 | 1458 | 104 | <0.0001 |
| pKNT25-*mazE1* | pUT18C-*mazF1* | 1216  1620  1200 | 1345 | 238 | .  0009 |
| pKNT25-*mazF1* | pUT18C-*mazE1* | 1381  1437  1174 | 1331 | 139 | 0.0001 |
| pKT25-*mazE1* | pUT18-*mazF1* | 211  146  139 | 165 | 40 | - |
| pKT25-*mazF1* | pUT18-*mazE1* | 7876  8405  7935 | 8072 | 290 | <0.0001 |
| pKT25-*mazE1* | pUT18C-*mazF1* | 3823  4991  4406 | 4407 | 584 | 0.0002 |
| pKT25-*mazF1* | pUT18C-*mazE1* | 4384  4617  4270 | 4424 | 176 | <0.0001 |
| pKNT25-*mazE1* | pUT18-*mazF2* | 171  195  163 | 176 | 17 | - |
| pKNT25-*mazF2* | pUT18-*mazE1* | 3556  3756  3657 | 3656 | 100 | <0.0001 |
| pKNT25-*mazE1* | pUT18C-*mazF2* | 415  449  361 | 408 | 44 | 0.0004 |
| pKNT25-*mazF2* | pUT18C-*mazE1* | 187  180  290 | 219 | 62 | - |
| pKT25-*mazE1* | pUT18-*mazF2* | 131  125  128 | 128 | 3 | - |
| pKT25-*mazF2* | pUT18-*mazE1* | 8523  8672  8490 | 8562 | 97 | <0.0001 |
| pKT25-*mazE1* | pUT18C-*mazF2* | 2492  2633  2460 | 2528 | 92 | <0.0001 |
| pKT25-*mazF2* | pUT18C-*mazE1* | 8169  7815  7544 | 7843 | 314 | <0.0001 |
| pKNT25-*mazE2* | pUT18-*mazF1* | 146  152  182 | 160 | 20 | - |
| pKNT25-*mazF1* | pUT18-*mazE2* | 490  393  458 | 447 | 49 | 0.0004 |
| pKNT25-*mazE2* | pUT18C-*mazF1* | 622  385  404 | 470 | 132 | 0.011 |
| pKNT25-*mazF1* | pUT18C-*mazE2* | 174  505  478 | 386 | 184 | - |
| pKT25-*mazE2* | pUT18-*mazF1* | 136  147  140 | 141 | 6 | - |
| pKT25-*mazF1* | pUT18-*mazE2* | 4242  4524  4561 | 4442 | 174 | <0.0001 |
| pKT25-*mazE2* | pUT18C-*mazF1* | 2593  2705  2745 | 2681 | 79 | <0.0001 |
| pKT25-*mazF1* | pUT18C-*mazE2* | 5289  5228  4824 | 5113 | 253 | <0.0001 |
| pKNT25-*mazE2* | pUT18-*mazF2* | 146  153  154 | 151 | 4 | - |
| pKNT25-*mazF2* | pUT18-*mazE2* | 241  353  276 | 290 | 57 | 0.0083 |
| pKNT25-*mazE2* | pUT18C-*mazF2* | 623  388  348 | 453 | 149 | 0.0196 |
| pKNT25-*mazF2* | pUT18C-*mazE2* | 195  153  171 | 173 | 21 | - |
| pKT25-*mazE2* | pUT18-*mazF2* | 122  124  123 | 123 | 1 | - |
| pKT25-*mazF2* | pUT18-*mazE2* | 3519  2054  4260 | 3278 | 1123 | 0.0083 |
| pKT25-*mazE2* | pUT18C-*mazF2* | 1756  2162  2420 | 2113 | 334 | 0.0005 |
| pKT25-*mazF2* | pUT18C-*mazE2* | 3896  3756  3869 | 3840 | 74 | <0.0001 |
| pKT25 control | pUT18C control | 127  130  131 | 129 | 2 |  |
|  |  |  |  |  |  |
| pKNT25-*higA2* | pUT18-*higB2* | 2626  2982  2586 | 2731 | 218 | <0.0001 |
| pKNT25-*higB2* | pUT18-*higA2* | 240  242  221 | 234 | 12 | - |
| pKNT25-*higA2* | pUT18C-*higB2* | 3465  3448  3276 | 3396 | 104 | <0.0001 |
| pKNT25-*higB2* | pUT18C-*higA2* | 254  268  258 | 260 | 7 | <0.0001 |
| pKT25-*higA2* | pUT18-*higB2* | 255  256  265 | 259 | 5 | <0.0001 |
| pKT25-*higB2* | pUT18-*higA2* | 13239  12859  13526 | 13208 | 335 | <0.0001 |
| pKT25-*higA2* | pUT18C-*higB2* | 267  238  239 | 248 | 16 | - |
| pKT25-*higB2* | pUT18C-*higA2* | 3736  3114  3968 | 3606 | 441 | 0.0002 |
| pKNT25-*higA2* | pUT18-*higB2*(K87A) | 4306  4213  4034 | 4184 | 138 | <0.0001 |
| pKNT25-*higB2*(K87A) | pUT18-*higA2* | 5285  5356  4961 | 5201 | 210 | <0.0001 |

| pKNT25-*higA2* | pUT18C-*higB2*(K87A) | 862  894  849 | 868 | 23 | <0.0001 |
| --- | --- | --- | --- | --- | --- |
| pKNT25-*higB2*(K87A) | pUT18C-*higA2* | 969  982  953 | 968 | 14 | <0.0001 |
| pKT25-*higA2* | pUT18-*higB2*(K87A) | 2595  2528  2593 | 2572 | 38 | <0.0001 |
| pKT25-*higB2*(K87A) | pUT18-*higA2* | 5268  7070  5096 | 5811 | 1094 | 0.0008 |
| pKT25-*higA2* | pUT18C-*higB2*(K87A) | 288  426  329 | 348 | 71 | 0.0042 |
| pKT25-*higB2*(K87A) | pUT18C-*higA2* | 3239  3262  3298 | 3266 | 29 | <0.0001 |
| pKNT25-*higA1* | pUT18C-*higB1* | 241  317  256  284 | 274 | 34 | 0.0004 |
| pKT25-*higA1* | pUT18-*higB1* | 70  72  81  85 | 77 | 7 | - |
| pKT25-*higB1* | pUT18-*higA1* | 2953  3159  3646  2820 | 3145 | 363 | <0.0001 |
| pKT25-*higA1* | pUT18C-*higB1* | 204  268  244 | 239 | 33 | - |
| pKT25-*higB1* | pUT18C-*higA1* | 5088  4366  7514  3603 | 5143 | 1693 | 0.004 |
| pKNT25-*higA1* | pUT18-*higB1(*R61A) | 761  723  766 | 750 | 24 | <0.0001 |
| pKNT25- *higB1(*R61A) | pUT18-*higA1* | 4388  4673  4547 | 4536 | 143 | <0.0001 |
| pKNT25-*higA1* | pUT18C-*higB1(*R61A) | 228  275  166 | 190 | 33 | - |
| pKNT25- *higB1(*R61A) | pUT18C-*higA1* | 191  185  226 | 201 | 22 | - |

| pKT25-*higA1* | pUT18-*higB1(*R61A) | 579  449  334 | 454 | 123 | 0.0081 |
| --- | --- | --- | --- | --- | --- |
| pKT25- *higB1(*R61A) | pUT18-*higA1* | 2758  2700  2625 | 2694 | 67 | <0.0001 |
| pKT25-*higA1* | pUT18C-*higB1(*R61A) | 274  293  565 | 377 | 163 | 0.0457 |
| pKT25- *higB1(*R61A) | pUT18C-*higA1* | 2788  1965  1001 | 1918 | 895 | 0.0248 |
| pKT25 control | pUT18C control | 106  109  109 | 108 | 2 |  |

^a^ P value compared to negative control (empty plasmids pKT25 + pUT18C)
